# Seasonal changes in the expression of insulin-like androgenic hormone (IAG) in the androgenic gland of the Jonah crab, *Cancer borealis*

**DOI:** 10.1371/journal.pone.0261206

**Published:** 2022-02-03

**Authors:** Amanda Lawrence, Shadaesha Green, Tao Wang, Tsvetan Bachvaroff, J. Sook Chung

**Affiliations:** Institute of Marine and Environmental Technology, University of Maryland Center for Environmental Science, Baltimore, Maryland, United States of America; Shanghai Ocean University, CHINA

## Abstract

Harvesting the adult male Jonah crab, *Cancer borealis*, mainly based on the size, has become an economically significant fishery, particularly in the Southern New England region of the US since 2000. Many decapod crustacean fisheries including C. borealis rely on harvesting adult males. Understanding the size related-sexual maturity and the seasonal changes in male reproductive activity is critical for sustainable management. In other decapods, an insulin-like hormone produced by the male-specific androgenic gland (AG), called insulin-like androgenic gland factor (IAG), plays an essential role in sexual maturity. Specifically IAG is involved in developing male primary and secondary sexual characteristics including spermatogenesis. This study aimed first to identify the IAG, then examine if season influences *IAG* expression in *C*. *borealis* males. Finally, the AG transcriptome was used to test if eyestalk neuropeptides regulate IAG levels via an endocrine axis between the two endocrine tissues as established in other crustaceans. The full-length *CabIAG* sequence is 928 nucleotides long, encoding a 151 amino acid deduced sequence. The *CabIAG* identified from the AG transcriptome after eyestalk ablation was the most highly expressed gene and accounted for up to 25% of transcripts, further confirming the presence of an endocrine axis between the androgenic gland and eyestalk ganglia. This gene expression was exclusive in male *C*. *borealis* AG. The transcriptomic analysis also revealed strong upregulation of the PPOAE transcript and downregulation of proteolytic enzymes. The *CabIAG* levels differ by season, increasing AG activity in fall and possibly coinciding with high mating activity. The timing of increased AG activity correlating to mating with females should be considered for better stock management for the *C*. *borealis* population.

## Introduction

The Jonah crab, *Cancer borealis*, is geographically distributed off North America’s eastern coast from Newfoundland to Florida, in depths between 20 and 400 meters, and at temperatures ranging from 8 to 14°C [1]. Jonah crab has become economically important as the fishery has steadily grown in the US since the start of the 21^st^ century. Despite the growth of the fishery, the species remains relatively data-poor [2]. The Jonah crab fishery depends on harvesting adult males, like most other crustacean fisheries including Atlantic Snow Crab, *Chionoecetes opilio*, Tanner Crab, *C*. *bairdi*, and Red King Crab, *Paralithodes camtschaticus* [3, 4]. The current Interstate Fishery Management Plan is based on the male size and recommends harvesting *C*. *borealis* males with > 120.65 mm carapace width (CW) [5]. However, a sustainable crustacean fishery depends not only on having males in the population, but having reproductively active males. This requires us to understand male reproductive activity, i.e., mating and maturity, which vary by season.

Reproductive activity displayed in adult male crustaceans is associated with spermatogenesis and developing secondary sexual characteristics. Insulin-like androgenic gland hormone (IAG) in decapods or androgenic gland hormone (AGH) in isopods exhibit structural similarities to vertebrate insulins [6–12]. They are produced and released by the crustacean-specific androgenic gland (AG) and play an integral role in male sexual development.

The activity of AG, a male-specific endocrine gland, is thought to be regulated by neuropeptides derived from the medulla terminalis-X-organ-sinus gland complex (MTXO-SG) located in the eyestalk ganglia. For example, eyestalk ablation increases AG activity, specifically bilateral eyestalk ablation, as it increases in RNA synthesis in general in the shrimp species *Pandalus platyceros* [13]. Transcripts of *IAG* have been known to cause the hypertrophic AG of several species, including *Callinectes sapidus*, *Portunus pelagicus*, and *Macrobrachium rosenbergii* [10, 14, 15]. *IAG* is also the most abundant transcript in the AG-transcriptomes attained after eyestalk ablation of the Eastern spiny lobster, *Sagmariasus verreauxi* [16].

Water temperature determines all the physiological processes of poikilotherms; hence, reproduction (mating and spawning) and growth, in general, vary by season [17–19]. A softshell or recently molted female mates with a hard-shelled male, during which males transfer spermatophores to female spermathecae [20, 21] for internal fertilization before spawning. During the warmer months [22, 23], increased mating activity is reported in some species, whereas little information has been confirmed about seasonal reproductive activity in *C*. *borealis* males [24, 25].

This study aimed to examine if seasonal changes affect *IAG* transcript levels. To this end, the *IAG* cDNA sequence was isolated from *C*. *borealis* using 5’ and 3’ Rapid Amplification of cDNA Ends (RACE) and with gene-specific primers (GSPs) based on IAG homologs obtained from the AG transcriptome. The transcriptome of AG obtained from eyestalk-ablated and intact animals was analyzed to demonstrate if eyestalk neuropeptide(s) control IAG levels via an existing endocrine axis between these two tissues. IAG was the most abundant transcript present in the AG transcriptome of ablated animals, confirmed by qPCR assays. Finally, season significantly affected IAG transcript levels.

## Materials and methods

### Animal collection and handling

Adult male specimens of *C*. *borealis* were collected from the Mid-Atlantic Bight off the coast of Bethany Beach, Delaware and West Ocean City, Maryland (USA) within the general boundaries of 37.72–38.71°N; 74.34–74.98°W, and from waters between Martha’s Vineyard and Block Island, Rhode Island between the boundaries of 40°52’N -41°16’N and 70°20’W—71°60’W. The collection took place between September 2018—November 2019. Crabs were collected during four seasons, defined as winter (December—February); spring (March—May); summer (June—August); and fall (September—November) [19]. With samples captured predominantly as bycatch in lobster and black sea bass traps off commercial fishing vessels, the majority of adult males were obtained during the fall with an average size of 106.5 ± 5.1 mm CW. For winter (n = 5) the average animal size was 110.5 ± 4.4 mm CW, for spring (n = 3), 122.4 ± 4.3 mm CW for summer (n = 4) and 121.4 ± 1.7 mm CW for fall (n = 23). The crabs were kept in coolers with cool packs and driven on the same day as harvested, or shipped the following day to the Institute of Marine and Environmental Technology (IMET, Baltimore, MD, USA). After arrival at IMET, crabs were immediately transferred to holding tanks in a dark, climate-controlled cold room kept at 10°C in approximately 30 ppt artificial seawater. Temperature and salinity were monitored daily. After acclimating for no less than five days, the animals were chilled on ice prior to dissection for tissue sampling.

### Eyestalk ablated animal care

Adult males were transported and acclimated as previously stated. Animals with an average size of 125.6 ± 2.1 mm CW (n = 5) were chilled on ice for approximately 30 minutes prior to eyestalk ablation, and both eyestalks were removed. The bleeding was monitored to ensure animal survival. Bleeding ceased approximately 15 minutes post ablation. The animals were then returned to their tanks and kept until they were sacrificed for dissection 14 days later. All AGs used for this experiment were collected from males containing spermatophores in both the testis and vas deferens.

### RNAseq sample preparation

Androgenic glands (AG) were isolated from six adult males: three eyestalk-ablated (14 days after bilateral eyestalk-ablation) and three intact animals. Total RNA was isolated using TRIzol reagent according to the manufacturer’s protocol. RNA concentrations were measured using a NanoDrop Lite Spectrophotometer (Thermo Scientific, Waltham, MA, USA). Equal quantities of total RNA from ablated and intact samples were pooled into two groups for RNA-Seq. Samples were put on dry ice and shipped to Macrogen for RNA sequencing (www.macrogenusa.com).

### *De novo* assembly and transcriptomic analysis

Two RNAseq read sets were generated, one from the AGs of eyestalk ablated males and a second from intact males. Paired-end Illumina raw reads were first quality assessed using FastQC and trimmed using Trimmomatic with a sliding window (10 bp) trimming procedure, trimming when the mean Phred quality fell below 20 [26]. Trinity (v2.8.6) was then used to assemble all clean reads into a single transcriptome of both data sets combined (AGs of ablated and intact males) using default parameters [27]. Statistics on the single *de novo* assembly were reported in Table 1. The transcriptome was evaluated for completeness using Benchmarking Universal Single-Copy Orthologs (BUSCO). The analysis was performed by comparing it against the arthropoda_odb9 lineage dataset and used the dependencies augustus-3.3.1 (E-value threshold of 1e-05) [28]. Trimmed reads from both data sets were mapped back to the reference transcriptome using Bowtie2 v2.1.0. Transcript and gene abundances were evaluated using RSEM v1.2.28 and calculated as Transcripts Per Million (TPM) values [29]. Differential expression between intact and ablated AGs were analyzed using edgeR 3.34.0 with default parameters (dispersion value 0.1), which generated a Volcano plot (S1 Fig). Then the top 100 most differentially expressed transcripts, marked as a dotted and solid square in S1 Fig, were further analyzed with a heat map using K-means clustering and following parameters (P = 0.01, C = 2, and K = 6). This was performed after testing an elbow plot with various K values. Finally, the transcript sequences of each group were annotated using BLASTx to UniProt and the non-redundant database (S1–S3 Files).

**Table 1:** Summary statistics for the *de novo* assembly of *C*. *borealis* androgenic glands. Statistics are based on all contigs.

Statistical Summary	Number of base pairs
Contig N50	1,956
Median contig length	443
Average contig	965.57
Total assembled bases	119,172,099

### Annotation and *CabIAG*

Diamond was used to annotate and identify transcripts [30]. Potential IAG candidates were searched using BLASTx in the NCBI nr database of all IAG sequences deposited to date. Potential candidates were filtered using the following criteria: (i) long sequence hits (>100 bp), (ii) high % identity, and (iii) low E-values. The top ten selected transcriptomic sequences were translated and aligned to reference IAG sequences using BLASTx to confirm their identity, and the putative amino acid sequences were aligned using MUSCLE (MUltiple Sequence Comparison by Log-Expectation) [31]. Gene-specific primers were generated based on the most conserved regions of the alignment.

Transcript abundance (TPM) was used to determine the difference between AG transcripts in eyestalk ablated and intact males. The 100 transcripts with the highest TPM values contributing for ~50% of total reads were pulled from the RNAseq Expression and Maximization (RSEM) analysis of the reads from ablated and intact reads. The top 100 transcripts were further analyzed using BLASTx against the UniProt database with an E-value cut off of 1.0e-6 (S1 Table). The transcripts without hits were then searched using BLASTn against the NCBI database (using default settings).

### Isolating the full-length *CabIAG* cDNA sequence and phylogenetic analysis

The total RNAs from the AGs were isolated by following the manufacturer’s protocol for QIAzol lysis reagent (Qiagen). Total RNA was quantified using a NanoDrop Lite Spectrophotometer. The total RNA (~1–1.5 µg) from AGs was subjected to 5′ and 3′ RACE cDNA synthesis using the Switching Mechanism at 5′ End of RNA Template (SMART) cDNA synthesis kit (BD Biosciences) and following the manufacturer’s protocol. A two-step PCR method was employed as previously reported [10, 32] for isolating the full-length sequence of *CabIAG* cDNA. The GSPs (Table 2) were generated based on transcripts pulled from our AG transcriptome. Primers *CabIAG*-5R2 and *CabIAG*-3F1 were used to obtain an initial product, under the following conditions: 94°C for 2.5 min; annealing temperatures decreasing 2°C/cycle from 57°C to 53°C for 9 cycles; followed by 27 cycles at 94°C for 30 s; 58°C for 30 s; 68°C for 1.5 min; and the final extension at 68°C for 7 min. One microliter of the initial touch-down polymerase chain reaction product served as the template for the nested PCR, and was amplified using the nested universal primer (BD Biosciences), *CabIAG*-5R2, and *CabIAG*-3F3 to amplify a partial sequence. Bands with the expected size of ~450–550 bp were excised for DNA extraction (Qiagen), followed by subsequent cloning and sequencing as previously described [10].

**Table 2:** Primer sequences used for the isolation of the full-length *CabIAG* cDNA from the androgenic gland of male *C*. *borealis*. Primers used for qRT-PCR are noted with QF and QR. *CabNa/K-ATPase-*F and *-*R were used for a control gene.

Primer Name	Primer Sequences (5’ → 3’)
*Cab-3F1* (= QF)	TTCACCATAAAACTCTTATTTCAGGTACTGCTG
*Cab-3F2*	TCTTACACTACCAAGTCTACCTTCCATCCCCGC
*Cab-3F3*	GGGCTGACGCTGACACCAATG
*Cab-3F4*	TGCGACCTACAAACTGACCT
*Cab-5R1*	ACAATACTCCGATACCTCCTG
*Cab-5R4* (= QR)	ACACACGTAGGACATGGATCTCTG
*Cab-IAG*-st	ATGAAGTCCACAGACGAGCAA
*Cab-IAG*-end	TTATACATGCGTGTTGTTCTGAGC
*Cab-Na/K-ATPase* -QF	TGGGTCTTGTAATCTTGTGTCTCTAGG
*Cab-Na/K-ATPase* -QR	ACACACGTAGGACATGGATCTCTG

### cDNA sequencing analysis

The mRNA sequence was translated using the ExPASy translate tool (https://web.expasy.org). The predicted *CabIAG* open reading frame was then identified using the ORF finder (www.ncbi.nlm.nih.gov/orffinder/). The putative amino acid (aa) sequence was analyzed for the presence of a signal peptide utilizing SignalP 5.0 (http://www.cbs.dtu.dk/services/SignalP/). Maximum-likelihood phylogenetic analysis of *CabIAG* and all other full-length ORF IAG and AGH sequences deposited in GenBank to date was constructed using phlogeny.fr (http://www.phylogeny.fr/index.cgi). The multiple alignment was performed by MUSCLE 3.8.31, followed by phylogenetic tree construction with PHYML and tree visualization was done by TreeDyn 198.3 using mid-point rooting. A logo was generated using the predicted A and B chains trimmed from aligned IAG and AGH ORFs using WebLogo (https://weblogo.berkeley.edu/logo.cgi).

### Tissue distribution of *CabIAG*

Total RNA was extracted as described above from adult male tissues including AG, testis, eyestalk ganglia, thoracic ganglia complex, hemocytes and brain, and adult female tissues including ovary, spermatheca, eyestalk ganglia, muscle, and hepatopancreas. Approximately 1–2 µg of total RNA of each tissue sample was subjected to the first strand cDNA synthesis using the PrimeScript™ Reverse transcriptase reagent kit with gDNA eraser (TaKaRa). The spatial distribution of *CabIAG* transcripts was established using an end-point reverse-transcriptase polymerase chain reaction (RT-PCR) assay in which cDNA from each tissue (12.5 ng of total RNA equivalent) was amplified with *CabIAG-*st and *CabIAG-*end (Table 2) under the following PCR conditions: 94°C for 2.5 min, followed by 30 cycles of 94°C for 30 s, 60°C for 30 s, and 72°C for 1 min, and the final extension at 72°C for 5 min. The *Na/K-ATPase* (*CabNa/K-ATPase*) expressed in all tissue types, was amplified in the same cDNA samples using *CabNa/K-ATPase*-QF and *CabNa/K-ATPase*-QR primers also listed in Table 2.

### Effects of season and size in *CabIAG* transcripts using qPCR assay

Total RNA was extracted as described above, and cDNA synthesis was carried out using the PrimeScript^TM^ Reverse transcriptase reagent kit with gDNA eraser (TaKaRa). The *CabIAG* transcripts were measured using qPCR analysis with the primer set *CabIAG-*3F1 and *CabIAG-*5R4 to amplify the partial *CabIAG* sequence of 351 base pairs (bps) (Table 2). This product was subcloned into a pGEMT vector and the plasmid DNA served as a standard. The cDNA samples were assayed in duplicate using Fast SYBR Green Master Mix (Applied Biosystems) on an Applied Biosystems 7500 instrument.

The qRT-PCR data were presented as the mean ± SE copies/µg total RNA. Standards for *IAG* and *Na/K-ATPase* (GenBank Accession number DQ457014) were generated as described in [10, 33, 34].

Expression of *CabIAG* and *Na/K-ATPase* were quantified in AGs from males averaging 125.6 ± 2.1 mm CW for ablated animals and 116.9 ± 2.2 mm CW for intact animals, and during different times of the year with the seasonal size averages stated above.

### qPCR statistical analysis

The statistical significance for seasonal expression data was determined using a one-way ANOVA (Rstudio v3.5.2) and Tukey multiple comparison test and were both accepted at *P*<0.05. Expression of *CabIAG* between the intact and ablated groups was analyzed using a Welch two-sample *t*-test for accounting for the inconsistencies in sample size and variance. The statistical significance was accepted at the *P*<0.05 (n = 17–23 for the intact and n = 5 for the ablated). All expression data, including the size of animals, were presented as the mean ± SE (n), where n was the number of animals.

## Results

A *de novo* assembled transcriptome was generated using Trinity and included the AG of ablated and non-ablated males. The transcriptome, assembled from a total of 119,172,099 bps based on 67,206,181 Illumina paired-reads, generated 123,421 contigs or isoforms (Table 1). Half of these contigs (N50) were at least 1,956 bps in length, and the average GC content was 43.37%. The assembly generated a total of 84,041 Trinity genes. The BUSCO searches for 1066 total BUSCO groups resulted in a 93.6% completeness score.

### Differential expression analyses by TPM

The ablated and intact AGs shared the bulk of transcriptomic sequences and most highly expressed genes in common. After filtering with a TPM cut-off of 1, the *de novo* assembly of AGs from intact and ablated males shared 27,970 transcripts in common, with 9,703 Trinity genes unique to AGs of intact males and 7,387 Trinity genes unique to ablated males.

**Fig 1:**
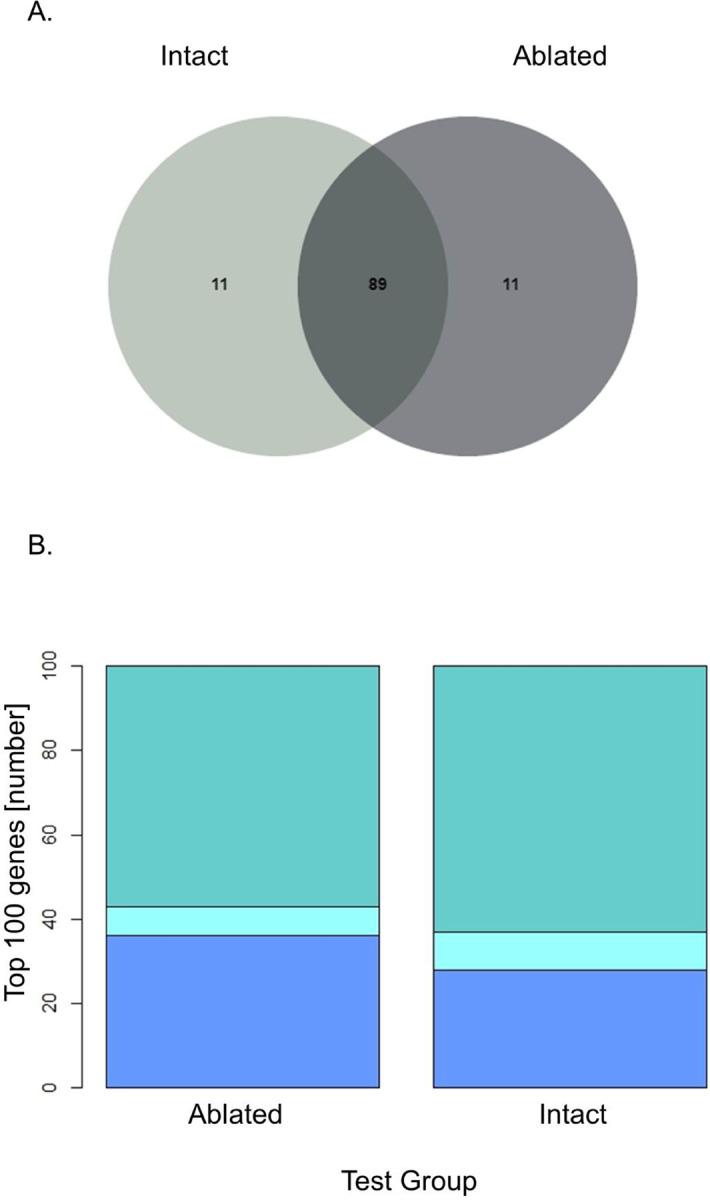
Top 100 most abundant transcripts (TPM) of intact and ablated *C*. *borealis* males. (A) A Venn diagram of the top 100 most abundant Trinity genes based on RSEM quantification of reads from intact and ablated males. (B) A stacked bar graph showing a breakdown of the top 100 most abundant genes from intact and ablated males by functional group. The top layer (dark teal) represents genes related to protein synthesis; the middle layer light teal) represents the number of genes related to energy metabolism; and the bottom layer (blue) represents genes classified as other than energy metabolism or protein synthesis.

A Venn diagram (using jvenn.toulouse.inra.fr) was generated for the 100 most highly expressed genes of both intact, and ablated datasets (S1 Table) [35]. The two datasets shared 89 genes in common, while both datasets possessed 11 unique genes (Fig 1A). The sequences were sorted into three functional groups based on the gene ids: mitochondrial sequences, protein synthesis and other (Fig 1B). There were six more genes involved in protein synthesis for intact males than ablated males, while there were 10 additional genes categorized as other in ablated males [Fig 1B]. Generally, ribosomal proteins were among the most highly expressed genes in the protein synthesis category accounting for 59 shared genes. On the other hand, only four more genes were mitochondrial or involved in energy metabolism for intact males than for ablated males when examining the 100 most highly expressed genes (Fig 1B). Aside from *IAG*, other highly expressed genes that were upregulated in ablated male AGs included alpha 2-tubulin with a 1.7-fold increase, an intracellular cholesterol transporter with a 3.8-fold increase and a phenoloxidase-activating enzyme (PPOAE) with a 4.2-fold increase in expression (S1 Table).

### Differential expression by edgeR analysis

**Fig 2:**
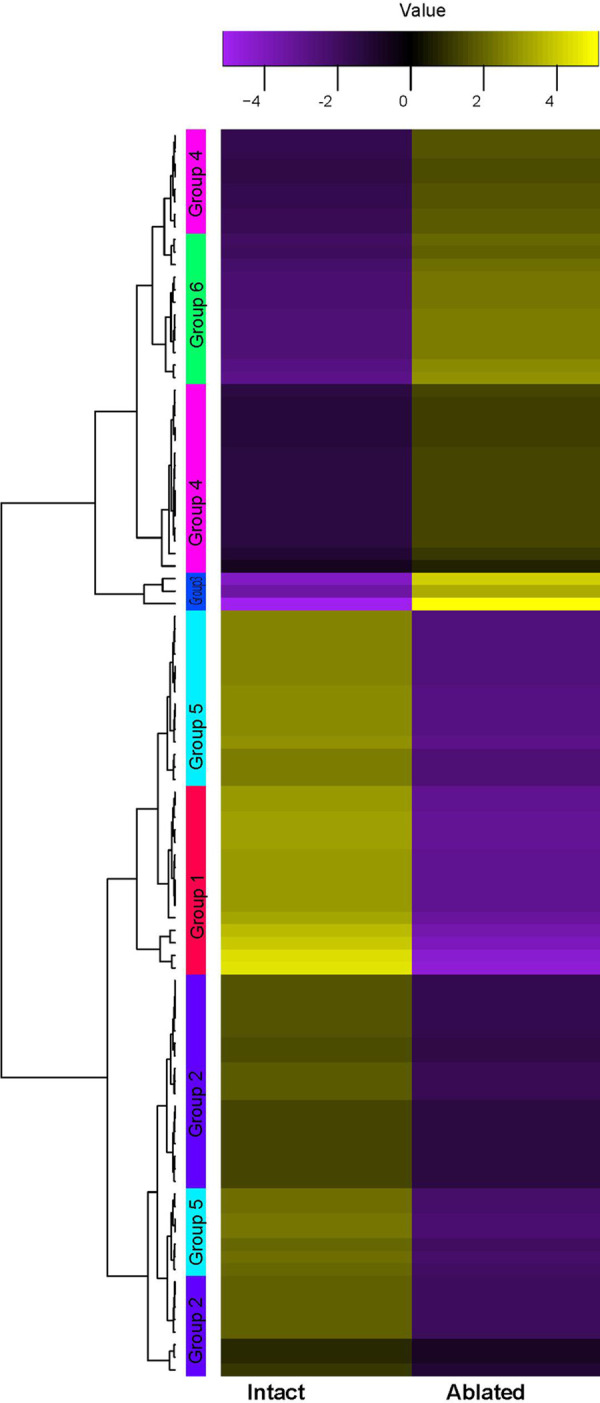
A heat map generated using K-means (K = 6) clustering of the top 100 most differentially expressed transcripts by edgeR. K = 6 was selected after testing an elbow plot with various K values. The sequence identities of all six groups were listed as S1 File, together with differential expression data of logFC and P values as S2 File, and the annotation of the sequences using UniProt and BLASTx nr as S3 File.

**Fig 3:**
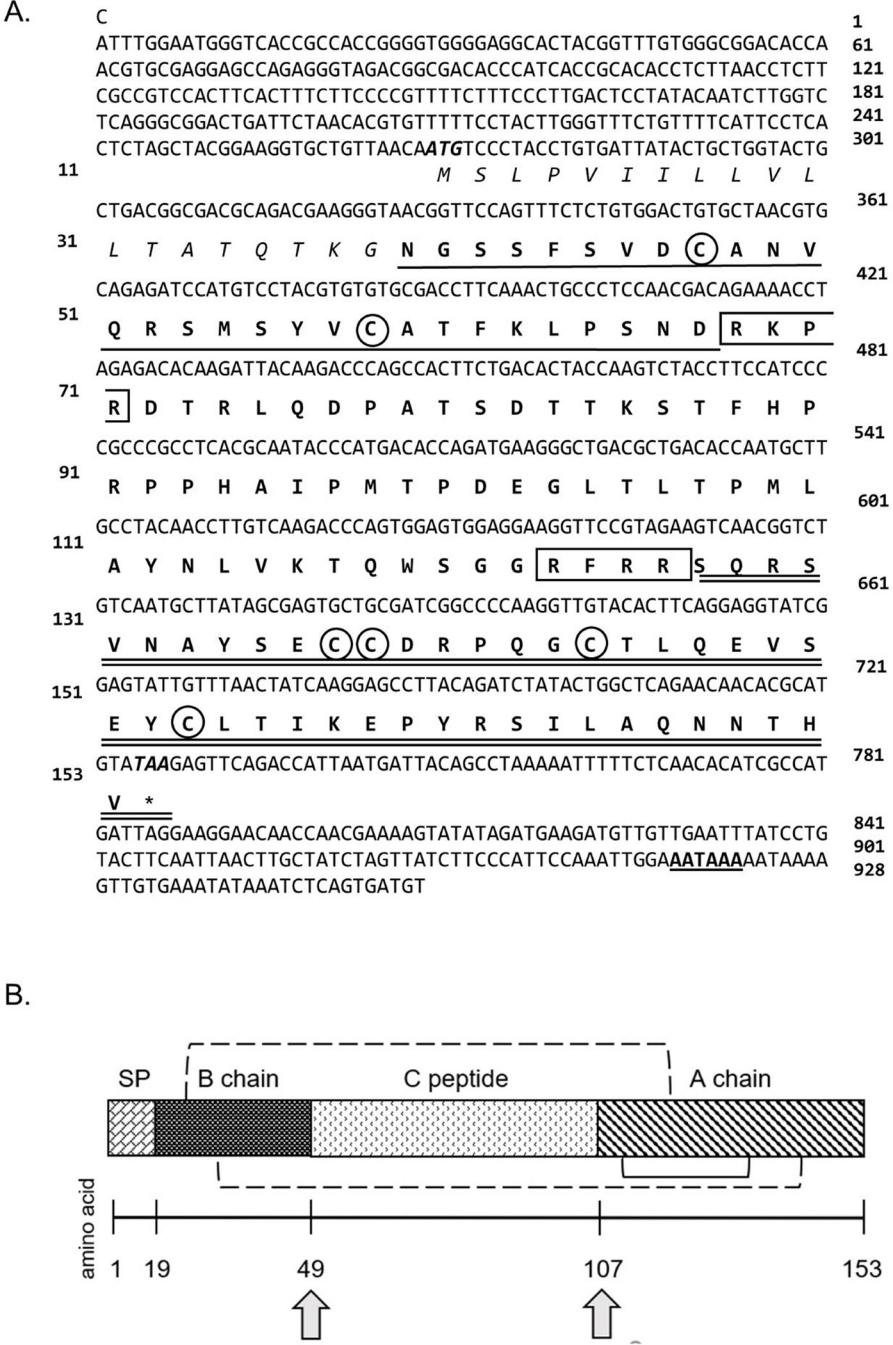
*CabIAG* cDNA sequence annotation and predicted structure. (A) The full-length cDNA and deduced amino acid sequence of *CabIAG* from the androgenic gland of male *C*. *borealis* (GenBank Accession No. MW207169). The nucleotide position is on the right margin and the amino acid position is on the left margin, respectively. The predicted start (ATG) and stop (TAA) are bolded and italicized in the predicted ORF region. The signal peptide is italicized and precedes the B chain, which has a single underline. The C peptide is flanked between the B and A chains and is double-underlined. The six cysteines, conserved in all crustacean IAGs, are noted with a circle. Two predicted cleavage sites (RKPR/RFRR) are boxed, and a putative polyadenylation site (AATAAA) is bolded and single-underlined in the 3’ UTR. (B) Schematic of the *CabIAG* open reading frame structure including the signal peptide, B chain, C peptide and A chain. The predicted cleavage sites are represented by gray arrows. The dashed brackets represent predicted inter-disulfide bridge formation, and the solid bracket represents a predicted intra-disulfide bridge; each bridge formed by conserved cysteine residues. The number line below represents the number of amino acids.

Differential expression of intact and ablated AG transcriptomes were analyzed using edgeR and was shown with a Volcano plot (S1 Fig). The top 100 most differentially expressed transcripts were marked in S1 Fig. They were then clustered using *K*-means (K = 6) as shown in the heat map (Fig 2). Of the six clusters, groups 3, 4, and 6 were upregulated and shown in S1 Fig: 3 (circled in blue) and groups 1, 2, and 5 were downregulated. Among the three upregulated groups, group 4 are first clustered together with group 6, then out grouped with group 3 (Fig 2). For three downregulated groups, group 1 was first clustered with group 5 but separated from group 2 (Fig 2).

The most differentially upregulated transcript was also identified in the ablated AG as PPOAE belonging to group 3 (Fig 2) with a logFC value of 11.46 at *P* = 4.22E-29 (S1 Fig with *). The two most differently downregulated transcripts were trypsin-like enzyme and chymotrypsin (group 1. Fig 2 and S1 Fig), with a logFC value of -15.01 at *P* = 4.75E-25 (S1 Fig with **) and a logFC value of -14.75 at *P* = 2.97E-24, respectively (S1 Fig with ***).

### Sequence analysis of *CabIAG* cDNA

The full-length cDNA sequence of 928 nucleotides (nt) *CabIAG* (GenBank accession number MW207169) was isolated from *C*. *borealis* adult male AG RNA (Fig 3A). The cDNA sequence comprised a 268 nt 5’ untranslated region (UTR), a 351 nt open reading frame (ORF), and a 201 nt 3’ UTR. The *CabIAG* ORF encoded for 117 amino acids, including a 19 aa signal peptide: MSLPVIILLVLLTATQTKG, identified (*P* = 0.628) using SignalP 5.0 (http://www.cbs.dtu.dk/services/SignalP/) (Fig 3A).

A schematic diagram of the ORF depicts the putative *CabIAG* sequence with insulin-like B and A chains containing six conserved cysteine residues, which surround the C peptide (Figs 3B and S2). These cysteine residues likely form three disulfide bridges (two interchains: C_B9_ and C_A12_ and C_B20_ and C_A27_, and one intrachain: C_A11_ and C_A18_) (Fig 3B). The predicted B chain (33 aa) begins immediately after the end of the signal peptide (represented by a single underline in Fig 3B). The B chain is followed by the C peptide 55 aa) (represented with no underline), which is then followed by the A chain (45 aa) and represented with a double underline. Two cleavage sites are also present at the end of chains B and the C peptide, respectively (boxed) (Fig 3B). Additionally, a polyadenylation site is predicted in the 3’ UTR (underlined and bolded) (Fig 3A).

### Phylogenetic tree

**Fig 4:**
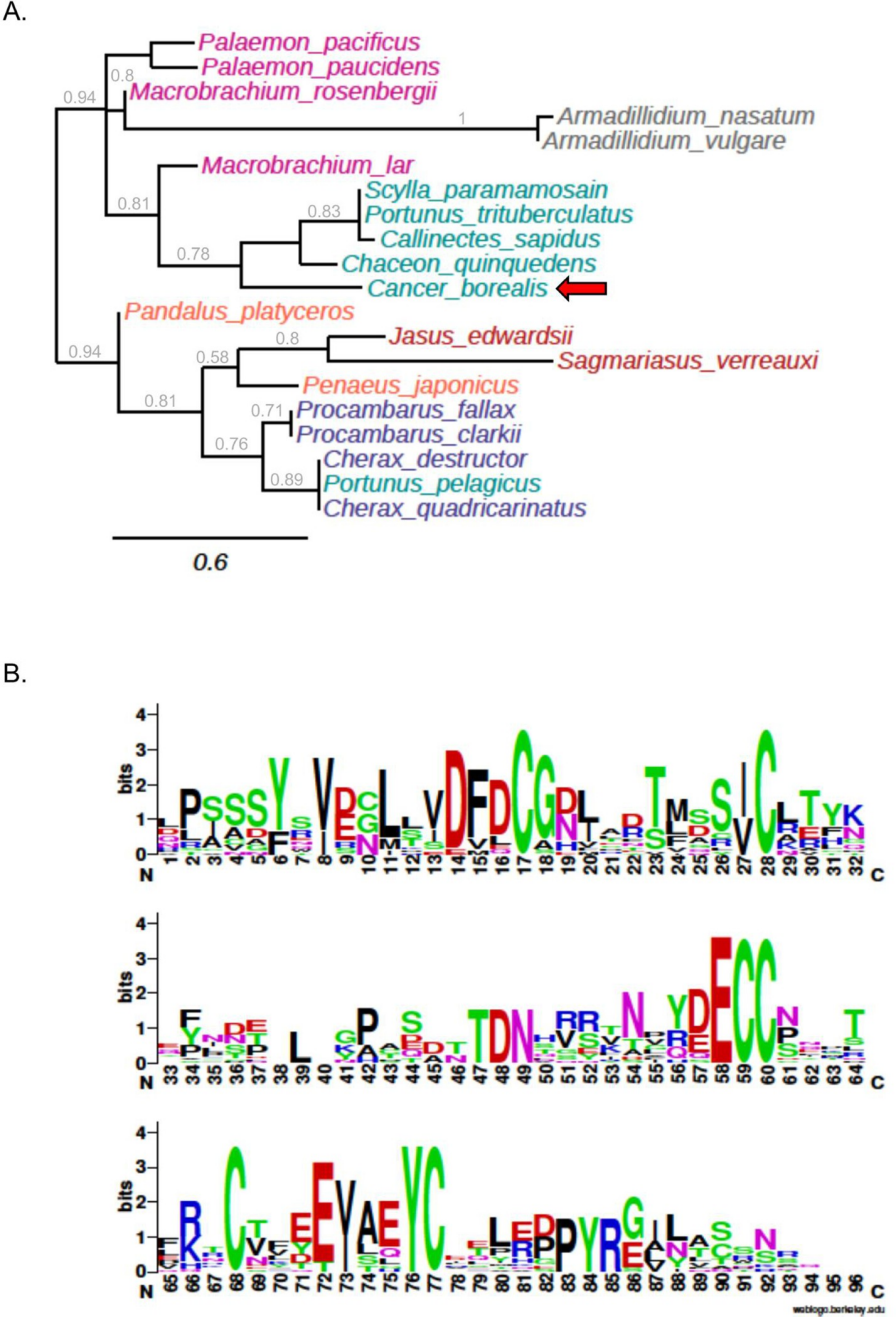
Phylogenetic tree of IAGs and AGHs and sequence logo. (A) A phylogenetic tree representing distance and relationships between IAG and AGH amino acid sequences. *Cancer borealis* is represented with a red arrow. The scale bar indicates the number of amino acid sequence substitutions per site. The branch support values above 0.5 are presented in grey. The sequences represented include: *Armadillidium vulgare*, BAA86893.1; *Armadillidium nasatum*, KAB7497038.1; *Cancer borealis*, MW207169; *Chaceon quinquedens*, ASA45642.1; *Portunus trituberculatus*, QCH40811.1; *Callinectes sapidus*, AEI72263.1; *Scylla paramamosain*, AIF30295.1; *Pandalus platyceros*, ASM94212.1; *Palaemon pacificus*, BAJ84109.1; *Palaemon paucidens*, BAJ84108.1; *Macrobrachium lar*, BAJ78349.1; *Macrobrachium rosenbergii*, ACJ38227.1; *Jasus edwardsii*, AIM55892.1; *Sagmariasus verreauxi*, AHY99679.1; *Penaeus japonicus*, BAK20460.1; *Procambarus fallax*, ASM94213.1; *Procambarus clarkia*, ALX72789.1; Cherax destructor ACD91988.1; *Portunus pelagicus*, ADK46885.1; *Cherax quadricarinatus*, ABH07705.1. The colors represent the following groups: shrimp species are magenta; isopod species are grey; crab species are green; prawn species are light orange; lobster species are dark orange; crayfish species are purple. (B) A sequence logo of MUSCLE 3.8.31 aligned decapod IAG and isopod AGH deduced amino acid sequences for A and B chains was generated using https://weblogo.berkeley.edu/logo.cgi. The Y-axis describes the amount of information in bits*, amino acid size is proportional to frequency, and the X-axis shows the position in the alignment.

Maximum-likelihood phylogenetic analysis of decapod IAG and isopod AGH ORFs including *CabIAG* was performed and displayed using midpoint rooting (Fig 4A). The phylogenetic analysis produced two main, and strongly supported branches (0.94 branch support value). One branch includes isopod, shrimp, and all crab species except for *P*. *pelagicus*. The second branch includes lobster, crayfish, and prawn species. The *CabIAG* most closely aligns with *Chaceon quinquedens* and clusters with other crab species forming a moderately supported clade with a 0.78 branch support value. Overall, isopod, lobster crayfish and most crab and shrimp species cluster together to form clades supported by moderate to strong branch support values. All shrimp and crayfish species are confined strongly to moderately supported branches (0.94 and 0.76, respectively). However, *Machrobrachium lar* and *Penaeus japonicus* are grouped with crab and crayfish, respectively, with both supported by branch values of 0.81. One anomaly to note is that *P*. *pelagicus*, a crab species, is located on a completely separate branch, clustered among the crayfish, and moderately supported by a branch value of 0.76.

The multiple sequence alignment of crustacean IAGs contained typical signatures of insulin. The logo of predicted IAG A and B chains reveals complete conservation of the six cysteines predicted to form three disulfide bridges (Fig 4B). There are also conserved features in the amino acids around the cysteines except for the 5^th^ located at position 68 (Fig 4B). Taking a look the full sequence, the C peptide was most variable except the regions around the R-X-X-R predicted cleavage sites (S2 Fig). Highly conserved predicted cleavage sites are present at positions 88–91 and 193–194 and the C-terminal end of predicted the C peptide and B chain. There is much higher conservation among A and B chains, while conservation in the C peptide is relatively low (S2 Fig).

### Tissue distribution

**Fig 5:**
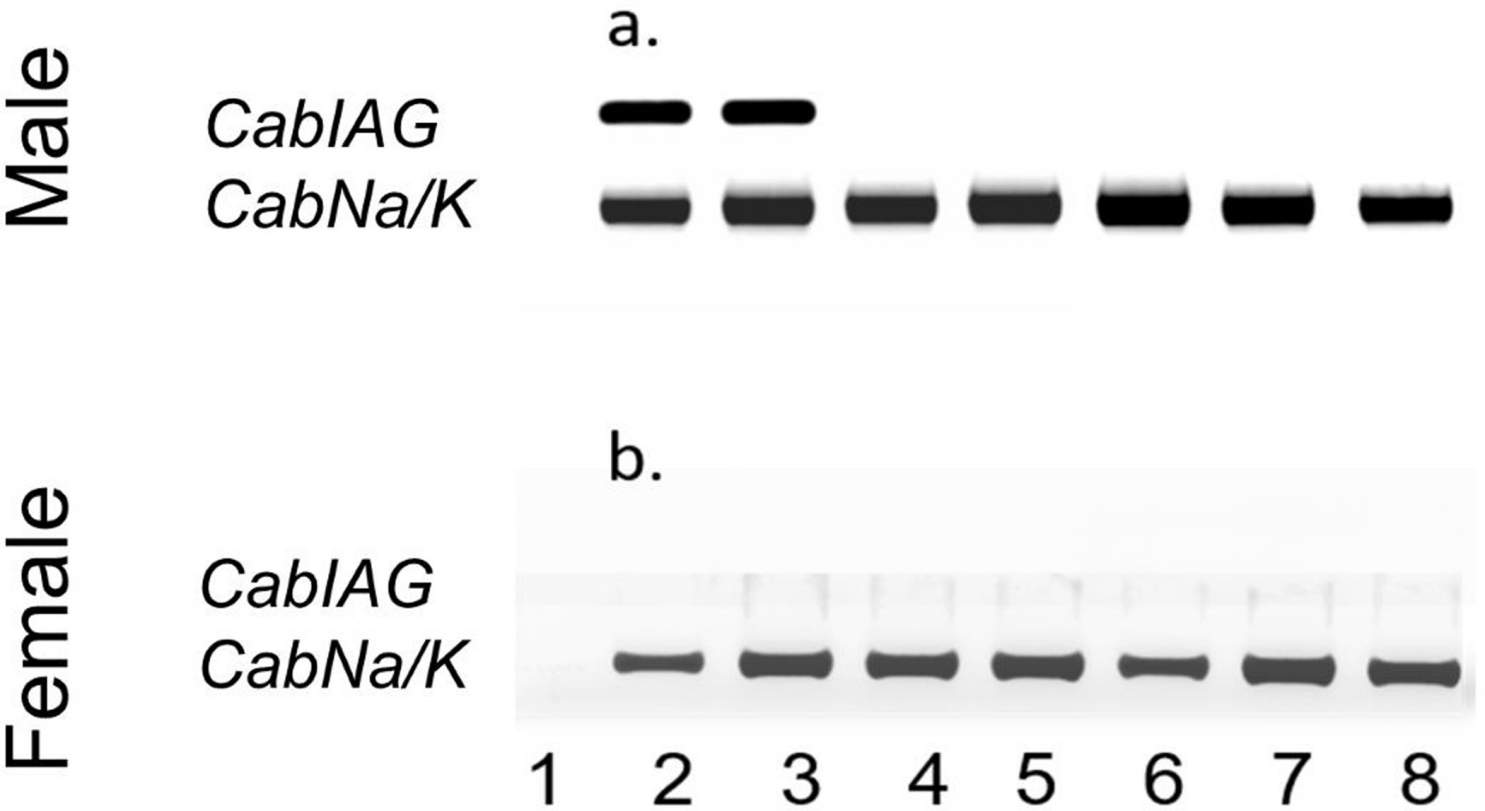
Tissue distribution of *CabIAG* and *CabNa/K-ATPase* expression in *C*. *borealis* males and females. Spatial expression of *CabIAG* in adult (a) male (b) female tissues using an end-point PCR. Male: 1: no template control; 2: AG; 3: AG from an eyestalk-ablated individual; 4: eyestalk ganglia; 5: thoracic ganglia complex; 6: brain; 7: hemocytes; 8: testis. Female: 1: no template control; 2: spermatheca; 3: eyestalk ganglia; 4: thoracic ganglia complex; 5: chela muscle; 6: hemocytes; 7: hepatopancreas; 8: ovary. *CabNa/K-ATPase* was amplified using the same tissue cDNA samples as a reference gene.

The spatial distribution of *CabIAG* expression was examined by amplifying the ORF region in the cDNAs of various tissues of adult male and female *C*. *borealis* and showed the expression to be specific to the male. However, these tissue cDNA samples had consistent *Na/K-ATPase* expression. The only tissue expressing *CabIAG* is the AG, which shows similar expression patterns in both eyestalk-ablated and intact male AGs (Figs 5 and S3).

### *CabIAG* transcripts in the AGs obtained from intact and ablated males

**Fig 6:**
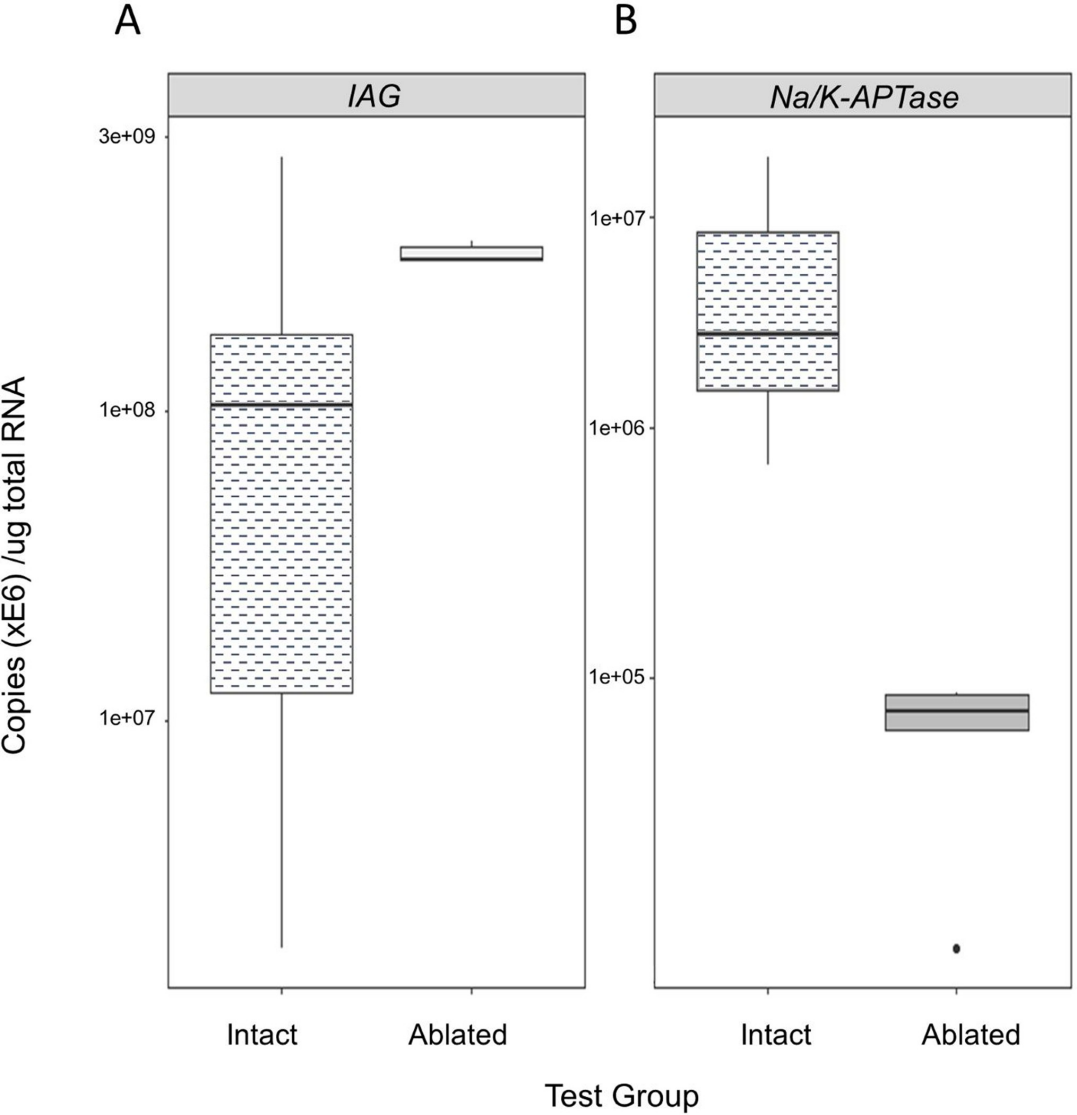
Boxplots of expression in intact and ablated *C*. *borealis* male AGs. Gene expression was measured by absolute qPCR assays and expressed in copies/µg total RNA. *CabIAG* transcripts measured in the androgenic glands of eyestalk-ablated (n = 5) and intact (n = 27) males were analyzed using a Welch two-sample t-test. Statistical significance was accepted at *P*<0.05. Expression was measured in copies/µg total RNA.

The expression of *CabIAG* reflected patterns is seen in the transcriptomic analysis. Specifically, *CabIAG* expression in AGs of ablated males (3.6 ± 1.0 x e8) is significantly greater than that of intact animals at 1.5 ± 0.4 x e8 copies/µg total RNA (t = 5.35, *P* = 1.46e-05, Fig 6). The *Na/K-ATPase* showed 5.3 ± 1.0 x e6 copies/µg total RNA in intact males, and 6.5 ± 1.4 x e4 copies/µg total RNA in ablated males. There was a significant difference in expression between *Na/K-ATPase* and *CabIAG* in ablated (t = 28.2, *P* = 9.47e-06) and intact males (t = 3.38, *P* = 2.69e-03, Fig 6). Further, *Na/K-ATPase* expression showed an opposite trend to *CabIAG* in ablated males, with *Na/K-ATPase* levels reducing instead of increasing after eyestalk-ablation.

**Fig 7:**
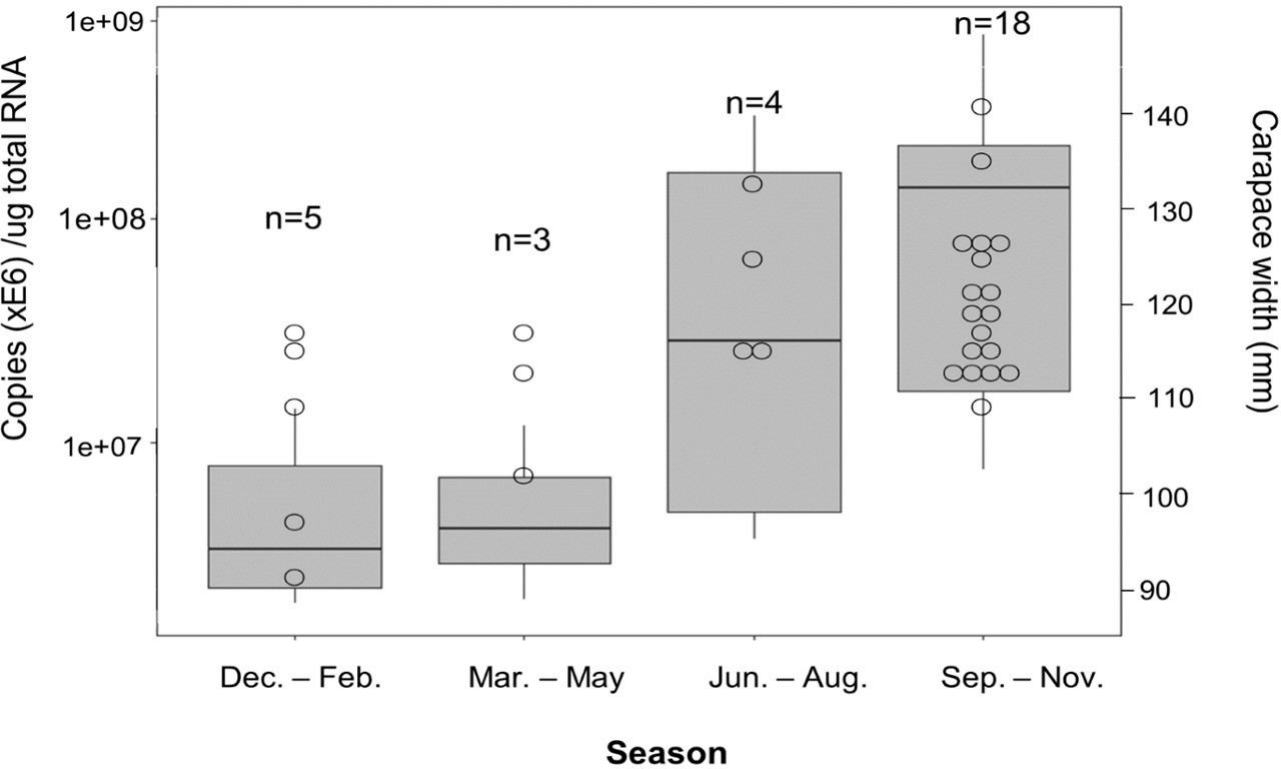
Boxplot of *CabIAG* expression by season in *C*. *borealis* males. Gene expression was measured by absolute qPCR assays and expressed in copies/µg total RNA. *CabIAG* transcripts were measured in the androgenic gland (AG) in the individual animals (n = 5 for Dec.-Feb.; n = 3 for Mar.-May; n = 4 for Jun.-Aug.; n-18 for Sep.-Nov.) obtained from different seasons and analyzed by a one-way ANOVA. Expression was measured in copies/µg total RNA. The size distribution of the animals is represented by the right Y-axis measured CW in mm, and each individual is represented by a circle with no fill. The statistical significance was accepted at *P*<0.05.

There was also a significant seasonal effect on *CabIAG* expression (F = 7.377, *P* = 9.85e-04, Fig 7). *CabIAG* expression was significantly higher during the fall months (September—November) at 1.8 ± 0.5 x e8 copies/µg total RNA (After removing 5 of the 23 for the ablation experiment, we were left with n = 18) than during winter months (December—February) at 6.1 ± 2.2 x e6 copies/µg total RNA (n = 5) (*P* = 0.0026, ANOVA and Tukey multiple comparison test) and spring months (March—May) at 6.2 ± 2.8 x e6 copies/µg total RNA (n = 3) (*P* = 0.019). *CabIAG* levels during the summer (June—August) were similar to that of fall at 1.2 ± 0.7 x e8 copies/µg total RNA (n = 4).

## Discussion

The current study reports *CabIAG* cDNA sequence isolation from the adult male AG of *C*. *borealis*, and a potential endocrine axis between the AG and MTXO-SG located in the eyestalk ganglia and serving as the source of eyestalk neuropeptide(s) regulating IAG levels.

### Transcriptomic analysis

The endocrine interaction between eyestalk ganglia and reproductive tissues is well-established in decapod crustaceans; more specifically, eyestalk ablation causes induced spawning in females and AG hypertrophy in males [10, 14, 19, 25]. *In C*. *borealis*, eyestalk-ablation (= removal of the MTXO-SG) changes the top 100 most highly expressed genes identified in the AG transcriptomes of eyestalk ablated and intact animals. The putative *CabIAG* showed conserved structural features and high amino acid identity in comparison to other IAGs. The *CabIAG* is specific to males, and exclusively expressed in the AG. A two-fold increase in expression of *CabIAG* is seen in the transcriptome. The *CabIAG* transcript levels also differed significantly by season, linking the importance of the harvest time of this species to sustainable fisheries management.

The top 100 most highly expressed genes contributed over 50% of TPM and were determined from the AG transcriptome of eyestalk ablated and intact animals. This is significant because relatively few transcripts are expressed and the AG transcription is largely devoted to a single transcript for IAG accounting for 7% and 25% of transcripts TPM before and after eyestalk ablation, respectively. [Any comparisons to other species could go here] Many of the remaining abundant transcripts are mitochondrial or involved in translation, presumably for the hypertrophied AG size and IAG transcripts. Interestingly, 10 out of 11 genes unique to the eyestalk-ablated AG transcriptome were not involved in translation or mitochondrial and classified with “other” for annotation purposes. These transcripts ranged from 1.5-fold and as high as 7-fold higher in expression when comparing eyestalk ablated to intact animals. The genes with high transcripts and high fold-changes: alpha 2-tubulin (1.7-fold increase), and an intracellular cholesterol transporter (3.8-fold increase), are not shown in the top 100 most differentially expressed. However, in the intact AG transcripts, over half the unique genes were related to protein synthesis, implying the consistent translation of mRNAs, including the *IAG*. It is important to note that the expression of protein synthesis genes unique to intact AGs was only slightly higher, less than a 1-fold change difference than ablated AG expression in all the genes.

Among the top 100 most abundant transcripts, three are represented in the top 100 most differentially expressed ones: trypsin-like enzyme, chymotrypsin, and PPOAE. Trypsin-like and chymotrypsin listed in the top 100 abundant transcripts are significantly downregulated in the ablated AG.

On the other hand, upregulated PPOAE in the ablated AG based TPM and edgeR differential expression suggests that eyestalk neuropeptides may control PPOAE expression. Furthermore, eyestalk neuropeptides influencing PPOAE may affect the PPO cascade involved in melanization, the essential component of the arthropod innate immune system [36]. However, it is not certain that upregulated PPOAE found in the AG could not be tissue-specific or exclusive to the AG. And, it remains to be studied if the eyestalk ablation causes PPOAE upregulation is in other tissues or is specific to the AG.

### Insulin-like adrenergic factor

The putative *CabIAG* showed conserved structural features and high amino acid identity in comparison to other IAGs. The structure of *CabIAG* ORF contained the signal peptide, B, C, and A chains, proteolytic motifs, and conserved cysteine residues found in other crustacean IAGs, including *C*. *sapidus*, *Scylla paramamosain*, *C*. *quinquedens*, *Fenneropenaeus chinensis*, *M*. *nipponense* and *Penaeus monodon* [10–12, 32, 37, 38]. For example, predicted proteolytic cleavage motifs of R-X-X-R were present at the predicted C-terminal end of both the B chain and C peptide, and are presumed to be involved in the ultimate removal of the C peptide [32, 37, 39] (S2 Fig). Further, all six cysteine residues of *CabIAG* are conserved in insulin-like peptides and other decapod IAGs (Fig 3A). These cysteines form two inter-disulfide bridges between chains, and one intra-chain disulfide formation [6, 40] and are predicted to do the same in decapods, possibly forming the functional heterodimer of A and B chains [8–10, 32].

Phylogenetic analysis of the putative IAG confirmed orthology with other decapod and crustacean IAG, despite relatively low overall sequence identity. For example, *CabIAG* was 60% identical to that of *C*. *quinquedens*, 49% to *S*. *paramamosain* and 48% to *C*. *sapidus*. The relatively low identity can be partially explained by the modular nature of insulin-like proteins, where the signal peptide and C peptide are cleaved from the preprohormone, leaving chains B and A for the mature peptide, and thus may be subject to less stringent selection. Further, chains A and B share more than motifs in common as there is high amino acid identity, especially around the conserved cysteines across species (Fig 4B). Specifically, adjacent to C_17_, C_59_ C_60_ and C_77_, a number of highly conserved aspartic and glutamic acids are located, similar to vertebrate insulin [41]. At the core of vertebrate insulin, a hydrophobic cluster of residues exists between the last two cysteines of the A chain. In *CabIAG* (Fig 4B), a highly conserved region of hydrophobic residues is also present between C_68_ and C_77_ of the A chain, possibly playing a role in the same folding structure as that of vertebrate insulin [41].

### IAG expression pattern

There are two different types of IAG expression patterns. *CabIAG* was expressed exclusively in the AG of male *C*. *borealis*, as seen in *C*. *quinquedens*, *M*. *rosenbergii*, and *Cherax quadricarnatus* [8, 9, 32] but different from other crab species. Clearly, it seems that there is another pattern of *IAG* expression in decapod crustaceans. *CabIAG* expression pattern differs from *C*. *sapidus*, *S*. *paramamosain*, and *F*. *chinensis*, where IAG transcripts are found and sequenced in other tissues (hepatopancreas and ovary) aside from the AG and in both sexes, and therefore may be associated with a different putative function [10–12].

Additionally, *Na/K-ATPase* as a reference gene had lower expression than *CabIAG*. *Na/K-ATPase* is a Na/K pump and is present in all cell types as the primary mechanism for controlling osmoregulation. In crustaceans, *Na/K-ATPase* is known to be stimulated in gills by salinity [42]. The animals used for the current experiment were maintained at 30 ppt, similar to that of the ecological habitats; hence, they were not expected to experience salinity stress. The downregulation of *Na/K-ATPase* transcripts in the ablated AG may be caused by the absence of a factor(s) present in the MTXO-SG that control ion-osmoregulation, as suggested in *C*. *sapidus* [43]. Further, *Na/K-ATPase* trends found in the eyestalk-ablated *C*. *borealis* need to be investigated to establish if the decrease we observed in this study is a typical pattern seen in the AG and other tissues and crustaceans.

Season affects *CabIAG* transcript level in the AG, which is possibly associated with the male reproductive activity. A clear pattern is seen with low expression during late winter through spring and the highest expression in fall and early winter, similar to the pattern of ovarian IAG transcripts reported in *C*. *sapidus* [22, 44], suggesting low reproductive activity. These results could be explained by mating and spawning seasons for *C*. *borealis*.

Somatic growth and reproduction of poikilothermic decapod crustaceans vary largely by season. All physiological activities regulating growth and reproduction (vitellogenesis, spawning, and mating) are cyclic, showing an annual or biannual cycle: high during warmer months and low during colder months. It would be interesting to study in the future if IAG levels in animals at a certain size change throughout the season concerning physiological activities, including mating and spermatogenesis.

The endocrine interaction between eyestalk ganglia and AG exists in *C*. *borealis*. The eyestalk ganglia or more specifically, the MTXO-SG within the eyestalk inhibits AG activity of *C*. *borealis*, as reported in other species such as *C*. *sapidus*, *P*. *pelagicus*, and *C*. *quadricarinatus* [10, 14, 45]. In these species, eyestalk ablation can cause AG hypertrophy and upregulation of IAG, possibly by increasing the number of IAG producing cells or cellular activities [15, 45]. However, the effect of bilateral eyestalk ablation on *IAG* levels can vary during the post ablation period and by species. Expression has ranged from a 2-fold increase in this study at 14 days post ablation, a 6-fold increase in *C*. *sapidus* at 7 days post ablation [10] and a nearly 8-fold increase in *M*. *nipponense* 14 days post ablation [46].

A specific neuropeptide responsible for regulating the AG activity also seems to be different between species. Gonad-inhibiting hormone (GIH) and molt-inhibiting hormone (MIH) in the eyestalk ganglia are the important regulating factors in *M*. *nipponense* [46], and crustacean female sex hormone (CSFH) is important in the Mud crab, *S*. *paramamosain* [47]. With the complete inventory of CHH neuropeptides, including CFSH in the eyestalk ganglia-sinus gland complex of *C*. *borealis* [48], it remains to be clarified whether CFSH or MIH regulate the AG and thus IAG expression in *C*. *borealis*.

## Conclusion

The AG of *C*. *borealis*, unlike Portunidae crab, is solely responsible for *IAG* expression, and its expression levels are influenced by season. Upregulation of *CabIAG* in eyestalk-ablated males, compared to that of intact animals, demonstrates the potential existence of an endocrine axis between AG and the MTXO-SG complex. It remains to be determined how C*abIAG* transcripts differ in juvenile stages and adults that could have the large size variation in the same season.

## Supporting information

S1 Table Top 100 most abundant Trinity gene sequence data for intact and ablated *C*. *borealis* males. An excel table of the top 100 most abundantly expressed Trinity genes from intact and ablated *C*. *borealis* males, and information for each gene including functional group, ablated/intact expression ratio, and rank in each dataset.
(PDF)

S1 Fig Volcano plot of differential expression. A volcano plot shows overall differential expression patterns where the X-axis reflects difference in expression between the eyestalk ablated males on the left and the non-ablated control males on the right. The Y-axis reflects the False Discovery Rate, a measure of differential expression. The top 100 most differentially expressed transcripts are marked with a solid square for the upregulated ones and a dotted square for the downregulated genes. Group numbers are presented using K-means clustering (K = 6) results. Upregulated are the groups 3 (circle), 4 (square), and 5 (remaining dots); Downregulated are the groups 1 (square), 2 (circle), and 6 (remaining dots). * = PPOAE; ** = trypsin-like enzyme; and *** = chymotrypsin.
(TIF)

S2 Fig Sequence logo for IAH and AGH ORFs. A sequence logo of MUSCLE 3.8.31 aligned decapod IAG and isopod AGH deduced amino acid sequences for the entire ORF region using https://weblogo.berkeley.edu/logo.cgi. The Y-axis describes the amount of information in bits*, and the X-axis shows the position in the alignment. The start of the signal peptide is signified by a black outlined star, the start of the B chain is marked by a blue outlined triangle, the C peptide is marked by a black outlined triangle, and A chain is marked by a red outlined triangle. The BLAST search identified a candidate IAG sequence from the top 100 ranked first in ablated and second in intact based on TPM values. The ablated/intact TPM ratio of 2.0 revealed that the IAG candidate gene was twice as abundant in ablated than intact males. The candidate IAG sequence was used to generate GSPs, allowing the full-length isolation of *CabIAG* via cloning from the AG, resulting in a cloned and transcriptomic *CabIAG* sequence sharing 98.7% identity.
(TIF)

S3 Fig Original gel image of the tissue distribution of *CabIAG* and *CabNa/K-ATPase* expression in *C*. *borealis* males and females. (PDF)

S1 File Top 100 differentially expressed sequences. The sequences are listed with trinity ids.
(XLSX)

S2 File Top 100 differential expression data with logFC and statistics. The values of logFC and statistics support.
(XLSX)

S3 File Top 100 differentially expressed sequences clustered using K-means (K = 6). The sequences belong to each group presented the sequence id using UniProt and BLASTx nr analysis.
(XLSX)

S1 Raw images (PDF)
