# Supplementary material for: Seasonal changes in the expression of insulin-like androgenic hormone (IAG) in the androgenic gland of the Jonah crab, Cancer borealis
Source: PLoS One. 2022 Feb 3;17(2):e0261206. doi: 10.1371/journal.pone.0261206 (PMC8812979; doi:10.1371/journal.pone.0261206)
Supplement: S1 Table — An excel table of the top 100 most abundantly expressed Trinity genes from intact and ablated C. borealis males, and information for each gene including functional group, ablated/intact expression ratio, and rank in each dataset. (PDF) [file pone.0261206.s001.pdf]

Column1	Column2	Column3	Column4
Sequence ID	Annotation	TPM Ratio (Ablated/Intact)	Intact Rank
TRINITY_DN0_c213_g1	Energy Metabolism	0.50408243	1
TRINITY_DN0_c311_g1	other	2.020327874	2
TRINITY_DN2_c261_g1	Energy Metabolism	0.45746445	3
TRINITY_DN0_c265_g1	Energy Metabolism	3.693976373	4
TRINITY_DN0_c265_g2	Other	3.433999018	5
TRINITY_DN2143_c0_g1	Energy Metabolism	0.013908532	6
TRINITY_DN393_c0_g2	Energy Metabolism	0.333181681	7
TRINITY_DN21_c62_g1	Energy Metabolism	0.616794302	8
TRINITY_DN33070_c16_g1	Other	0.088063844	9
TRINITY_DN169_c0_g1	Other	0.721422069	10
TRINITY_DN8203_c1_g1	Other	0.330216657	11
TRINITY_DN21_c63_g1	Energy Metabolism	0.853627447	12
TRINITY_DN1_c141_g1	Other	3.64444836	13
TRINITY_DN3_c0_g1	Other	3.620475684	14
TRINITY_DN45872_c9_g1	Protein Synthesis	0.840263812	15
TRINITY_DN13974_c2_g1	Other	0.33917026	16
TRINITY_DN45849_c24_g1	Other	1.147334611	17
TRINITY_DN79_c0_g1	Other	0.831305131	18
TRINITY_DN33093_c9_g1	Protein Synthesis	0.988426602	19
TRINITY_DN13139_c0_g1	Other	3.835767861	20
TRINITY_DN33203_c5_g1	Protein Synthesis	0.825917962	21
TRINITY_DN5_c0_g1	Protein Synthesis	0.864858123	22
TRINITY_DN4123_c0_g1	Protein Synthesis	0.896528754	23
TRINITY_DN65114_c3_g1	Protein Synthesis	0.80893143	24
TRINITY_DN58773_c11_g1	Protein Synthesis	0.880145487	25
TRINITY_DN45865_c17_g1	Protein Synthesis	1.053171622	26
TRINITY_DN52551_c10_g1	Protein Synthesis	0.960797385	27
TRINITY_DN13944_c0_g1	Other	0.777400919	28
TRINITY_DN11_c0_g1	Protein Synthesis	0.850508533	29
TRINITY_DN91_c2_g1	Other	0.804608815	30
TRINITY_DN45835_c6_g1	Energy Metabolism	0.678371086	31
TRINITY_DN16_c0_g1	Protein Synthesis	0.865092305	32
TRINITY_DN4_c4_g1	Protein Synthesis	0.793601989	33
TRINITY_DN52666_c5_g1	Protein Synthesis	0.844446782	34
TRINITY_DN33195_c3_g1	Protein Synthesis	0.81682844	35
TRINITY_DN91_c6_g1	Other	0.588167154	36
TRINITY_DN33080_c8_g1	Protein Synthesis	0.86750024	37
TRINITY_DN45824_c0_g1	transl	0.981699123	38
TRINITY_DN36_c0_g1	Protein Synthesis	0.943182372	39
TRINITY_DN2172_c0_g1	Protein Synthesis	0.926165497	40
TRINITY_DN52575_c7_g1	Protein Synthesis	0.997702519	41
TRINITY_DN64893_c3_g1	Protein Synthesis	0.793364697	42
TRINITY_DN2659_c0_g1	Protein Synthesis	0.942992133	43

TRINITY_DN33066_c5_g1	Protein Synthesis	0.951493913	44
TRINITY_DN58862_c2_g1	Protein Synthesis	0.930953953	45
TRINITY_DN1882_c0_g1	Other	0.822984052	46
TRINITY_DN3058_c23_g1	Other	0.873713665	47
TRINITY_DN734_c0_g1	Protein Synthesis	0.99877027	48
TRINITY_DN6_c4_g1	Protein Synthesis	1.23165641	49
TRINITY_DN33277_c2_g1	Protein Synthesis	0.808533597	50
TRINITY_DN19_c44_g1	transl	1.116332083	51
TRINITY_DN1316_c1_g1	Protein Synthesis	0.904181007	52
TRINITY_DN564_c10_g1	Other	1.067396146	53
TRINITY_DN33305_c3_g1	Protein Synthesis	0.966196986	54
TRINITY_DN14_c19_g1	Other	3.34455314	55
TRINITY_DN45968_c7_g1	Protein Synthesis	0.929102544	56
TRINITY_DN783_c3_g1	Protein Synthesis	1.005828375	57
TRINITY_DN270_c1_g1	Protein Synthesis	0.954014311	58
TRINITY_DN1135_c2_g1	Protein Synthesis	0.927496204	59
TRINITY_DN1567_c9_g1	Protein Synthesis	1.052109539	60
TRINITY_DN52660_c3_g1	Protein Synthesis	0.911526238	61
TRINITY_DN45851_c4_g1	Protein Synthesis	0.925067905	62
TRINITY_DN65025_c4_g1	Protein Synthesis	0.806477909	63
TRINITY_DN29_c0_g1	Protein Synthesis	1.019342805	64
TRINITY_DN20_c0_g2	Other	0.760160229	65
TRINITY_DN303_c0_g1	Protein Synthesis	0.984099277	66
TRINITY_DN39656_c2_g1	Protein Synthesis	0.963717899	67
TRINITY_DN288_c0_g1	Other	1.270131867	68
TRINITY_DN443_c1_g1	Protein Synthesis	0.881906853	69
TRINITY_DN743_c0_g1	Protein Synthesis	1.083049966	70
TRINITY_DN58615_c10_g1	Other	1.173069289	71
TRINITY_DN45895_c5_g1	Protein Synthesis	1.011666495	72
TRINITY_DN65249_c3_g1	Protein Synthesis	0.855904356	73
TRINITY_DN39601_c6_g1	Protein Synthesis	0.985247911	74
TRINITY_DN5847_c4_g1	Energy Metabolism	0.457318625	75
TRINITY_DN215_c0_g1	Other	1.329764188	76
TRINITY_DN39982_c3_g1	Protein Synthesis	0.935034849	77
TRINITY_DN1427_c0_g1	Other	0.800193248	78
TRINITY_DN1459_c3_g1	Protein Synthesis	0.884279621	79
TRINITY_DN960_c0_g1	Protein Synthesis	0.880759401	80
TRINITY_DN104_c0_g1	Protein Synthesis	0.988561994	81
TRINITY_DN33088_c9_g2	Protein Synthesis	0.993607773	82
TRINITY_DN45919_c3_g1	Protein Synthesis	0.844253373	83
TRINITY_DN576_c0_g1	Protein Synthesis	0.897177492	84
TRINITY_DN3875_c0_g1	Protein Synthesis	0.991233332	85
TRINITY_DN64868_c2_g1	Protein Synthesis	0.669729018	86
TRINITY_DN10_c0_g1	Protein Synthesis	1.028979775	87
TRINITY_DN52628_c2_g1	Protein Synthesis	1.080139886	88
TRINITY_DN52586_c2_g1	Protein Synthesis	0.860113407	89
TRINITY_DN397_c0_g1	Other	2.016838115	90

TRINITY_DN39520_c0_g1	Protein Synthesis	0.935273832	91
TRINITY_DN8_c3_g1	Other	4.413337382	92
TRINITY_DN39657_c6_g1	Other	1.382960182	93
TRINITY_DN52560_c0_g1	Protein Synthesis	0.92823866	94
TRINITY_DN52588_c2_g1	Protein Synthesis	0.937776597	95
TRINITY_DN2111_c0_g1	Protein Synthesis	0.921019718	96
TRINITY_DN45853_c4_g1	Other	0.912841784	97
TRINITY_DN27_c0_g1	Other	1.558229899	98
TRINITY_DN18_c1_g1	Protein Synthesis	0.7099888	99
TRINITY_DN28_c0_g1	Protein Synthesis	0.851616499	100
TRINITY_DN7897_c1_g1	Other	3.819281391	107
TRINITY_DN58643_c46_g1	Protein Synthesis	1.547278946	110
TRINITY_DN60_c0_g1	Other	1.690405805	129
TRINITY_DN7_c3_g1	Other	1.712935052	134
TRINITY_DN20699_c0_g1	Other	3.816405408	163
TRINITY_DN18853_c0_g1	Other	3.384902866	170
TRINITY_DN287_c0_g1	Other	2.103808495	171
TRINITY_DN12_c1_g1	Other	4.476001918	172
TRINITY_DN57_c1_g1	Other	4.203460453	180
TRINITY_DN195_c0_g2	Other	5.131664456	256
TRINITY_DN1288_c0_g1	Other	7.519163293	332

Column5	Column6
Ablated Rank	Sequence
2	TTTAGTTAACATGAAGCTACAGCTTTATAGGGTCTTATCGTCCCCTTGAATGATTTAAGCCTTTT
1	CAGTGAGTGTTGCGCATAGAAACACACACAGCAGAGGGTGTCAGAGTGGCAAGGCACTGTGC
5	TTTTTTTTTTTTTTTTTTTTTTTTTTTTTTTTTTTTTTTTTTTTTTTTATTTCAACAAATAAAAACTTTA
3	AGCAGCCTTTTTTCGCAGACAAGACGCCGTTGTCAGGAAAACCCAACACGTTGACGGTTGAAT
4	AGACGTGTGCTCTTCCGATCCGTGGCTGTTGGCGACACCTTGTTACTGTTCCACACAATACAAG
386	CTACGTCATAGACAGCTCTTCTTTTGTCCATCCCTTCATACGAGTTCCTAATTTAGAAACTAATG
32	TTTAAGACTAATTTTCTTTAGGGTTAACAGCGTTATTTTTTTTTGAGAGTTCATATCGAAAAAAA
14	CCTCCCAGCTTCAATTGAGTGGGCTCATTTTTACCCCCCTTCAGATCATTCTTATATAGAAATTC
170	CCCCCTTTTCAAGCAGAAGACGGCATACGAGATCGCGGTTCGTGACTGGAGTTCAGACGTGTG
13	TTTTTTTTTTTTTTTAGGTGGTGGTCCCTTCTTTCTTTGTTTTTTTTTTTTTTTTTTTGTTTTTTTTTT
55	ACTGATATTTGATTGATATTTATTTGATCCTTTTAGATAAAGATTTTATACTATGTTACTTTAGG
11	CATTTTTTCCATACACGTCGTTCTTACTATATCACTAATATTTCATAATTTATAATTTAATTAAATT
6	TGGGAGTGAAACATGGATGATGCTTAACAGACACAAACTTGAGTCTATAAGTGTAGAAAGGA
7	TTACCTTTACCTCTACCTTTACCTGGTCGCTTGCATCCTTTATTTTTTTTGCATTTTCTACCTCGGC
16	TTTTTTTTTTTTTTTGATCAGCCACCTTTATTCATCTTAGTCTTCAGATTTTGTGGTTCTGGTATAT
94	TCTTACTATTTTATCTTTCCTTTTTCTCAAGAGATAGAAACCGACCTGGCACACGCCGGTCTGAA
15	ATCCTTCGCTATTTTCACCTCTTCTTTTGGTTAATTCCAAGTACCTATACTTCTTTGGGAAAAGCT
19	GGCTAAAATCCAGGACAAGGAAGGCATTCCACCCGACCAACAGAGGCTGATCTTCGCTGGTA
17	CCCACAATGCCTTGCGCGGCCACCAAGCTCCTCTACTGTGGTGGCCGGGACATAGGTCTCCAC
8	CTTTACCTTTACCTCTACCTTTAACTGGTCGCTTTCATCCTTTATTTTTTTTTCATTTTATAACTCGG
23	CCGATCTCCAGAAAACACCATGACCAAGGGTACATCGTCATTTGGAAAGCGCCACAACAAGAC
21	TTTTTTTTTTTTTTTGCAAGATGATTTTTTTTTTTTTACTTTTAGAGTGACACATTAGAACTGGATC
22	AGGAGGGCCAGCGCGAGCCTCTACCTTCCTCACTCTCCCTTCCACCAGCCTGCTTCTCCCCGCT
28	TTCCTTGATTTCCTTTTTCCTTCCACAGCAGCATCTCGAAAGCCAGGTGACTGCTGCCACCAACA
25	TGTTGGCAACACTGAACATCCCTTTCCCTTCTCCCATGTGTTGACGAGTGTGGAAGAACGTTGA
18	TATTAATATTTATAATATTTTATTTTTTTTTTTTTTTATTTACTTTTATTTTTTTTTTTTTTTTTTT
20	TTTTTTTTTTTTTTTTTTTTTTTTTTTTTTTTTTTTTTTTTTTTTTTTTTTTTTTTTTTTGCAGCCTATAC
37	TTTTTTTTTTTTAATTCTAAAAGGGTAAATATTTAGTTTTAAAAAAATAATTAGGTTTTATAAAG
30	TCTGACATTCTGTCTAACCACCTCTTTTTTGCTTTTTTAAATTTTTTATTTATTTACCGGGCTGCG
35	TGCTGCCCTTTAGGAAGACCTGATACCACGCTGCCATTTAGGAAGACCAGATGCCACGCTGCC
53	AACCTTTTAGACTAACCTTTAATTTTAAAAATAAACTACCCAACCCCCATAAATATTTAAACAGG
29	CCGGTCTTTCCATTGAGGCGGTCCAGGAGTTGGTGGTGTGGTGACCGCAAACATGCAGATCTT
44	GTGAAAAGAACCATCGCCTTACTCCTTCACCCCGCCGCCTGCACATCGTCAACATGGGCTTCGA
40	CGATCTGCGCAGAACCGTCCACTCCTGCCTTCCATTCCTTGACAACAACACACGAAGATGCAGA
50	TTTTTTTTTTTTTGTATCTTAGCTTTTTACTGCTCACACTCTAGAGGGGTACATCATAATCCTCAC
89	CCATGCCCATCCTCTTCACGCCATGATGACCGACAGAGACCATGCCCATCCTCTTCACGCTACG
46	TTTTTTTTTTTTTACTTCCTAACTTTATTTAGAAGAAGAATCCAAGAATCTTTCCTCCCAGGTGTT
34	TCCAGTTTCTTTATAATAATGCAATGTGATCCAATTGTAATTGTAATGTGGGGGGGGAAAATGT
38	TTTTTTTTTTTTTTTTTTTTTTTTTTTAAACCCATGACAACTTTATTAATTTTCCTTAACTGTGTATT
43	CTGGTCTTCTTATTGTCTTTTTCCACGATCGGCCGCTAACATGTCCACACACAGGAAGAAGACC
36	ACCACCCTTCCCCCACCCCCCCCCCTTCCCTTCTGTGTTATAATTAAACCTTTTTTTTTTTTTTTTT
66	TTTTTTTTTTTTTTTCTTGAGCTGAGAGGACAGCGCCAAACATGCCTCTTGCACGTGATTTACTA
45	CGATCTCATCCGCTAACGTAACGTCCTTCCCAAGACATCACCGCCAAGATGTCGATCATTTTGC

49 TTTTTTTTTTTTTTTTTTTTTTTTTTTTTAAATTTATGCTATTCTTTATTAGAACTTTTGGAACTGCTT
52 TTTTTTTTTTTTTATATAACTCCCACTTTAATCATCAGAGCCTTCATCTACATTCTCAGTTCCCAGT
70 GTCCTGCCATCCTCCAACTGCTTTCCAGCGAAGATCAGCCTCTGTTGGTCAGGTGGAATGCCTTC
61 CCAATTTTTAAAGTTTACTTCAGTCCTTGATACAACTTTTCCTCTCAGCTGGCAGTCTTTATTACAT
48 CTTTTTTTTTTTTAAATCTATCTTTATTTGACTCAGCGACGGCCACCATGTCCACCGTGTCCCGGC
27 CGGCGCATTCTCTTCTCCAGATGCTCGTCAGAAGAGCAAGTACCACGAGCTGTGTTCATGACCT
77 TAAAAATACAATATACATATAAGTTCATGGGTGGCAGATAATAATTATATATTTTCTCATTGTTG
39 CCGATCTTTTTTTTTTTAAATTGCAATGTTTTTATTTTTATATATTTCTCTTCCCTGGAGTATACATT
65 CGCTCCTTACATCCCTTACCAAAACACGCGCCATGGGGAAGGAGACGGAAACCACCTGTACCAT
47 CGGTGGTCGGTCCTTCCGTGCCGCCGCCGCACAGGTCCTGCCGCTCCTTCCTGCTGCCCACCAC
59 TTTTTTTTTTTTTTCTCAACGTATTTTATTTATGCAACTAGGGCAGAGGCTGTAGCTGATGCATAT
9 AGGTAAAGGTAAAGGTAGAGGTAAAGGTAAAGGTAGAGGTAAAGGTAAAGGTAGAGGTAAA
63 TCCGATCTTTTTTTTTTTTTTTTTTTTTTTGCAGTGATGACATTTATTAACTCTCAACACCATAGGA
58 TAATTTTTTTTTTTTTTTTTTTTTTTTTTTTATTAATCTATGGCTTTATTGGTTTATTTAGTCTTCTCAG
60 CGATCTTTTTTTTTTTTTCACAATCGCATAGTTTTATTTAGACCTTAGTCTGTACAGGTGGTTCATA
71 GTTTTTTTTTTTTTTTTTTCTTTCACAAGTTTACTCTTATAGTTTACAGATTTTACTTCTTGCCCGTG
57 TTTTTTTTTTTTTTTTAACATCTCATCTTCTTTATTGAGGTGAGCTGAGGTGCGGTGTGGTGTGGT
75 TTTTTTTTTTTTAACTTTCTTTTTTATTGTAGAATATTTTACTTGTGTCCTGCAGCAGCAGCAGCGG
73 TTCCCTTCATCACACCGCCACCATGTCGAAGAGAGGACGTGGAGCCTCGTCTGGAGGGAAATTT
90 CACCCACCCCCAACCGCCCAACCGCTTCGCGGTGTGGGTTTGGTGTTTTGTCGTTTTGTGTTCCT
67 TGCCTGCTCTTTCTCTTCGCCTCCACACACGCTCCACGATGCCGAGTGTCAGTGTGAAGGACGTG
96 ATCCTATGGTTCGTTAACATCCTGGATTGTATGGTTCTCCTGGATCCTATGGTTGGTCTTGACTG
72 CTGATGACGATGAGCCTCCACGGTCCTTCCTCCCCGCCAAGCACACACCAGGTGGTGACGGCAG
76 CTTTTTCTTTTTTTTTTTTTTTTTTTTACTACTAATCCAGTTTATTTAGAAGCGAAGTTTCTGGAAAA
41 TCTCTCTCTCTCTCTCTCTCTCTTTCTCTCTCTCTCTCTCTCTCTGTCTTCCTCAGCGATAAGAAA
85 CCCCCCACCCCCCCCCCCCCCCCCCCCCCCCACCCCCCATTACCGCCACAATTTCCCTACCCGA
62 TTTTTTTTTTTTTTTAACCAGTCATGTACTTTATTTATCCATCTCGACAACCAAATATACGAGTGTT
56 TCAGGTATTTTCTTGTCTTGCTATTATTAATTTCATGCCAAAACCACACAAACAAACACACACACA
74 TTTTTTTTTTTTTTGTCAACTTTTTCTTTACTTAGGGGTACACAAGTTACTCGGCATCTTCCTTGCT
93 CCGATCTCTCAGCGTGTGCGCATCACGATGTTCTCCTTTACAGTTCTCTATCCCGTGTGAGACGT
79 TGTCTTGCTTCCCCGCGTGGCTGATCCAGAGAACACTGGCCGGCCGTACGCGCCCCTAAAACAT
145 TAAAAACATAATATAGGGATAACAGCGTTATTTTTTTTTAGAGTTCATATCGAAAAAAAAGAATG
42 CCCCCCCCCCCCCCCCCCCCCCCTTTTCAAGCAGAAGACGGCATACGAGATTATAACCTGTGACT
83 CTCATTCCTTCTACAAGTCCACAGAGGGCCATTGCCGCACCGTAAGCCATGGCTCCACGTAAAG
98 GCTCGCTCGGTCGGTCAGCCCGACTGGGCCCGGGAACTGGAGCCAGTTGAGCATAGGATATCT
91 TTTTTTTTTTTTTTTTTCTTCTAAATGATTTGTATTATAAATCACTCATCTCCATCATCATCATCATCT
92 ATCGGTAAGACTCAGACCTTCTCTCCACGCCGCCTCAGTGTCAACATGGACAAGCCTGTGAAAC
81 GATCTGTCCATGTCAGCATATCATTTATTTACTCCTCTTGCTCTTGATCTTCCTAAGATAGAACTC
82 TTTTTTTTTTTTTTTTTTTTATTATTTTTTTTTTTTTTTTTTTTTTTTTTTTTTTTTTTTTTTTTTTTTAAA
97 CCGATCTTTTTTTTTTTTTTTACCAATGCATCTTTATTTTCTTTAAAAACTTTATTCCACACGCATCT
95 GCCTTTATCATACACTTCCTCCTCGTGGGTAAAGTCTGTCCTCCGGTTCAGCGCTGATTTTTCGTA
87 TTTTTTTTTCAGCCGTTCGCCTTCGAGCTGGGAGGATGACATCCAAACGTCGTAACAATGGTCGT
123 TTTTTTTTTTTTTACTGGAGCATTATATTTATTTAGTAGTTCTTGGGAAGAATCAAATCTTTCTTGG
88 TTTTTTTTTTTTTTTTTTTTTTTCATCCTTCAATCCTTTATTCATATCCTTCAGTACAAACAGGATAT
86 CCGATCTTTTTTTTTTTTTTAACAGTTGTAGGTTTTATTTCACTACAACACCAACACCACCACCATG
109 TTTTTTTTTTTTTTTCATCACAGCGAGTTTATTCATCGCCTTCACACGGTCACAGTCTAATCGTCTT
24 AATTTAATAACATTACCTAATTTTAGTTTATTTTATATTATGTAACTTTCAATTTTTTTTTTATTTTT

100 TCTTTTTTTTTTTTTAGTTTTTATTCGTGATCGACTTCATAGAGAACGTCTGACCACATTAGTCAA
10 AGATGCTTGGAGAGAAAATACTTTTACTACCTTCTTTCAATAACAGATACACATGCAGGATCTA
64 CAAGTGAGGTGAGGCTAACTAGCCTTTAAACATTTACGTATTGTAGCTATATACAAGGCTTTTA
105 GTGCTCTTCCGATCTCCTAACACCAAGGTGAGGCTGGCGTACACATGGTTGATATTTAACACGA
106 TGTAGTGTAATTATTTTCCAGTATTGTTGTGGTGTGAGAGCCATCTGACGGCGCCTCTTTATAC
110 CCGATCTTTTTTTTTTTCACATTGATCCAAAGTCATGTTCCTTTATTAAGCTCTCAAGAACACATT
113 TTTTTTTTTTTTTTGATCACACAACAATTTTAATGGAATTTAAAAGAACAACAATAATTCAAATG
54 CGCCTATTTGACTCTCGTCAAAGAGGCGAACCTCCCCAAGGCGGTCAATGCCACGCACGCAGT
134 CGTAGTCTTCCTGGGGTGGAGACACGGGCAGCCGAGAAGTATCTTTCCTCTTGGAAATATCCT
119 CGATCTGGACAACACGGTGGAGACAACATGGGTAAGCCACGAGGTATCCGCACTGCCCGTAA
11 CTACCTTTACCTTTACCTTTACCACTACCTTTACCTTTACCTCTACCTCTACCATTACCTTTACCTC
68 TTTTTTTTTTTTTTTTTTGTTTTGTTTTTTTTATTTTTTTTTTTTTTTGATTTCGCCATCGGGTTGCGT
79 CTCAGCTGTTTCCTAGTGCTGTGCATGCTGACGTACTCCTTCCTGCTTTTAAGAAGGAAGGAAG
83 AGAGAGAGAGAGAGAGAGAGAGAGAAAAAAAAAAAAAAGAGAGGGAGATACCATATATAAAAA
30 TCAGGTCTTCTGGTTACGGAGTGAGTACCCTGTGATATGGTCGCTGGTGGACAGTAAGACAGA
50 CTCTACCTTTACCTTTACCTTTAACTCTAACTTTACCTGTACCTATAGCTTTACCTTTTCCTTTAAC
98 CTAACTAACTAACTAACTAACTAACTAGAATGGACAAACAAAGGAAAAAGAGAATAAGAAGA
25 CCGATCTCTTCCTCGAATTTCTTAATTTCGTAATATTGATGATGATAATTATTATTAATAGTTTGC
32 CTGCTGTCGTGGTGGTTGTCTTATACTTCCCGCCTGTGTGTTCCAATTGCCTAGCAGAACTTTTA
77 TTTTTTTTTTTTTTTTTTTGGAGCAATACAGTTCAATTTTATTATATGCATTTACATGTATATCAAT
67 CACACACACACACACACACACACCATAGGAAGAGGATAAAAGCAAACAGGGGGAGAGGAGG

CACTTAAAAGTTAAATTCTATTTCTACGTCAGAGACAGCTCTTCTTTTGTCCAACCCTTCATACGAGTTCCTAATTAA
CAACCAACATAGAGGGGGTGGGGTGTGGGGGAGACGAGCAGAGCAGTCAGCACCATGAAACAATTGGTGGTG
AGCTATTAAATCCTTTCGTACTATAATAACTTTCCTTTTTCTCAAGAGATAGAAACCGACCTGGCTCACGCCGGTCT
AGAAGCCAAGGTTGACAACGCCACCATCACCGTCCATAAACTCGGTATTGGGAAAAACGCATAGACTTTCTCCGCT
TGGAAGTGTGGTTAACATTACACAGGACAGGGATTCCACTATCTCCAAAAGTTAGTGTTGACTTGTTTTTAATGCA
ATTATGCTACCTTTGCACGGTCAGAATACCGCGGCTCTTTAACTTCTTGTCAGTGAGCATGATAGACTATTTATTAT
AAGTTTTCGACCTCGATGTTGAATTAAAATTTTCTTTATAGTGCAGCTGATATAAAAGAGGGTCTGTTCGACATTTA
CTATAATTACTAACTTCTAAAATGGCAGAAAGATGTATAGGATTTAAGCTCCTACTAGGAAGAAACTCTTCTTTTAG
CTCTTCCGATCTCTGGAATCCACACCACCATCATGCGCGTTCTTCTCCTCGTGGTGAGCTTGGCGGCCATAGGGCA
TTTTTTTTTTTTTTAGGTGGTGATAATCTTTTAATGAGAGCCAGTTACAACAAAATTATTTAGTGATCCAACACCAAG
TTTACAGAGTTATTTTTTTTGAGAGTTCATATCGAAAAAAATAGTTTGCGACCTCGATTTTGAATTAAAATTATCTT
TAATAATTTTTTAATAACTTTTTTTTTTTTTTTTTTTTTTTTTTTTTAGCCGCCCCACCAGTAAATAAAAATATAAAGGA
TAACTCGAGGGAACATAATCAAGACCTGTAGATCTGTATGTGGATGAAGATGTGTTGGGAAGCATCTGGATGAA
CTTGTCGACCGAGTTGTTCGAGTGTAGAGGACTGGTTGACTAGGCTGGTTTGAGAGAGTGGTTTGGGTGATAAG
ACTACCTGTGCATGATGCTTAACCACCTGTCGGATGAGTCCTTTGGTCTGGAGTTCATCAAGTGCCCGGCGTGCCA
CTAAAATCATTTAAAAATTTAAAGGTCGAAAAGACCCTCTTATATAGCAGCTGCAAAATAAAGATAATTTTAATTC
GTACTTTTTTGTGTCGTTCAAATAAATTTCTTTTCACAGTTTTTCCTGTGTCCCCTCAAATATCCAGCCTCATTTTCTT
GCAGTTGGAGGACGGACGAACATTGTCTGACTACAACATTCAAAAGGAATCCACACTCCACCTTGTTCTCAGGCT
ATGAAGCTCAACAAGCTTAAGACTTCCTCCCGGCGCAAGAACAGGGAACGGTATTTCAATGCCCCGTCTCACATC
CCGTGTCGACCGAGTTGTTAGAGTGTAGAGGACTGGTTGACAAGGCTGGTTTGAGAGAGTGGTTTTGGTGATAA
ACACACGCTGTGTCGGCGTTGTGGCCGCTCTTCCTATCACATCCAGAAGAAGGTGTGCGCACAGTGTGGGTACCC
TCATTCCCTTCCTCTTCTTGTCACCACCCAGTTCAAAGTGTTTACATCTCTTGAGAGGAATCTGGGTCTTGATTCTG
GGGTTAAAGGTGATTACTGATCGCCGAGTTGCAGGGTCATCCAGCCTTCTATAGCCTTCACTATGGCTTCCAACG
CCACCACCACCATGCCGAAGCAAATTATGGAAATCAAGGACTTTTTGTTGACGGCGAGGAGGAAGGACGCTAAA
CGATCGAGGACGTCCAAATAGTTCAGCATGTCTGATGTGGAAGGAGACGTTAGCGTTCCGGTGGCTGGGGGCCC
TTTTTATTTTTTTTTTTTCTTATTTTTTTTTATTCCAGTTTCTTGATCTTAGTTTACTTTTTGTGCAAGCCTGCGGTCGCG
AAACATCCACTTTATTCGCTCACAACAGGCTACAAGTTTTTTTTCTTCCCTCACTTTCATCCACTTGACATCCGCATC
GGTTTAAAAAGTGTAAGTATTGTTATAGAAATTAGAGTTAATTAGCTTAAAGGGGAGGTTAGATTACAATTTTGA
TTTGGGGTTTCTTTTTTAATTTCTTGTTTTATTTTGGTTTTTGTCCTGGTAATATTTTTTTTTTTTTTTTTTTTTGACTAT
TTTAGGAAGACCAGATGCCACGCCGTCCTTTAGGAAGACCGGATGGCACGCCGCCCTTTAGGAAGACTGGATGG
GTTCTTCTTTTCATTTTCTTACAACTAGCCTTTATTAAAAATAAAATATTTAAAAAACACCTTCTTTAAAATTAATACA
TGTGAAAACACTCACAGGGAAGACCATCACCCTTGAGGTTGAGCCTTCTGATACTATCGAAAATGTGAAGGCCAA
CACTATCTGGAACTCACACCCAAAGAAATACGGCCCAGGCTCCCGTAGGTGCCGGGCTTGCTCTAACCGTCACGG
TCTTCGTGAAGACCCTAACGGGGAAGACCATCACCCTTGAGGTGGAACCTTCTGATACTATTGAAAATGTGAAGG
TCTCCTTCCCATGGCATAAGCAGGGAGTGATCTGGCAAACTTAGCGCGTAGAGCCCCAGAGTTCCCGTGGATGCG
TGACCGATGAAGACCATGCCCATCCTCTTCACGCCATGATGACCGACAGAGACCATGCCCATCCTCTTCACGCTA
TCCTCTTGGCTTCCTCGTGGTCCATGATTCCGCCAGATGTGGTCAGTACAATGTACCCGAACTGCCTAGAGGGGAG
GCCAGGCAACAAAGACCATATGGCACAAGGTTAAGGAAAGATAAGGATTATGTTAAGTCTCACGGAAAAGAAG
GAAACAGGCACGTGTCCGCTTTCCTACACGCTGGTGGCCTTGGCTATTAGCACATCACGCTTCTTGGCTCGGGCG
GGAAGCTGCGAGGCCATGTGAGCCATGGACACGGCCGCGTTGGCAAGCACCGCAAGCACCCCGGTGGTCGTGG
TTTTTTTTTTTTTTTTTTTTTTATTGCAATGAACAAATTATTCCCTGGCAGCCATAAGCTTTTGGCGTGCAAGTTTCTTC
ATCCGTCACCGGTGGAGGAGAAGAGGAAGTGCAAGCTGAAGAGGCTTGTGCAACACCCTAACTCCTACTTCATG
GGAGAAGTTCCAGCACATCCTGCGTTTGATGAACACCAACATCGATGGGCGGCGCAAGGTTATGTTTGCCATGA

TCTTGGTGCCTGGCGCTTTGCTGACCTTCAGTACATTAAAGCGAACAGTTTTGGATAATGGGCGGCACTCACCCAC
CCCTTAAATGAAGCAACAGGGACAAAGGTTACTAAGGTATAGAATGGGTGAGTAGAGTCTTCATCCTCGTTGCGG
CCTTGTCCTGGATCTTGGCTTTGACGTTTTCAATTGTGTCTGAAGGCTCCACTTCTAGGGTGATGGTCTTACCAGTA
TCTTTTTCACCCAATTTTTAAAGTTTACTTCAGTCCTTGATACAACTTTTCCTCTCAGCTGGCAGTCTTTATTACATCT
CTTCCAGCGCGGGCCATCTTGGGAGCCTTTTGGGTGGTCTTCTTGGACTTAGATGGGGCAGGTGCATTGGCCTTC
GTGCCAACCGGCGTGGGGTATGCGAGGTCAGGCTGGGTCAGTCACACAATCTACGAGCCATCTTCAAATCGCCA
ACTCTGGACATAGGTTGGCAGCCCTGCGTCCTCCCTTCCCAGACAGCACCAGTCGAAACCTCCCGGCAAAATGGT
TTGATTTCCACAATTAGACAACCCATACGGATTAGGTTTTCATCAATAATGAGATACATTACATACGTAACAATACC
TCAGGACCAGGAAGTTCCTCACCAACCGCCTCCTTAACAGGAAGCAAATGATTGTTGATGTCCTGCACCCCAACCG
TAAGTGCAGAACATCCATCCATCATGAAGGTCTTCAAGGATCTCGTCTCAGGTGATGAGATGTTCACTGACACCTA
TTTCCAGGTTGGGGGCAGCACATTACGAGTCTTGTAGTATCTTGCTAGGCGATGGATACGTGACTCTACCAAAATG
GGTAAAGGTAGAGGTAAAGGTAAAGGTAGAGGTAAAGGTAAAGGTAGAGGTAAAGGTAAAGGTAGAGGTAGA
GACCATCACCTTGGTGTACGGCTCAACCCTCTTGGCAACCTTACCAAGAGCCTGTCGCCTCAATTTCTTATCAGTAA
ATTTCTCAGTCTTCTCGGTCTTCTCAGACTTCTCGGTCTTCTCAGACTTCTCAGATTTCTCAGATTTCTCAGACTTCT
AGTTGTCTGGGCGATCCACCTTTGACCCGACGTCCTCCCCTGTGGCGGTGGTGGACTTGCCACTCTCACCGTGCAT
ACAGCAGCGTCCTCGGCAGCTTCCTTCGCCTTCTTGGCCCGCATGCCCCACGTGCGTTCCACGGCCCGTTCCCGGC
TGGTGTGGGGTGAAGTACTCCTTGCCTCCACCTCCACTCCACCTCCTCCTCCACCTTAGATTTGACCCGCCAGTTCC
CGGCGGCAGCAGCAGCAGCCGCAGCCTTCCTTTGCTGACCCATCACACGCTGCATTTCCTCACGCTTCCTCTTGG
TCGAATTTCCCTCGGCCTGCCTGTAGGTGCGGTTATGAGCTGTGCCGACAACACGGGCGCCAAGAACCTGTACAT
TTTTTTTTTTTTTTTTTTTTTTTTTTTTTTTTTTTTTTTTTTTTTTTTATCTTTCAATATTCTTTATTTACGCAGCTTCCTT
CAACCAGCAGGCCTTTACTGAGGCTTTTGCCGAATTCCTAAAGAAATCCGGCAAGGTCAAGGTACCAGACTGGGC
GTTCATCTTGATCCTATGGTTCTGTATCCTATGGTTCGTTAACATCCTGGATTGTATGGTTCTCCTGGATCCTATGG
CAATGTTGATGCCCAAAGAAAATCGTGTTGCCATCTATGAACACCTCTTTAAAGAGGGTGTGATGGTGGCCGAGC
GCCAGCGGTTCTTCCCAGACTTGTACCTTTCTTCTAGTTTGGCCTTGACCATCCTGCGGGCCTTCTTCCTGCGGGCA
GACTGAACCTCACCTTCTGATCCTCGTCCTCGTCCTGTTGGTTCTCCTCGTCCTCCATCACCCTCGTCACGATGCCT
CGCTCTCCGATCTTTTTTTTTTTTTTTTTTTTTTTTTAACGGTAGGTATCCTTTACTCTGGTTGTACAACGTGTCCCTTC
TTTATGCAATGAACTCGTAGGGCAGTGGCGTCAAGAACTCGGGTTTCTTCTTGGTGATGTTGACCTTGTGTGCGAT
ATACACGCACCCGCACACACACACACACACACGAGGGAATCACACCACCAGTCCTTAGTGTGCACGCAGTATATTG
CTTCATTGCTTCAGCTTTCCTGACAGCAACACGATCCTGCCTTCGCTTGCGAGCTTCCTTCGCCTTGTTTCTGCGTG
GCTGGCTCCTGAAGTCTGAGGGTGATCCACATAATCCGACGAAATGGGTCGTGTGCGTACAAAAACTGTGAAGA
TCAAAATGGGGTATGTCAAAGTAGTCAAGAACAAGGCTTACTTCAAGCGTTTCCAAGTGAAGTTCAAGAGGCGCC
GCGACCTCGATGTTGAATTAAAAATATCTTTATAGTGCAGCAGCAATAAAAGAGGGTCTGTTCGACCATTAAATTT
TGGAGTTCAGACGTGGCTCTTCCGATCTGGCTGACAACACGCTGTGACGGAACCCAGGTGTTTTGAATTACTGTCT
GAAAGACCCAGAAGGAGGAGGTGCAGGTCCAGCTTGGCCCGCAGGTGCTGGATGGAGAGAAGGTGTTTGCTGT
TTTGAGTTAACCTTGGAACCCACCCACCCTAATCACCACCAACATGAGCACAGACGCTGCCCTCAAGGACTTGCCC
TCTTCATTGTTGATCTGGAAGTATCGCAACTCGTACATGTCTTTGGCAGTGGGAGGAGCCACCACTCTCAGCCAGT
TCGCCCGTGTGACCAAGGTGCTGGGCCGGACTGGGTCTCAAGGCCAATGTACCCAGGTGCGCGTTGAGTTCCTG
CAGCTCCTTGCCCTCCAGGATGTAGCCATCAGCGCGGCCACTCTGGCCAGGGCGGGAGGAGATACAGGCTAGGA
AATTTTTTTTTTTTTTTTTTTTTTTTTTTTTTTTTTTTTTTTTTTTTTTTTTTTTTTTTTTTTAATATAACCCATTTATTAGAG
TTCAGCCTATTCCGTGGGCATGGTGCGAATGATGTCAGAGTCACCCGGATCAGTGATGGCCAATGAGCACACCCT
AATTAACTCGACCGGGGAAGCAAGATGGCGTCTATGTATGATAAAGGCGACATGAAGAGTGGGATGATGGATGA
TTCCAAGCATGGACGCGGCCACACCAAGCCGGTCCGCTGCACCAACTGTGGCCGCTGTGTGCCTAAGGACAAGG
GCCAGACGAGCAAGACAGTCATCAGACTCACCCATCTTCCTAATATCTCCACAGATGGCATAAGTCTTGTACTGAC
CCTTTTAGGCAGCGTGTCTGGGATCCTTCTTGGTACCTCGGATCAGACCAGTCCTACGAGCAGCAATCAGGCCGA
CACAGCCTCCTTAGAAGCACTTAGGGATGGGACGGCCGTAGGTAATGCGATCTCTCTTCATGTTCTTCTTCTTGTG
TCACCGCTGCCATCCTCGGTCTTGCTTCCTCCCTTGCGCATGTTCTTCCTCTTAACGCGGCCGTAGCGTCCGCCGCC
TTTTTTTTTTTTTTGGAATATAACTCTGTAATTCCAAAGTCATACATATAATCCATATAGTTTATAATTCGTTACAA

AGAGACCCATGCCGAAGTCTTCATCGGACTCCTCTTCCTCCTCCTCAACCTTGGGAGCCTCCTTGGGGGCTTCTGCA
ACCTAACCTTACCTAACCTACGCAAGCAAGATCGTTAATATAGATGCTTGGAGAGAAAATACTTTTACTACCTTCTTT
TTTCCCGGCGCACGAAATTTAACATTTAAACAGAGAACATTAAATTAATCAATGTTTAAAATGTATGTATTGATGGT
ATTTTACGCTGTTAATACTCCGTTACCTCAGTTGTTGTTCACTGTGATGACTGATAAGGTTGTGTTGCCTCCTCAGAT
CTTCTTCCTGCCCGATCTGCAGTGTGTTTCGGGCCAAGATGGTGCAGACCAAGACAGTGCGGCGATCTGTGAAGC
ACAGACCACAACATTCTCTCTTTACTTCTTGTACCCGCAGCTTGACCTCCTGCCTCGGGCCCTCTCAAACTTGCGTCC
CACAAATCACGATTAATAATGAAATAGACAACCAAATATACTACACAAACATATCATAATGAAAATGAAGAATCAT
AGAGAGAGAGAGAGAGAGTAGAGACAAAGCTGCTACTACTACTACTACTACTACTCTGCCGGTGGTAACAAA
TCACAGCGGAGTACAACAAGATACAAGGTTCATCTTCCTTCATGGTGTCAGGTGGAGGGAAGTGTCTAATTCTCTT
GCACGTGAACCATCGTCGTGAGCAGCGCTGGCACGACAAGGATTACAAAAAGGCTCATCTCGGCACCAGATGGAA
TACCTTTACCTGGTCGCTTGCATCCTTTATTTTTTTTTTCATTTTATACCACGGCCGTGTAGACCGAGTTTTTCGAGTG
GACGAAACCTCCAGCCTTGTTAGGGAGAAGTCTTAGCGGATCGCAGTGAGGATGCTGCTCTGCCGCCCACAACAC
AACCTCCAGCCAGCCAACTGTCTATCTGCTCACACACACTCACACACTACAGGATGTATCGTCGACTCTTGACTGAC
ATAATAATAAAAAAAGGCAAACATATGGAAATTATCAATAACAAGAAAATGCATGAAGTCTGTAATATTACTAAAC
ACCGAGAATTGCTTTGCTTCAAGTTCAAGATAGACATTGTGTAGGTGAAACCTGCTTCTCCTCAACTAACCTTGTAC
TATACCTTTAACTGGTAGCTTGCATCCTTTATTTTTTTTGCATTTTATACCACGGCCGTGTCGACCCAGTTGTTCGAGT
AAAGTTAAAGTTGCAGAAATATCGAAGAAATTACACTTATACGAACATGTCACGAGAAGAAATTAAAACAGGACTA
CCTTAATGGTGCAACAATCATTATTAATGCTGTATTTTATTTTCCAGGGATGCATGAGGTCTGGAAGGTGTCATGGA
GTGTGGTGGTGGAGGTGGTGATGCAGTGGGCAGCGGTAAGGACTAGTGATTCGTGGATTAGGGAACCGCCACA
TTAACATAGTAATTAAACAAGCAAGATCATTAATATAGATGCTTGGAGAGAAAATACTTTTACTACCTTCTTTCAAT
GATAAGGGTAAAAGAATATATAAACTCACCCTAACTCTAAACACCTCACTTGCTCTTACTGGCTACTTACGAACATG

GAAACTAATGATTATGCTACCTTTGCACGGTCAGAATACCGCGGCTCTTTAACTTCTTGTCAGTGAGCAGGCTAGA
AATAATGATGTCAAAATAACACTAACGTTACTGACACACAATGGCTTCTCCCTGCAGGTGGTGACGTCACTCCTG
GAACTCAAATCATGTAAAAATTTAAAGGTCGAACAGACCCTCTTTTATAGCAGCTGCACTATAAAGATAATTTTAAT
GTAGGACATGCCGGTGTATAAGGTCCATGGTTTTACTCCAGTGATAATCAATGAAGAGACCTTGCCCGACAGGT
GACATGTCTTGCCTCTTTATCAAAATGATACATGTGTTACTTCTTTATGTATTGGCACAAGATTCAAGGATAGACCA
TCAAATAGACATGTTTTTGATAAACATGCAAAGAAACTTGTTTGCCGAATTCCTTTAACATAACTTTTTTTTATTTAT
AATTTTTACATGATTTGAGTTCAGAGCGGCGTGAGCCAGGTCGGTTTCTATCTCTTGAGAAAAAGGACAGTTATTA
AAATTAAACCTATGGCAACATGAGCATACCTTAGCTTTCAAGACAGAGCTTCACCTTTAATAGAACAGTTAATTTTT
CGCTGACCCTGACATCAAGACATTACCTCAGGTGTTGCCAGCAGTCTTGCCACACGAGCCGTTCATCTCCCCGCCT
AGGAGGCGGGTGAAGCAAAACCAACAACAATAAGGCTGATGTGACTGAATAGTTGTTAAGTTGTGATATTCAAG
TATAGTTCAGCTTCTATAAAAGAGGGTCTGTTCGACCTTTAAATTTTTTCATGTTTTGAGTTAAGTCCGGCGTGAGC
ATAGTCATACTACATCGACAAAATGTCAATATCAAGCAGCAGCTTCGAATCCTACATGGTGGTTAGAGGAAAAATG
TACACTGACATGATTTAGTTATATGGAGAGGATGGAGGATGAGAGAGTGGAAGGGGAAAACAGGTCTAGGATA
AGAGGGAATTCTTCTCCTGAAACCTGAGAATTCTGAAGCTCCAGGTCTGTTTTCAATTCCTTGGAATCTACTGTGTA
GAGACCCAGTGATTTTGAGACGCTCACTCACAACAGAGGGAGTGATGAGCTTGTAAGATGGCACCTCCTTCAACA
ACATAGAGGTCGCAAACTTTTTTTTTCGATATGAACTCTCAATAAAAATAACTCTGTTATCCGTAAAGTATCTTAGT
TCACAATTTGATCTTTTGTATCTGTATCCTCCAGGTTGTTCATCATATCATATATTGTAACTAAATCTCCTCTTTCTC
GAGAGGAGGAATGCAGATCTTTGTCAAAACCTTGACGGGTAAGACCATCACTTTGGAGGTGGAGCCTTCAGACA
CGGCGGAGGTTCATGTCCGCTCCCCTCTCTAAAGAACTGAGGCAGAAATACAATGTTCGCTCCATCCCAATCCGCA
GAGAGGGAATTCTTCTCCAGAAACCAGAGAATTATGAAGCTCCAGGTCTGTTTTAAAATCCTTTGAAACTAATGTG
TAGCAAGAAGACCAGACGCTATAACTGGTCGGTGAAGGCCATCCGAAGGAAGACCACTGGCACTGGCCGTCTGC
CACTTGTTGCATTCCATCTTCAGCACAATCTTCTTTGTGGTCTTTGCCTTCTTTCTGAAGATAGGCTTTGACTGGCCA
ACGAGCTGGCATGTGTTTACGCGTCCCTCATCTTAATGGACGATGAGGTGCCCATTACGGCTGAAAAGATCTCCAC
TCGGTGAAGATAAAGAAGAACCCGGACAATGTGAAATTCAAGGTGCGATGCTCCAAGTACCTTTACACCTTGGTG
CATGGACTTGAACACCGCAGTCCAGGAGGTGCTCAAGCAGGCCCTCATGGCAGGTGGTCTGGCTCGGGGACTCC
CTCTTCTGCAATGGAGAGCTTGATGCCCTTGCCGCGGGGGAGGGACACATAGGCCTTGTTTCCTTTACCGATGACA
CGGCTCTACGTGGATTCCTCTGTACGCAGTCTGGCGGATCCATTGGTCAGGCGGATTGAAAGCTGGTTGGCTCGC
AGTGTTATGGTTATTCAGCCCATTCTAAAGCTCACTGTCCCCACTCATAGTAAAGTCCGAAAAGTAAGATTAATAAT
CGCTCTTTATTGAGCCACAGGGTCTGCACACGACCACCAATATCAGATGTCCACACAGAGGTAGCAGCTTACAGC
CACGCCGCCCTTTAGGAAGACTGGATGGCACGCCGCCCTTTAGGAAGACCAGATGCCACGCCGCCCTTTAGGAAG
AACTTAATTTTCGATTAACCTTAAACAAACATACTCTCTAAAACTATATACCTTAAAATTTTAACCGAGAACTCACCC
AATCCAAGATAAGGAAGGGATTCCTCCCGACCAGCAGCGACTCATCTTCGCGGGCAAGCAGCTTGAAGATGGACG
CCTCATCAGGAAGTACGGCCTCATGATCTGCCGCCAATGCTTCAGGGAGTATGCGGCTGACATTGGATTCAAGAA
CTAAGATCCAGGACAAGGAGGGTATTCCTCCTGACCAGCAGAGACTCATTTTTGCTGGCAAGCAGTTGGAGGATG
CGTCACCTTGCCCCAGATCACACGCAGTTTAGATTTCCCACCAAGAGGGCAGGGAGTCTTGTTTTTACACTTGTAG
CGATGACCGATGAAGACCATGCCCATCCTCTTCACGCCATGATGACCGACAGAGACCATGCCCATCCTCT
TAGGTTGGTGGTCCACTTCTCGATAGAATTGATCTTGACGTCAAAACGTGGGGAGATCACGCCACACTTGATGAG
ATAAAAATACCGAAACTAATGTTAGTTTTCAGCAATCCCAATAGAAACTAAAGTAGTAAATAGTTTTGTTGAAGG
GCGGCCACCACTTGCTGCTTTGCACCAGGAAGATTGTGCTTGCGGGCGAATTTCATGTTTCGCAAGAATTTTGGTT
TAACGCTGGTGGTCAGCATCACCACAGAATTAACTTTGACAAGTACCATCCAGGATACTTTGGAAAGGTTGGTAT
TTAGAGACTTTCTTCTTGACTGGTTCATCCTGTGCCTTGGCCACCACTTGCTCCTTCTCAGACAGGATCACCTCAAT
GATGTTAAGTGTCCAGGCTGCTTCAAGATTTCCACAGTGTTCTCCCACGCCCAGACCGTAGTAGCTTGTGTAGGGT
CGGCCATTAAGGGTGTTGGGAGGCGGTACTCCAACATTGTCCTGAAGAAGGCAGACATCGATCAGACCAAGCGTG

GGTCACAATGTCTCCAGGCTTAACGTCCCTGAAACAGGGAGACAGATGCACAGACATGTTCCTGTGGCGCTTCTCA
CGGCGGGAAAGACGAACTCGGATGCGGAAGGGAACATTCCTGATTCCCCTGCACCAGATGTGCTTGTTCAGCCT
AGAGTCTTGACAAAGATCTGCATTCATCCTCTTAGCCTCAACACAAGGTGGAGTGTGGATTCTTTCTGACTGTTGT
TTTTCACCCAATTTTTAAAGTTTACTTCAGCCCTTGATACATTGATGAAATCACTATGCAGTTTTGTTTGGGTTAACA
TTGCTTGCCTTGGCAGCTCTGTTCTTCTCCTTAGCGGCCCTAATTGCCTGTTCTCTCTGGGCCTTGCGCACTTCAGG
ATCAACTCGACGGATGTTGATTCTTACGTGCGATGGAACAGTGCATGGACGGAGATGCCTCTGCCTAATCAACAA
GAGGCTAACATACCGCAGGCGGCTCTCCTACAACACCGCCAGTAACAGGAGGAAAATTGTGAAGACCCCTGGAG
ATTTCCCTTTGGTAGCGACAGCATTATTCCCCTCAAGAAATTTGCATCAAGGCTGGATGTCATATCTAACTTAGATT
TGCCACTGTGCCCAAGACGGAGATCAGGGAGAAACTGGCTAAGATGTACAAGACCACAGGAGACGTCGTGTTCT
CAAGTTTGAGGAGATCGAGGACGCCTTCTACATGGTGGTGGGCAAACACACCACCATGAGTGACGGCAACATTG
AGGCGGAACTTGGAGTCACGGTCCTTCCTATTCCTTTCCAGATGCTTGCGGATAAAGACAGCCTTCTTGATAAGGT
GGTAAAGGTAAAGGTAAAGGTAAAGGTAGACCTGGCTGCAAAGGTAGACCTGGCCAACCATGTATATCTGCGCC
CTCTCTTCTTGTAGAGGACAGCAGCCTTGAGTTTCCTGCGAGTCTCCAGAGTGGCCACCACACTCTTGTACTTCCA
CAGAGGACTTGGAATCTCGCATGGAGGAGCGCCTGCGACGAATCTCCTCCTGCCTCTTCTCCTTGGCTTCCTTCTG
CTCAAGCAGCTTGCCCAGGTCAAAACGAGGCTTCTTCAGTACCTTCACCTTGCGGATGTGCACTTCCTTGAGCGG
GCAAAGAATTGAAGCAGCTGTACTTCTTCTGGCGTTTGCTGATACCACGTGCCTTCTCCTTCTTGGGTGTGCGGAT
AGCTGACTCTTCCTCCAGACGTTGAGAGGGCCGTGTTCTGGTCTGTACTGCACTGTCACGCCGTCGGGGTAGAGG
CGCGGATGTGGGTGCCAAGTCGTTTCTTGAGGAACTTAAGAGCCCTCTTGTCCTTGGAAACCTTCAACAATTCAAT
CATTGCTGTGCAGGGCATCAAGGGTCGCCTCAACCGTCTGCCCGCCGCTGCTGTGGGGGACATCGTGGCCGCTAC
CTCCTTCAGGTCCTTGTTCCTCCTGATGAATGACCTCACACCAGCAGCGGCGACGGTGGAGTAGGTGTAAGCTCCG
GGATCTTGTCAAGACGGCCAAATTCAAGGAGCTTGCCCCCTACGATGACGACTGGTACTACACCCGGGTGTCTGC

GGGACCTTTTCCTTCCTAAGCACCCGGAATTGGAGAAGATCCCTAACTTGCATGTCATCAAGGCCCTTCAGAGCCT
GGATCCTTCAAAATCTCCTTATTAATCTTAACCTTGTCAAAGACGATGTCAGTTGCAGTATAACGAGTGGGCATCA
AACTGTCCCAAATGCAGTAAACCGGTGTATTTCGCTGAGCGCAAGACATCGCTGGGCAAGGACTGGCACTCGAGT
TCAGACACATACACTCCGTCCAAGAACTTACGGATGTCCTTCCTCTTGACGGTGGTGCTCTGCTGGATGCGTGCGG
GCGGGGCTGGGCCGGCTGGCGCTTGCAGATCACCTTCTTACCCTTGGCGTTGGCCTCGTGCTTGATGCGGTCGTT
CAAACAAACACCATACACCAAACGTTTGTAAAAAGGAAAACGTTTTGCAATACAACACAACGTTAGCAAGCAATA
CCTCCGCCTGGTCACTCAGCATCTTGGCACGTGCATTATCGGCCTTCTTCTTGTGAATGTATTCCATGAGGACGCGC
AGGCGGCGCGGGTAATCATTGAGAAATACTACACCCGGTTGGGCAGTGATTTCCACACCAACAAACGCATCTGTG
GTGAAGGCAAGACTGATTATTATGCTCGCAAGCGCTTAGTTGTGCAAGCCAAGAATAAGTACAACACCCCTAAACA
TTACATGATTGAGTTCAGACCGGCGTGAGCCATGTCGGTTTCTATCTCTTGAGATAAAGGAAAGTTATTTTTGTACC
CAAGATGAAGACTCAGACACTGGCGGCGGTGATGGTGGTGGTGCTGATGATGACAGTGGCCACAGAAGGTTATG
TCTGCCACATCTACGCCAGCTACAACGACACCTTTGTCCACGTTACTGACCTGTCCGGCCGGGAGACCATCGTGCGT
AAGGTCGACAGTGTACTCAAGGAACAGCTTGAGGGCTTCTCTCCTGACAAGTTGAAGAAAACCGACACTGCAGAG
TCTCGGAGATTATTCTTCTTAAGATATTTCTTGGTGAGATACTTCAGATATCTTTTGGAGAATGGAATATCAGCATTC
GATGAGTCTCACCGATCCATCATCCGGAACGTTAAGGGACCCGTGAGGGAGGGGGACATCCTCACCCTGCTGGAG
CACGACCTGTCATGAACTGGTCCTCAAGGCTAGATTCCACCTTGGCGGTGCGCTGACGCTCCTTGTATTTCTTCTCG
GACACGGCCGCATCAGTACAGCTTGGTTTCCTGTCGCTGTTCTGAGCTGATCTTGTGGTACTTGGCGAGGTAGTC
GAAGTATTTACCACAGGCTGTTCCAAGCTCGATGTTGTTGCCAGAATAGTGATGCACACCAGTCTTTGCCAGCATA
CGGTGCTCCCATCCACCGCATCCGTATCACCTTAACATCCAGCAACGTTAAGTCACTCGAGAAAGTCTGCTCCGAG
CCATCAAGAAATTTCAGATTAGGAATATTGTGGAGGCTGCCGCAGTGAAGGATATCAATGAAGCCTCAGTGTACTC
CGGTCATCCTGCCAGTGCTCTCATCCACCTCTGCAATGTTGATCTGGATGGAGGCGTGATCCTTGGCATGGATGAT
CCTTCCTACCGGCACACTTTCCTCTCGAGACCGTAGACGCCTTGCCGATGTGTTGATGATTACCACCTCCGTGCGGA
GTGGGTGAGGTAAGGGAACTTGATCTTAGAGTTGTGGAACTGTGTGGTGAGTGGACGACGGCACTTACCACTTGA
GAAGGGACTCTTGAGGGAGAAGTCAATGTGCTTCTGGGAGTCAAGACGCACGATGAAGGAGGGAACATTGACCA
AGAGCATTGGTGGGCCGGACAGCTGATCCCAAGTTCATGCCAGATTTCTCAATCCCATATATTAGTATTTTTTTAAT

GCAGCTGGAGCAGCAACAACAGCAGCTGCAGTAAACTTGCTTGGATCAGCCAAGTATTCCTTCAGTGTCTCTGCC
TCAATAACAGATACACATGCAGGATCTAACCTAACCTTACCTAACCTACGCAAGCAAGATCGTTAATATAGATGCT
ATATACAGTATAATTAAAGTGCATTAATTTTAGTGGCGACCCTCGGCATGTGTGGTTGATCCCTTGCTTCCTCCGC
GACCAACAAGGAAGTGGTCCCGGCCGTACAGGTCTATGGCAGGAAGAAAACTGCCACGGCCGTCGCGCATTGCA
AGGTGGTGTGCGCTTCGGCAAACCCAAGAGTAAAAAGATAAAACAGGTGTTGGAGAAGCCCAAGACCTTTGTG
CTTGGAGCGCACCAGGGGCTTGGTGTGGCTATGGGGTAGGCCAGGGGCGGGGCCGAAGCTCTTGCAAGCCTCA
CCCTTCTACCGTTCACGTCCATCCGCATCTATTTCAACCTTTCCCACGTGGATCTTGAATATATGGGTGACGCAGG
GCCGATGTATTGGATTTGATATCCGACCCTCGTGTAGGAGTGACGGCAGCACATGAGAGTGCCGCCGCCGCAAT
CCCGTAGTCTTCCTCGGGGTGGAGAGACGGGCAGCCGAGAAGTATCTTTCCTCGTAGTCTTCCTGGGGTGGAGAC
GGCCAACCCCTTCGGAGGCGCATCCCACGCTAAGGGAATTGTGCTTGAGAAAATCGGTATTGAGGCCAAACAGC
TAGAGGACTGTTTGACTAGGCTGGTTTGATAGAGTGGTTTGGGTGATAAGAGAGGGAAATCTTCTACAGAAACC
CTGACCGGTACGTAAGTCGTCTGCGAACGATTCGGTACGACACATCAGCATTCGGGTGTTCTCTACTAGGGAAG
TCACGCCGACTCATACATCCTGTTATCATGAAGATGCCAACCGAGACCATGAGAGTACCAGCATCACCATCACCAT
ACACATCAAACTGAATATTGAGAGCAATGTGTTACTCAAGGAAAATGAATACATTATGGATTACAATTACACATCA
CTGGGTGGACGCCGCTGCCACACTTCACCTTGCAGCTGTTCCATTATGCGGTGCTTCAGAATATGTTGAGGAAGA
TGTAGAGGACTGGTTGACTAGGCTGGTTAGAGAGAGTGGTTTGGGTGATAAGAGAGGGAATTATTCTCCTGAAA
CATTATCAGAGGAATGTGCAGGAGGGAAGAAGAAGAGGAAGGTCAAGATTGAGATGGAAAGATAGTGGTACG
AGCTACAAAATTACTTAGTCTTTCCAGGAACGAAAACGGGATGAAAAAATGAATACAGGTAAGTCTTCAAAACAT
CACAAAGGTCCGGATCCAGCCGTTAGAACTTTAGTCTTCCCTCAGAACCACAGCGAAGGAAGCAAATTTTTAGGAT
AACAGATACACATGCAGGATCTAACCTAACCTTACCTAACCTACGCAAGCAAGATCTTTAATATAGATGCTTGGAG
GGCTTAACGAGGCAGCTGGTGACGGTGGCGGTGGTGGTGTTGGCAATGATGTTGGTGGAGTTAGGGAAGGTGG

GGTATATAAACTACGGCTTGACACTACTTCGGCCTGCGGTGCGTCCTTCAGAGCTGCTCCCTGCCCTTCAGAGACGC
TTCAACATCGAGGTCGCAAACTTTTTTTCGATATGAACTCTCAAAAAAAATAACGCTGTTATCCCTAAAGTAACTTA
GCCAAAGGAACCAACGTTAATCGCTGTTAAGTAGTCTTCACCTGTAAATACCTCTCCCTCAAAGAACGTGGCCAAAT
GTACATTTTGCAGGAAACGTATCGAATTAAAATGATCTCCAAACATAGTGTTGTCTTTTCGCAAACAGTATC
TAAATTTAAAACTTATTTTATTTTAGGGCTTTAATATACAAATAAACTTATAATAATAAAACCATTAAACCCCTATAAA

TTTCACGACCATATTATACTTGTATTAATTTTAATTATCACCTTTGTCGGGTATATAATATCTTCTGTTTTATTCAATTT
CGCCCTGGTGGTGGGTTGAGACCTTTCCCTCGTCCTCAGCCTGGTGGTGGTGTACGTCCTTTACCCCAGCCTTTCCCC
TCATTGCTATACATCCATTATAGAAACAAATGCGCTGATAACCATTAAACTAAATAATGGTTTTTGGTTGTATACTAC

GACAATTAAAGAGACGGATAAAACAAATAAATAAAAATCTAGTGCCAATAATAACATGAAGGCCATGGAAGCCTGT
GAGTAGATGGATGGAGGAAAATGTTGTAGTAAGACATAGGTGAATGTTTAGTAAGTGGCATGGAGGTATGTATTT
TTTGATAGTTTCTGTTGGTGTGTTATCCTCTGCATCACCTTCATCTTCATCAGTTGTTGAAGCTTCTAGACCGGTATC
AGTTTCTCATATGTGGGCTTATCGAACAGCACAAGGTTGTTCAACTTGTCACGAACCTTTCCCTTTGACCACTTCTTCT

TTCTCTCATGTAGTCTCATTTCATTCTTCCCTTATCTTGTGACATTGCAGACCATTCCATCAGGGGATAATTATGTCAC

AGGATGATGAAGTACAGGTAGTCCGAGGCCACTACAAGGGCCAGCAGGTAGGCAAGGTGGTCAGTGTGTACCGA

GTCACCTCAAGATCGTGTACCGGCGGTTCAGGAATGGCTTCCGTGAAGGCACAAAGCCCCAACCCAAGAAGTCGG
CCATATCCAGACTGTTTCCTGTCATAACGCCTCCTGCCCTGGGCGTATGTACGCTCCTTGGACTTCTTGTACTGAGTC
CATCCTCAAGGCCTCCAATGTCACCGTTGAGCCTTACTGGCCGGGCATGTTCGCCAAGGCAGCCGCAGGCCTCGAC
ATCCACGACCGGGAGAAGGCCGAGAAGCTGAAACAGTCCCTCCCTCCAGGTCTACAGGTCAAGGAGTCAAAGTAA
ATGAAGCAGTGAAAGCTCTTGACAAACGCAAGGCATATCTGTGCATTCTAGCCAATAACTGTGATGAGCCTGGGTA
AAACACATTATTCAACCTGGTAGCAAATGTATGGCCGACAGCATCCTTCACATGCACAATGTCAAAACTCCCAGCAT
TCGACGATCTCTTTACGTTTGCGAGATGAGACTCCGTGTGCGATCTCTCCACAATAAGTTCTGTTCTGCATCATAAGA

TCAGCTTGGTCCTCCTCCAGTGGCGCCTCTTGGCATTGTACCTGATCTTGTTGCCCGTGCAGTTCCTAATCCATGGTC

CTAACCGCCTTTACAAATTTAATTAAAGTTATTTTCAAATTTTAGTCAAAGAATTTACTTTAACTCTTTAAATACTACA
GCACCCTTTCCGACTACAACATCCAAAAGGAATCCACTCTTCACCTTGTACTGCGTCTTCGTGGTGGTGCCAAGAAG
GCTGGATTAAGGATGGCGAGGGCACCTTATGGGATGAGGGACCAACCAGCACCACCATGAGCCTCACCCATGTCC
GGACGCACCCTGTCTGACTACAACATCCAAAAAGAGTCTACTCTCCACCTTGTGTTACGCCTGCGTGGAGGTGTCAT
ACATAGGCACAGCGTTTCCCGACGTAGTATTTGCCATCATCCTTGTTTTTGGCACCATCCACCTTCAAAAGGGCTGTC

CCGCCCAGTGAGGTTGACCACAATCTTGCCTGCGCGGTGGTCGTCCACTATTTCAAACTCTCCGATGTAGCCGTGCT
CAGAGTGCAAGGATTTGCGAGACAACTTTTACTTGTTCTTTCAGAATCCTCCAATCTAATCAATACCCACTTATTTTCT
TCAACTCCTCGCATGGATGGATGCTTCTGGTTCTTAGGCCGACGGATTGGGTTACGGTGAGCTTTCCGTGTCTGGTT
GAGGAACTACCATGAGCGCCCCAACCACAGCTGGGCTCCCATCATTAACCTGGACAAACTGTGGTCGTTGGTATCC
ATGACAAGGGGAGGACATGAATGGGTTGATGCGACCGTGTGCCCTGTAGGTGCGGCGACGCATCTGGCGGGCAC
TGCGCGACAGTACTATGTCAGCCCACTGGAGGGAAGGCCAAGCTCACAGATGGGTGTTCGTTCAGACGGAAGCAG
GCCGGTGAACTAACTGAAGAGGAGGTTGAGAAGATCGTGACAATCATGAGCAACCCCCGTCAGTATAAGATTCCCC

AAACCTGTTGTACTTAAAGACATAGTGGAGGTAATCTCTCCTGATGGTGATGGTGCGCTGCATCTTCATCTTCTGTAC
GACATCTAAACGCACATCCTCAGTGTTCATTTGTTTCTTAGCAAACTTGCGTATTTCTTTGATAGCACGAGGCGCCCT

AGTGCTAACTCTCAGAAAAACTTTCGTGGTCCAGAATAAGTCGAGTAGTACTGAACCAGAGCCACCCGGCCAATAC
CTTCATGTTTCTTTTGGTCATAATATCGATGAGGGATGCACCCACAATAGCACGCTGGTATTTTTGAGTGCGTCGAGT
CGTGACGCATGTGACGGTGAGCTGAGGTGCCCGGTGGTGGCAGGAGAATTGACACAGTTTACCTACTCCCTCAACA
GCCGTCTGGTGTACCACTACCAGAAGAAGCCTGGCAAGGCTCCAGCATGTGGCAACTGCAAGAGGCGTCTGCCTGC
CAGTATTTCACAGGTTTATCACTTCCAAAAACTTCCCAGATTGTTGGCTTGGAATCAATAAGACATTCCATGATACTT
GCTTTGGGTTCCGCACACAGTTTGGTGGCGGGAAGAGCACCGGCTTTGCTCTCGTCTACGACACGCTGGACTACGC
AGCTGGCCGGCGCCAACCCTTCTGCAGAGGAGGCCGACGAGGGTACTGAGAGCGGCAGTGTGTCCGGCATTGAT
TGGTACAAATCCTCTGGGATATCTGGGGCCAAACCCTTGGCCTTAAGCACACGAAGGATCTTGTTACCAGTGACGA
AGTTGGTCCACCTGGGCCATCTGGCCAACCCGGCCAATCCGGTGCACCTGGCCCACCCGGCCCACCTGGCCCTCCC
CCCGACCTCATGGGACAGGCGACCAAGGAAGCAGTACTTGCGTCCTGGCTTGAGGCGCATGATTCTAAGGGCATC
CCTGACAGCCAACAGCTTGGCATAGTCAGCCTTCTCCTCCTTGTTCTTGACGGCACGCTGCCTCTTCAGGGCCTTCTT
TAGATGAGGTTACACGCCTTACGGATATCATCAGCCATGGAGTCTGGGATGAGCTTATTCACCACTTCCTTAAGCTC
GTGGGGCATCACCACGCCCTTCATCTGCTGGGCCAGGGCAACCTCCTGGGGCGAGGAATCGCTCTTCTTTGGCTTC
CGGCCTTGTGCCATCAACTCTTCGTACTCCTCGCGGTCGAATTTAGTGAAACCCCATTTTTGTGAGACGTAGATCTTC
GGTACGCTTCTCATAAGGAGCAAAGCCCATAACCTCTCGCACCAGATCCCTCACAAACTTGTTGTGTTTGGTATTGT
TGTTAAGAAGGGAAAGCCTGAACTCAGGAAGAAGGTCCACCCTGCTGTAGTAGTGCGGCAGAGGAAGCCTTACCC
GCCAGCTACAGTGCGGCGGCATGGCTTGCAGGACCAGATGCCAGTTGCCATGCGTCGCATAGCCTCCTTGCCGCAG
TGTGGCCCGACACATCTACATGCGCTCACCTGTGGGTGTAGGAAGCGTGCAGAAGATTTTTGGAGCCCGTCAGAGC

CAAGTCCAGGGGTTATGTGGAGCAGAATTTCGCGTGGCGTCATTACTACTGGCGTCTGACTAATGAAGGGATCCAC
CATGCTTCAAGTTCACGATCCTGACAAATGGCTTGATCTTAGAGCGACGAGCAATCTTCTTCTTGCTCATACTCTTGC
TGTCTTCGATGTGATAAGTGCAACAAGACCCTCACGCCAGGGAGCCACGCCGAGCACGACGGCAAACCCTACTGTA
AAGTCTGAGAAACAGCCTCCACATTGTTTCCTTCAATCACAAACTCATCCTTCATCTTGGCGGAGGCTGCGATGGTG
CTCCTTGACGCGAGCCTTGAAGTCCTCACGGCAGCGGGAGTGTGTAAGGTGCTCCACTCGCACGTTGAGCCTCTTG
TTTGTACGAAAACGTTGCAATTGTGAGGAAACGTTTCCCTCTGCTGTAACTTGAGGGTATTACAGCAAACGTTAGTA
TTGTTCTTGAACACGTTACCCTTAACCTTCATGTACAACTCGTGGTATAGATGCCTGTCGATCTTCTTGTTGTCACGG
AGGAGATCGCCATCATCCCCTCAAAGCCCCTGCGCAACAAAATTGCTGGATTTGTAACCCATCTGATGAAGCGAAT
ACCGGCTTATTGTGCGTCTTACCAACACCGACGTCATTGCCCAGATTGCTTATGCAAGAATTGAAGGAGATGTGATT

GATGGTTGTAACATAGCTTGTACCCTGGAGTATGCCCCTGTGTGTGGCAGCGACGGCGTCACCTACGGGAATTATTC
TATTACTGGAGGACAGAAGGTGAAGGCCGACAGAGATGAGTCCTCACCCTACGCTGCTATGTTGGCTGCTCAGGAC
AAAACTGCCTTGCCGACAAAGGAGGACGTGGCGGCAGAGAAACAGGCTCAGGCTCACCTCCAGGCAGTCGAGGG
CAAGGTAATCTTGTGCTTGTTCCTCTCCAGTGTCACTTGGTTAGAAAGGTTGTTGGTCTTTCCATTCACCTTGATACG
GTCTGAGCGTGAGGCGCGTAGATTAAGGTGATCGCAGCACCACTCGCCACACGTTGAACAGGTTATCCAAGTACAC
GGTCTTCTTAGACCTCTTCTTGTTGAGGACAGCCTCCTCAGCATCAGTCAGCTTGGTGGTCTTCTTACGGCCGAGAG
CGTGAACTCCTGGTAGGGGGACTTAGTGAACACAGTCTCCTTCCATAGGTCGGGTGTGAGGTAGGCGTATGTCTTC
GCATAGTACTCAATCTCGGACTTCCTGAGTGATGGAGTGTTGTTGGCAATAATGACAAGCTTTGCCTTGCCAGCGCC
CTGGTCCGCGGCGCCAAGGACAAGCAGCTGAAGGTGAAAGGCCCCGTCCGCATGCCTACCAAGACACTTAGGATC
CCATCTACCAGCTTCCCAAACTGTACATCAAGCAGCACTACTGTGTGTCCTGCGCCATCCACTCCAAGGTGGTGCGG
GCGGTTGGAGGCGGAACACTTCCTGGGGACGTACAGGTCCACGTATTCTCCGGCGTCGTTCTGCATGTTGGTGGT
ATGTTCGACAGGGTTCATGGCCACACCGCGCACCTTAGGCCAGCTGTTCCTCTTGACGCGGTACTTGTGGTATGCGC
ACACTGTCTCCACACGCATGATCTGAATAGAGTGTGCGCGTGCCCTGTGTCTGGCCCCCATATCACGATAGCACTGT
CCTGCTTCCTCACCCTGATGTGTCCCTGCTTGATCAGAACACGGGCGTGGTGGATTGACTTAGCCAGCCCCAACTTC
TCCTTTTCTATTTTTCATGTCATTCATTGTTACTAACTACTACTGTTGTGTTTGCTTCTTAAGTTATTATAGACCTAAGT

TCCTTGAAGGTGATGTCTGTGACAGCAGCAATAGACAGCAGCTTCTTGAAACCATTCACAATTGAATGAGGCACCG
TGGAGAGAAAATACTTATACTCTTCTTTCAATAATACACATACAGGACCTAACCTAACCTAACCCTAAGCTAACCTTA
CTCGCCTCGCCACCCTCCCTTTACCCTCCCATGCTGGTGAGCCTCACTACTCTCTTATCATACCTCGCCCACCACCTCA
AGCGTGGCCACGGTCTTATCAAGGTGAACGGAAGGCCCTTGGAGTACATTGAACCAAGGACACTGCAGTTCAAAC
GAGAAGCCCATTGGAGGCGAGAAGAATGGTGGAACCCGTAAAGTACGCGTCAAGAAACTGCCCGCTTACTACGCT
CGTGCCTTCCTGCGGCCTTGAATGAGGAGTGTGTTCTTGCCAGTTGGTGAGCGTCTGGCCAGCATGTCAAAGGTCA
CTGTACTGATAGCCAGCAAGGATGGATGGATGGTTATCTTGTACGCTGGTGACGTGCGTATCGGTAAGACCAAGG
GTGCGTTCGACTTGTCGATGTATCTGGTATTCTTGCGATTCACATACCGACACGCAGCTTGCTGCGGTCTTCATCGAG
CACGGGCAGCCGAGAAGTATCTTTCCTCGTAGTCTTCCTGGGGTGGAGAGCAGTAGAAATACATTAATAAGATAGA
CCAACTCTGCCATTCGTAAGTGTGTTCGTGTCCAGCTGATCAAGAACGGCAAGAAGATCTCTGCGTTCGTGCCTCG

GGAGAGCCGGAGCCTGTTTGTCGAGACTCTCCCGAATTCCCTCTGCACGGGTCCATATTCTGGCCCCGCCCGCGAAG
TCACGAGAATGAAATAAAGAAGAAAAAAATCTTGCGAAACTTGAAAATGTTATTGCGCCTGCGTAGTACAGGGAAG
AGAATTAGTTCTGAAAAGCATATAGCTAAGGACACACACATCACAAGGACAATCTAAAAGGAGGAAGGCTAGGA
TACAGATGATCCAATGATCCTAAGAACACATTAAAACACGCTATTCATGGCGATCAACAATCACTTGCCACCATCAG
CCTGAGAATTCTGAAGCTCCAGGTCAGTTTTCAATTCCTTGGAATCTACTGTGTATTTGTTAGTTTCTGTTGGTGTGT
AAATATTTACATGGTGAAGATAGCAATGTTTGATTAAGGGGAGAGAGGAACCATCGGACAAGAAATATTGACGTT
TTCTAGGCTTCCAGAATTTCCAGACTGATCTAAATAGGTTTGCTTACTCTTATTTCCAGTGTTCTTGGTATTGGTTATT
TGTCAATACATAAGAATCTTTGACGCCATCTTGGAGTAAAATTTTTCCATCGCAAGGACAACAGATCGA

GAAGCGAAGGAAGTGAAGACGGTATCCGGTCATCCCACCCCAAAGATCCCAACGGTTGGACTATGGAATAAACAAG

CCTTCCCAACGCCCTTCCCTCCGCAGCCTCTTAACCTCTTCGCCGTCCACTTCACTTTCTTCCCCGTTTTCTTTCCCTTG
AGTCTTAAATCTTTATCTTAAAGGATCATTGAAATTTCAATCAATTATCAGTGTTCTAACTTATAAGCAGTTACAAAA
TCTAGGTGATTAAATACTTTGCTCTTGACAAGGCGAACAGAAGAAGTCGTATTGCAATATTCCTCGGCTGGTTCTT

TTTTTATTAACCGATATATGCTAGAACACCAAATAATTGAATTAATTTGAACAACACTACCAGCTGTTATTCTTATTT
CGGTGGCGGCATTCGGCCCTTCCCTCGCCCACATATTGATTACAGCCGATGCTCCACCCTTTGCTCGTCTTACTACC
GCAGCAATATGAAGCAATTGAAAACTTTGGAAATCATAAAATCAAGCTTCCGAAATCCCATGAAATAATTTTGGAA

TAGCTACAAAAAAAGTGGCCCCAAAGATTGAGTCTGCAATGGTAAAAGAGGCTTCAATATATTCATATGCTTGTAA
TTATTCGTAGCCAATCTGTGATGGAATAAGGTGTCAGACCTAATTCCTATAGAAGTTATTTATATTGTTTAATTTCTA
CACAACACTCACGTCTCTTGCCTCCACAAGCTGCACCACCATCAGAGTCACCGCCGCCGCAGTCACAAGTCGCCAC
TTCTTGGCCTTTCCTCCTCCTCCTCCCTCCTTCTTCTTGGCTGTTTGTTTAGCTGGCTGCTTACTCTTGGTGTCCTTCTT

CAATAGCTCATTTAGCATAAAATTTTTTATTGCACAATAACTATGTGTTCATATAACCTATTATTTCTCTAAATCTCGC

AAAAAAGTACTGCATCTACATCGAGAGGATCCAACGGGAGAAAGCCAATGGAGCCTCTGTCTATGTTGGCATTCAT

CCACGCAGTAAATGTTGCTATCAATAAACAAATAATAAAAAAAAAAAAAAAAAAAAAAAAAAATAAAAAAAAAAAA
CACTTTATGTGCCTGGTGCTTCTTGCACTTCCAGCAGAAGGTGTTACGTGTCTTGGGAATGTTCACCATTTTCCCAG
CCTGAGGGCAATGGTCAGCAATGTTGGCTCTGGTGTGAGCAGTGGCGGAGGTGGTGCCGTTGCCGCTCCTGCTGC

ACTCCAAGCTGGTGGAGGCCCTTTGCCAGGAGCACCAGATCAAGCTCCTCAAGGTTGACTCCAATAAGAAGTTAGG
GACGTTCACGGGAGACAATGGTGCCGACTCTGCCCAAGTTACGGCCACCAGTGATCATGCAAAGGTTGCCTGAAT
AACTTCCAATTCCCTAACGTTATGGAGGAGCACCTTACGGAAGCCATTGGGCAGCATATGCTTGGTCTTGGCAGCT

GGAATAGGACGGTTCTGCTTCATCTTCTTGGCCAGTTTGGTCTTAATACGGAAGGTCTTGTGGGCAGCCATGGTGC

ATTGCTTTTTATTGCCTGATTAAAATTAAATCAAATTAAACTATAATAATTATATACCTGCTAATAACAATAAAATTTA
CGCAAGAAGAAGAATTACACTACCCCAAAGAAGATCAAGCATAAGCATAAGAAAGTCAAGTTGGCAATGCTGAAC

TCGAGCCCTCCCTCAAGCTGTTGGCAGAGAAGTACAACTGCAACAAGATGATTTGCCGCAAGTGTTATGCTCGCCTT
GGTCTCATGCTGCGCTCGCAGACCACGCTTGTAGCCTGTAAACACTGCCTTGGCGTAAAGCCTTCCTGCCCTGTTCA

TTCATCATGACGGACAGGAACTTCACCACCACCTTGGAACACGGCCGGATCAGGACCTGCCGCTTGCCCCTCTTCTC
TAATCGAAGCTGGTTTCTAATCTTACTCATCAACTCCATTTCCCAAACATCCCAGTTTTATCTTTGCTTATTCCTTCAAT
TGTGATTGGTGTGGTTCTTAGACTTGGCCATGTTGTTGTCTGGTCAGACACCTCAATGAGCACACAGCAGTGATGG
CGAGCAGACCAGGACCAACTACGCCAAGGTGTCCAGGAAGGCGCCCGTCATCAACGTTGTCAGATCAGGTTACTAC
CGTTGGACCATGATGTGCTCGATCACCAGGTGGTCCACCTCAAGACCCTTGTACTGGGCATTGCTCTCAGCATTCTTC
GAATTAGGTTTGAAGTGAAAAAATTCCCTCTCTACGATGCCCTTGTGAGCTGTCATGATCTTTGGAGACACAATAAA
GACTGGTTCCTCAACAGGCAGAAGGACATTAAGGACGGCAAATACAGCCAGGTGATGTCTAACAACTTGGAGACA

CCACACCGGTCAAAATACGACCACGAATGGAGATGTTACCAGTGAATGGGCATTTCTTATCAATGTAGGTCCCCTC
TCTTCTTGAAAGAAATACCGTACAATCTCTTGTGCATATTGATGGTGTACTCACGGGTCACCACCTTGTCAATAGCC

AATTCTTATTACTGCAATGATCACCTGCTCCAACAAGCAGTGAAATTAAAACAGCCATTATTCATATTTCAAATCTT
TGCGTTTCTTGGTGGTCTCCTCCTCCATACCCTTCTTGTGCTTCCGCCTGTAGAGGATGGTCCAACGCACTTCTCGT
ATTCAGGACTTCTGGTTACGGAGTGAGTACCCTGTGATATGGTCGCTGGTGGACAGGAAGACAGACCGAGAAAT
GTATTGTGGTGGCCCGTCCACACAAGCTGCACCGCCTCTCCAAGCGTGTCAAGAGGGTCACCCGGGCATATGGTG
TTTGTCAACGGCCGAACTGGTAGGTTAAGTATTTCTGTAGCAGTGTAAAGTAGATAATTGTGAGGATCATCACAA
CCAAGAAGATTGAACCCAAGTACCGTCTTGTTAGGCAAGGACTCCTGGAGGTGAAGAAGACCGCCAGGAAGCAG
GTGGTTATATACATGCGCCTGCAGGAGACTGGTTTTGGCAGCAAGAAGGACTACCTCACCTACATGAAGGATTAC
ATCTCACCTGGGCAACACCATGACTGTCTCGTAGAATCACACCGATCTGTGAGGGCGTGAGGCCCTTCTTAGCCA
GGCCCGCCACCTATTGAACGAAATTTCACGGCAATTGGGTTCCCATGCCCCCTTGGCTCGAACTGCGAACTCATTC
TGGCACCACCTTCCTGGCAGACTTGTCATAAGGGGGCGGTGTGCCTTCAAAGCTCTTCAGGCGCTTGAGAGCCTC
GTGTCTCTTACGCTGCAGCACAACAGGAGTGATGAGGCGCTGGATCTTGGGTGCCTTTGTCTTGGGTTTCTTGCC
CACTGCTCGCCACGGCCCGGGTGATAATGTCAACCATCTTCTTGCGGATGTTCTTGACCTGTGTGTGCTGGGCGTA
GCAGCCTTCAGAGGGAAGAGGATCAGCTTGCTCTTGTACACCTTGAGGCGCTGCACATTCTGCTGCAAGGAATC
TGCCTGCCGGGGTACTTGAACTTGGCCCTCCTCAGAGCCTCAATAACATGGGCCTTATGACGGTCATGGGTTCGG
TCCCCTTGCGAGAACTGGGCTTGGGCTTCCGTGTGTTTTTGGTGACTTTGTGGCCTTTGCAAAGGCCCACAGCCAT
GCAGGAAGGATGGCGTCTTCATCTACTTTGAAGACAACGCAGGAGTCATCGTAAACAACAAGGGCGAGATGAAA
AAGGTGCAGATGTGCCTCTTGTGCTGCGTAATTTCCATCTTCTTGATCATCTTACGCAGGGAGGCGCCATAACGG
CAGAGGCACTGCCCCTTCACACTTCTGTAAGAGCTCTGGTGCCGTTGCCCGCAAGGCCCTGCAGACCCTGGAGAG

TACCTTCGTGATTACCTCCATCTGCCCGCTGAGATCGTCCCAGCCACCCTGAAGAAGCCCACACGCCCCGAGGGA
TCACCTTGCGCGGGTATCGCTCGATGCCTGCCACCAGGCCGTGTTCATAAGGACGGTCCTGTGTGCCCTCATCAT
AACACTCCTTGCTACAGCGCACTCTTCGGACCTGGTGGTTATGGTCGTGGAGGCACCGAGAGCTTCAAGTACGAC
ACGCCGGGTGCCATCTCAACCTTACGGATGTACTTCTCACCCAGGAAGTTACGGATCTCCACGGTCTTGTTGTTGT
GGGAGGATCTTTCCCTTGACGCGCTTGTTAACGATGACACCGACGGCGTGTTGGGTCACGTTGAACACTCGTCCA
ACAGCAACGTTCACCTACAATACAGTGTTAGTGTTGGCTAAACGTTTAATACAAGACAAACGTTGCTTTACGTTTA
TATTTTTTAAGCATACGACGCAACACTCGCATGCGACGAATCCACAGAACCTTGACGGGCATACGGGCATTGGCT
CCAGCGTGGACCAGTACGAGGCATCTCCATCAAGCTTCAGGAGGAGGAACGTGAACGCCGTGACAACTATGTGC
TATTGCATCAGCTTATGCCCATGAGCTTAGTAATTTTGGCATCAAGATGGGCTTTACTAACTATGCAGCTTGCTACT

GTCAGCTGAACGCGGAGGCAACGTGTAACAATCCTGCGCTCTACAAGGTGTCAGATGGTAATTGTCCTCAATGGT
GTAGCCGAGAAGTGCAAGACTCTCGGCATCAACTGTCTGCACATCAAGATCCGTGCCACCGGCGGCAACAAGAC
GTTCAAAGCCTCTTCGTTGAAACATGCCGATACCAAGGAAAAAATAGTTTTACCAGCAAAAGAAGATATCCAGAC
TTGTTGAAGGAACGTCTCAAAGTCAGCAGCATTCATGATGCCATCTTCGACAGGCTGACGGCAGTCAATGTGGAA

GAAGGGCGTAGTGGCTCTCGTACCACTGCCTGAAGGGTGCGGCATCGATCACAATGATGGCATTCTTCACCAGG
GCAATGGCAGCATAGGTAGCCTTAGCGAAGTTACCAAGAGTGGCAGTCTGGCCCCTAGCGCTGGTGTAGCAATC
GCAGGCACTTCAGGGTCTGTCGGTATCCCAGGACGTACTTGCCAGACTTCATCACCAGGGCCAATCGAGAGTTGA
CACAACACGCAAGACGCCCTGTGGTGAAGGATCGAAAACCTGGGATCGCTTCCAGATGAGGATTCACAAGCGTG
AACAGGAGTCGTAAAGCAAGGAAAATCCGCACTCCTCCACCTCGCTTCCCAAGGCAACAGGTCAACATACCAAG
GGTGGTGGTGGTTGATGGGAACACACACGTGGCTGTAGATGGAACGAGGGATGCAGAGGGAAGGGAATGGCG
GGCCGGCTTTAAGGATTGGTTTGTCTATGCGACCGCCGCCGGCTACGATGCCAATCATAGCCCGGTTAGCAGAA
GTCACTGCCCCGGACACAGTCAGGTCACGGTATTCACGATACATGTTGTGTGTACCTGAACGAGAGTCATAACGA
GAACACCTGAGTCTGCAGCCTACGCTCCAAAAAGTCCTCAGGCTTCAGACCGAGCACATAATCGAGCTTCATCCTG
TATTATATTATGACGGAGCCCATTGATGTTGAAGGGGGATAGTTGCAGTGTGAAGAGTGAGTGAGGTGCTTGTT

GACACAATGGTTGGATAGCCAATGGAGAGTGACAGTGAAGCCACTTTACACAATCCATCTTGGAATGACTTCATCA
ACCTAACCTAACCTAACCCTAAGCTAACCTTACCTAACCTAACCCTTCAACACTCAGACACACCCCAAGGATCTGACC
ACTCCACTCGTCATCATGGTGCGCGAACGTACCTTCATTGCCGTCAAGCCTGATGGAGTCCAGCGTGGCCTCACCGC
CTCATGGAACCTGTACTGCTGCTGGGCAAGGAGCGGTTTTCTAACGTGTCCATCCGCGTGCGTGTGAAGGGCGGTC
TCACAACTCAACTCTAAGAAGAGGGCCAAGCGGGTCAAGCGCAACACCAAGCCCCTGCGATCCAGCCTCACGCCC
ATGATCTCTCCGCCGGCCTTCTCGATGCGGGCGCGGGCACGGGCTGTCACGCGCAGGGCACACACCGTCATCTTAG
GCTGGCTGGGTGTGACGGCGCGGGCTGTGATGGCTGATAATCGCTGTGTAGGCCTAGGGGCAGCATGAGCAAGG
CCCACGAGCCGAGTGATCCACCGTTAAGAGTTGTGATTTTTGTCATTACGTCAGTCTCAATTGCCGACTTTCAAGAC

TGACGGCTGCCTCAACTATATCGAGGAGAACGACGAGGTGTTGGTGGCAGGCTTTGGCCGGAAGGGTCATGCCG

CGAGCGGGGAACAAATCTCTCGAAAAACAATGGTGACCATCCCGATGCAAGCCGTACGCGGTATCGTTGGTCATC
CCGAGGCTATATAAGCTACTGACGCTCTCCCGCTCGAGCAAAGGCAAACCAATCGCGGGAACACCAAACGAGTCTC
AAAGTGGAACTGTAGAATAAACCTAAAATAATCTGAATAACAATAATAATAATGATAATAACGATAATAATCCTAT

TATCTATAGACACGAAAATTCTATAGACGAAGGAAGTATATGGACCCGAGCCTCACTAGCCCATCTAACAAACTAAA
TATTCGATCTTTCCAGGTCTTTTTGTCGAGCTTCCAGGCCGTTTTTGATAATTCCATGTTTTTCCAGGTTAAATGGGT

CAAACTGACGATGAAGATGATAAGGAGGATTCTGCCCACCCATACCCTCATTCTGGTGACATGAAGCCAAGGTATC

ACTCCTATACAAGCTTGGTCTCAGGGCGGACTGATTCTAACACGTGTTTTTCCTACTTGGGTTTCTGTTTTCATTCCT
ATTATACTTATACCTCCCCAATAAAATATAATAACTTATTAAAACCTTTTAATAAAAATTAATTTAATTAAATTAAAGT

TTATTGCCCTTCCCTCCTTGCGACTTCTTTATCTCTTAGATGAAGTTAACAACCCCGCTCTAACATTAAAGACTATTG
AGGGAATAGTCTCGTACAGTGACCGTAACAGGTGCTGCCGCTCCTTCAGGTTATGCTGCTCTTCCATCAAGGGCTG
TAGTGGTGGTGTTTGAGCTATATAAGTTACTACTCCAATTTGATTAAAACACTAGATGGAGTGTTAAATCCATCTA

CGCAGAAAAGTATAATCCTAAGATAATAGTAGCACCTAATCTTTGAATAGCTTGGGTATGGTTTGATTCTATAATA
CTTCTCGTTTGTGCATTAACCTCGAATTAGAAGAGACGAGTTAGCCACGCTGTCTTGCATACCTTTCTCTTGGATTA
TTGACGCCCATGTCCGAGGTGAAGGTGTGACCGATTGTGAGTGAGTGAGAGAGTTGAAAGAGTGAAAATGGTGC
AGGTGGCATGCTGGCGGTGTGTTGTGGTGATGGCCAGAGGAAGGCCGAACAGGCAAGAAGAAGGAGG

AGTATTACAGTCTCACAGGACTGACAATAAGAAAAACTCAAAATATTTTCCTTCTTTATAGCTCAGCGTGAAAAACA

TCCCTCTAAGTGCTGTGTGGTAAAGCTGAAGATGGATAAAGACCGCAAAAAGATCATTGAGCGGAGAGCGGCAG

ACGCGGATTATTTCCGATTTCTATAGGACTTCAAGTGCAGCACAAGTCGATCCCCTGACACGGAGCCTCAAAGGAC
CGCTGCTGCTGCAGCCCCAGCAGCTGAAGCCAAGAAGGAGGAGAAGGAAGAATCAGAAGAGTCTGATGATGATA

GTGAATGGGCCGGCCTCTGCAAGATTGACAGAGAGGGAAAGGCTCGCAAAGTTGTGGGCTGTTCCTGTGTTGTAG
CAAACTTGATGATGTCCTTCATCTTGCTCTTCTCAAGGTCATACAGGATGGAGTCATTGACCTTCACAAGGGGGTC
GAACCGTAGCCAACATTGGGCATCAGGTACTGGCCCTTGAACCGTCGCCTTACCCTGTTGTCAATACCCTTAGGCT

AAACTCTCTTACACCTTATATAATATATATATTTTAAACCTTAAAAAATAAAAACTTAAAAAATACCTTTAAGCTTTTT
GTATTACAAGGTTGACGACAATGGCAAGATCACCCGCCAGCGACGTGAGTGTCCATCTGACGAGTGTGGTGCTGG

TCATCCAAGAGCCACCAACTGCCGCAAGAAGAAGTGTGGCCACACCTCCAACATCCGACCCAAGAAGAAGATTAA
ACCTTCTTCACCATGGTTCTTAAATGTAACGTCCTCGCCTTGTCTTCTCCCTCGCAAGGTTTGTTTACGAAAAAAAAA

CGGCATTGTTGATACTCCGCAAAGCGTCTGCTAACGCGTTCATACGCACCATCTTGTATCGCTACTCCAGGCTGGG
TGATAAATTCTTCGCTTTTTTCTTCAACAACATTCACTAATTTCCACAATTCCTTCGCTCCACCTCCCCATGAATGTCA

CAAGGTTCTGGGCAAGGGCAGCCTTCCCAAGCAGCCGGTGATCGTCAAGGCCAAGTTCTTCTCCCGCAAGGCTGA
CAGCAGGTCAAGAAGAAAGCGGGCACTTTTCTTTGGCCACCTGCCCTGGGTGATGCCCCAGGCTTTGGCCTGTGCA
GAGAGTTCCATGAAAGGTTAAAAAAAAAAAAAAAAAAAAAAAAAAAAAAAAAAAAAAAAAAAAAAA
AAAGCTGCGTGAGGACTTGGAGCGCATGAAGAAGATCCGAGCCCACCGAGGGTTGCGTCATTACTGGGGCCTGCC

AATGGCCTCTCTAGGGGTCTTGAAGCCCAGGCCAACCTTGCGCAGATGGCGCTTGGCTTTGCCGCCCTTCTCTCGG
GACTGGCTTTTCTTTTCACCCTTGACCCGTGGCATCTTGTCTGATGAGAGCGAGTCCGGAAGAGATCGCAGCGCCT

ACTATACTCTGCATATATGAATGTCCAGAATCTTTATACTAGTTAAAGGACAGAAGGTAACTCAGGATAACGCGG
GGTTCCTCTTCATCATGTGCGCGCGTTCACATTTTGCACTCATGAACACGTACACCTTGCCATCCGTCCTGACCAT
CTTTGCTTCAAGTTCAAGATCGACATTGTGTAGGTGAAACCTGCTTCACCTCAACCTTGTACGCTGGGTGGACGC
GCAACCTCTGCCACAAGTGTGTCCGTGAGAGAGTCGTGAGAGCGTTCTTGATCGAGGAACAGAAGATCGTAGTC
TTGTCAGGTAAATAACAAATAATTATATATATATGAAAATCCCTGCAAAATTCAATAAGATGTAATTGTATCGGCT
AGGAAGGAGCGTAAGAACAGGATGAAGAAGGCACGAGGCACCGCCAAGGCGAAGATGGCTGCCGCAACTAAAA
CTCAAGACGTTGAAGGGCAAGCTTGAAGGAACACCCAAGGTAGACAAGCTGCAGGCCATACAGAAGCCTCTCGT
CTTCACAATCTGGTCCTCCACATCGTCCTTGGTGAGCTTGAGCCAGTTGGGTACGGAGCGGCGGTATGGCAAGG
CTCTTATTAAATAGTAATGATACACAATAATACATCAGTGAAGGAGCCTGTGTTTATCTTAGCCAATATAGTTTTAT
TGCCCACGTCTAGTCTTGTAAGGCAACATTCCCCTGACGCAGCGTTGGAAGATCTTGGCAGGAGCGCGGTGGTG
TCCTTGCCTGCCAGGGGACGCTTGATCACATACTGGCGCACATCATCCTGTTTGCTCAAGTTGAACAGCTTGCGG
GGCAGTCTTGCGGGTCTGGCTGGACACCTTCTCAGTGAAGCCAATGCAGAACACACGCAGGATGTAGCCGTCGG
ATGGACTTGTTGGTGCGGTGATAATCCACGGCAATGCCGATGGTACGTGCATACTTGGGGTTGAGTGTTGCACC
ACCGACATGATGGGCTGCCCAATCTGCACCCTCGCCACGGTGCCCTGAGGCTTGCCAAACGCACCACGCATCCCT

GGTCAGCCATTACTGGACCCGTGGCTAAAGAGTGTGCCGATCTCTGGCCCAGGATCGCCTCTAACGCTGGTTCC
TGCCGTACTTGCCGACGATCCCCACCTTCTTGGTCCTCTTGGCCATTGTTGTCGGTTTGTTATGGTCTTGCTGAAG
CTGAAGCTTGTGGAGAAGGCTCCTAATGGCGGCCGTAGACTAACCTCCCAAGGCCACCGTGACCTTGACCGTAT

AGGCCACGCCCCATGCAGAGCCGACCCGAGGGACCACGAGGAGAGGGTTCCAGGGATGCTTACAGACGCGCTC
ACTCTTGATGACAATGGCCTTCTTGCCAGCCTGTTTGCCCGTCAACAGGATTACCACCCGACCTGGTTTGTAGACC
ACAAGAAATAAGAGGTTCAGGCGGCGTGGCATGTCGTGGCGTGGGACTGGCGACTTAGTAAAATGGAGAAGG
TGGAGATGACACAGTTGATGGGGAAATGAGCATACACAGCACGCATCTTGTACTGGAAACCCACAGTCACGCCG
GTCTTGCCGTGGTAACACTTGTGGATCAAACCCTTCTGGAAGGCTCCGTTACCCTTAAGGTCGACAATGTCCCCAA
AAAATGCAACTTTAGTGAAATATCAACATACTTTTGTTAAATGAGATAAGTTTACACTGTATTACAACGTTAATGA
GTACCCTTCCTCTTGCCAAAGCCCATGTGCCGGCCCTTCCTGCGGGCCTCCTGGTTCTTGCGCACTCTGGCACGTG
CCGAGGTGTCTGCCTTGGAGCAGGGCATCATTGAGGTTGATGCTGATACTAAGGAAATGTTGAAATTGATGGACT
GCACTGGTCTGCTGTTGGCCAGGAGGGTGTTGAAGAAGTTCAAGTTAGATGGCATTTATGAAGGCCAGAAGGAG

AACACTGGAATCTGGACCCCAACGGACTGACCCACCCACACGCCCACCGGCTCACTGATCCACGCCCGTCCCTCAT
CAAGACACCCGGACCTGGTGGACAGTCAGCCCTGCGAGCCCTGGCACGTGCAGGCATGAAGATTGGACGTATTG
AGAGAAGACTCATCAGAGCTTATTCCAGGGAGTTACAGGATTTGACAAGTCCCAAATGCGACACACAGAAACAG
CTTGAGCTGCAGCTTCTTGCCTTTACGGCCTTTACCCTTCACCCCCTTTCCTTTCTTAGCAGGCACTTGAGGCTTCTT

TCTTGGTACGCACCAGCTCGTTGTTACTGGCATTGTATACCACATCTACCACACGTGTTTTCCTGGTCTGGCCCTCA
CTCGATACCAGCCATCTGCAGCAGCTTCTTGGGTACTGAGGCGGCTACGATGCTGGTTCCTCTTGGTGCTGGGAT
TACTCTCGATGGCTTTTTTCGATTTCTTAGTAGGAGCCATTTTGTCTTTATTTCGTCACTCCGAGGATCTGAAACTGG
TATTGACCTTCACTCCCCTTCAGAGATCGTAAAACAGATCACAAGTATTAGTATTGAACCCGATGTTGATGTTGAG
ACTTAGGATGCAAAAAAAATAAGAAGAAAGAAGAGAGAAAATAAATACATTACACTCTTTTATGTCTGTGTCTGT

GCAGTACTTTCTTGGCCCCAGACGGCAGCTTGACCCTAGTCTTCTTGGTCTCAGGGTTATGTGCGATGACCTGAG
AGCCAGATGCCAATGTTCTTCACCACCTGAGGCTTGCGTTCCACCACTAGATTAATGGATACAATTTCTCCACTGG
CCTTCCTCCAGCACGCCAGTCCTCACCAGGCGGCGGAGCAGAGCGTTGCCTTGGAAGAGCCTCCTAGCATCCTTCT
TGGCCCTGCTCGTTAAGTACCTGTAAGGATGACGGTACTAGGGGAAGGGGGGGGGAGGGGAGGAGCCAATTT

AGGTCTTCATCAGTGATGTCAAGTACACGAGGAGAGAACACTGTCCCTTGGTCATACACTTGCTGAACCACAAGGCC
CTAAGCAAGCATTGTGCCGTCTCAACAATCCAATTATATTCCTGACTCACATTAACATACACCCCAGTCGTACTAGTC
GTGAAATCATCAAGCGTTTTGAAGCTAAGGGATTCCAGCTGGCCGGCATGAAATTTATGAAGGCCACAGAAGACCA
GGTCACACTTCACAGGTGTATGCCATCCGTCAGGCCATCTCAAAGTCACTCGTGGCTTACTACCAGAAGTTTGTGGA
CGGCACTGTCTGCATCCTGCTTGCTGGTATTCACAAGGGGAAGAAGGTGGTGTTCCTGAAGCAGCTGAAGAGCGG
GGCACATCATACATCCTTAGATCATCAGTGACAGTCCCCACCACACAGATGATCCTTCCCTGTCTGCCCGGCTTCTTA
CTTCGAGCAGGTCTTGGAGCACTCATCCTTGCTGGTACACTTGCATCCAGACGTACATTTGTCGCATGGGGAGCAG
CTTCCGCCATCTCTGCCTCGGGAGACGATAGTGGGAAGACGTAGTATACTGGGTTTAAATAGATAGTCACAGGTTT

GTGGGTGATATTCCCGGCATTAGGTTCAAGGTGGTGAAGGTGGCTAACGTGTCTCTCCTTGCCCTCTACAAGGAGA

CCAGCCGGGATTCTGCCTTAGAGGCTTTCAGGCATAATCCCACGGACGTAGCCTCGCTCCACTGGCCGATCGACCAA
CCTGTGAGAGGGTTCCACTCAAGTTCTTCCATTACTCTTATCCCTGAAATCGGTCAACATGCGTGAGTGCATCTCCA
CTGCAATAGTATAGATAATGATGATAGCAATAATAAAAATTGTAAAAGTAATGATGATAATGATAATAATTCCGTA

AACACCAGGAATATGTTGAATCCAATTATTTATTCTCACGAGATAAAGAGTAACACTGAAATCAGAATAAGTGTTAC
TGTTGGAAGTACATCGGTTCCTGGAAATTAGTCCGTATTCCAGCATCTCGATCTTAATTTTTTTCTGGTTTTTTGGAA

CTTAGTACAGATGCCCTCGATGCTTTGGGTGGAATAGCTGTTTCAGTTTTGGGTATTTTGGCGGAAAACGGAAAAG

TCACTCTAGCTACGGAAGGTGCTGTTAACAATGTTCCTACCTGTGATTATACTGCTGGTACTGCTGACGGCGACGCA
TTAAAATATAAAGCTTTATAGGGTCTTATCGTCCCCTTGAATGATTTAAGCCTTTTCACTTAAAAGTTAAATTCTATTT

GCCACCAATGGTATTGAAGGTATGAGTATTCAGATTTTCTACAAGTAGAGTTTGACTCATATATACTTCCTTTTAATG
GAGCAACACCTCGCACCATGCACGGACTGACCTAACTCGGACTGGTGTTAAGTGTGTGTTAGTTAAACTAATCACCT
GTCAAACCATCCTATCTTGAGCTGTTAGGTGTGACTGGGGGATTCTCGGCATTGTTCACTGATAACAGGCGAAATG

GCGTGGTGTGCTCAGGTTACAGTAGCCCCAGAAGATAAAAGAATAGTAGTATTAAGAAGGGGAATCTGGAAGGG
ATCTGGCTTTGTTATTATTTCACAATGCACCGGAGTAGTGATCTGCAACACGTGTCAAATAACTAAGTCACTCGCTGT

ACGGACACAACAAAAAAATTCGGTAAACAATACATGTGTTACTTAGTGTTACAGACAAGAGTGATAACAGGAAAAT

GTCGTGCTGCTCGCCAGGGCAAGGACAAAGAAAAGTTCACTGCTATGGATACTTCTGCGTAAAATAAAAATCTTCG

ATGGGCTTCAGTCTGTTCGGCTAGACGTCAACACACACACACCACTCCCAGCTTAACCTGACCAAATAAAGCTGATA

GTCACAGATTATGGCAAGGAGACCCAGGCACATGATGTTGTGAATGACTACTTCAAGAGCAAGGGCCAGTAAAGA
TGGATAACGTACGGTTCTGCCATCAGAGGTCACAAGGAATGGCACTCCCTTGGGTCCAACAGACACCTTCTTCACC
TCCTCCAGTTGGGCCTTATCTTAACATAGCGGTCACTCTGGTGTCGTATGAACTTCTTAACTCTTTTCTTGACAATCT

TATCGTCGTTTAAACCTACTTTTTCTCCCACATTAAATTTTTATTTTCTATTTTATTCTCACTTTATTTTGAAAGTTATTT
GTGTCTTCATGGCCAACATGTTTGACCGTCAGTACTGTGGCCGATGCCACCTGACTTACGTCTTCAACAAGCCTGAAT

GGGTTAAGTGTTGGTGTTTCTGTAACAAGTTGAGACACTGGAGAATAGGTAAAGCAACATACATAATACTTCAATA

AAAATTTCAGGGCATGTGCTACATCCACGTTTATTCACATTTTTATATTCATATGGAATACAATATATGATCTACACAA

GGAGAAGATCAAGTCTGTTGGCGGCTGCTGTGTCCTTGTGGCCTAGTTGACGTGAGGCTGGTCTCCATCCGTAGCA
ACACCTGCCCACACCCCCATTGTATACACGGAATGGTACACACTCCTTCTTGGCAATGACGTTTTTCAGGTAGCGCA

GTGTGAGGGGACAGCACACTAAGACCACCGGCCGCCGTGGTCGCACTGTGGGTGTGTCCAAGAAGAAGTAAACAT

GATAGCCTTGGAGTTAAGGAAGACAGTAGGCTGCTTCTGGAAAGCCTTCTCCGTCTGATCCATGATGGCAGTCCGC

CTTCATTATAAATGAATTTATTCCTTTCTAAATAACATAATCCTTATCAAATAACGTAAGCCTTAGCCTAATAGAATGT
AGTCTTGCCATGACCAGGCTGGATCTTATATCCGCTGAAATTACACAGCTGGAGCTTCATCACGAAGATACAGACTC
CGCTGCCACACTTCACCTTGCAGCTGTACCATTATGCGGTGCTTCAGAATATGTTGAGGAAGATACAGATGATCCAA
AAGGTTCTCAAGGCCCAGAAAGCTGCAGCAAAGGCAAAGTAAAGGTTTAATAAAAAAAAAAAACTAGTTAAAAAA
TCCGTCTACAATCTATGTAATAGTTACTTTATGTTGGGCATCAAAAACCATGGCAAACAGAATCTGATCTCCCATTTT
AAGAAGTAAATGGAGCAACGTGGATAATAAACCTACAAATTAAATCAAATATTGTTTGATTTTTCCATATTTTGAAG
GGAGCTGCTCAAGAAGTTTAAGGACCTGCAGTTCTTCACCGGGGAGTCCATGAACCCCGATGGCATGGTGGTGAT
CAGACTGGGATATACCCTTCCCTCCACTGTGCATACGACCCATTTTTGTGAGTTTCTAGAGAGGTCTCGGCAGTACA
TCAACATTGCACCTGCAATTATGATATGAAAATGGCACGTGCTCTATATTTCTATGTATACTTGTATTTTATTCTTGTT
GAATGGGCCTCTCTTGGGGTTGACATTGCACCGTTTACGCAGGAAGGACAGGTACTTCAGCTTGTTGCGGTAAAAC
ATCTTGGAGGCACGCTTGGGACCCAGGCGGCGAGGCACAGAGCCGTCAGTCAGGCCAGGGATGTCCCCATCACCC
TGGTCTTGACGTCGGCGTTGGCTTCGATGCAGGTCTGCCATTTCTTGACCATGGAACGCAGCTTGTCGGTAGTGAG
CTTAAGTTCTTCGAGGGTGAAGCCCTTGCCCAGCCGCTGCTTGGTGTTGTACCTGAAGGTGGGGCAGCGCACAATG
GTCTGCAGCCGATCAGCGCCGGCACACGACAACATCTTATTAATGCGGATCACGTGGAAGGGGTGGAGCCGGACA

ATCGCCTAAGTAGAAGAATAAAAAAAAAAAAAAAAGTTAAAAAAAAAAAATTTACGGAAGTGGTGGGTGTGGGGA

TGCTGCACAGATGAAGGCTGCCAAGGCCAAGGCAACACCAACTGTACTGACCACCACTCAGTAGAAATAAATAGAC

CCAGTGGCATGGACAAGACTGGCGATGCTGGTGCTGGCTCATCCACTATGGAATTCCGTGGCGGGTTTGGACGGG
CTTCACCATGTTTGCAGTATGGTGGAGGGAGGGAAGGATCGGAAGAGCACACGTCTGAACTCCAGTCACGAACCG
AAGGCAAGGCAAGGCAAGGCAAGGCAAGGCAAGGCAAGGCAAGGCAAGGCAAGGCAAGGCAAGGCAAGGCAA
GTCACCATGTTGGAGATGTGGGAGCACACGGTACGCACAGCTGCCACCTCCTTGCGGTTACCGAACCATTTGTTGA
CCTTGTACACACGCAGGAAGGTGGACAAGTGTTCTGTGCCGTTGGTCTTGAAGGCACGGGAGAACATGTTGCGCG
GGCAACCTTACGTCATACGATATACCGTTACGCTGTATCATACAACTTGACACTACAGAATACAACGCCAAGCTACT
AGTGTACATTGTGGGGCTTCTTGATGATGAGGCCATCCTTGATCAGCTTGCGGATGTTCTGGCGTGAGTTGGCTTG
TCGATAAGGTTGCAGGTCTCCAGGTGACCCAGCCAATCCCATCCACTTATGGCAATAGGAGATAAATGGTGTGGT
GTGGATGGAGAGATGTATACAGTGGAGGATGTGGACAGTGGACCTGGAGCTTTCCGGGCCAACCTTGACATAGG

CACGCTTCTGTTACTGCTACTACTGCTGCCACTGTTACTACTGCTATTTTCACGTTCGGTATATCTGTTATAATGAATC
AGGATGTGACACCGGTACCCTCAGACAGCACACGTAGGAAGGGTGGCCGCAGGGGCAGGCGCTTGTAAACAGTC
AGGAAAAGGTTGCCCTGCCTGTCAAAGAAGATATTGATGCCGAGAAGGGTCAGCAGGCCCTCCGTGATGGTATTG
GCTACTGCCCTTGGCCTGGGGCCGCTTCGCTGCAAGTTTAGCCGCCGTCATATCTGCTCCGCGTCGCTGAGGGAAA

AGAGCCCCAAGCAAAGTTTCCAGTGTCAAGGCGGAGAGCACGGTACTTCTTGTTGCCTCCACGGGTCCTTACGGTAT
GAGCCGCACCAGGACAGAACCACACTTGCCGGTCACCTTGCATGGGACAGTGTGAGGCTTTCCGATCTTGTTGCCC

GTGACCATCGCTGAGTGAGGTTGAAATTGATGGTGATTAGAGTGGCGGTGTTGTTTATGTTAATGGTCTCCGAGG
ATTGATGTACGTACACGCAACGCACACACACACACACACGCGCACATAGCATCTGTGTGTGCGTATATGTGTATGT

CGTAATTACCAGAGCCGCGAGCAATGCGTCCGCGGTCACCTGTCTTCTCCTCCAGATTACAGATGACGGTGCCTTCC
ACTTCTTCATCTTCTTCAGCTTGGAGAGGAAATACCAGAATCGAGACTTGGCAGTGACTCGGTCTGGTGCGAAGATC
TCGTCTAAGGTGAGCAGTTCTCGGGCAGCTTTACGGATCTTAGCGAGGAACATCTTCACAATCCACAGCTCCCTCTT
GCAGTAACAAAAATACTGTTCACAAATACTACCTTATATAGCACTTTCCTTTTCATCAGTAGTAGAATTTATTTATCTT

CATAAGTGAACGGGGAGATATTCAGCATGTTGAGAAGTGTGGCCTCGCTGGCACCCACTTTGTCTCCTTCCTTCAC
CTCAAAGGGAATATTATAAGACATTACACCTGTTAACACATACACACCATTGTAGCCATCCCAGCAGAGCAGAGGA
ATCTCAAGAAGCATTATGCCGACCTTGCTGACAAGCCTTTCTACGCTGGCCTCTGCAAGTACATGGCATCTGGACC
ATGAGGCTTCCAAGAAAGAGATCAAGAACATTCTCATCAACTATGACCGTTCACTCTTGGTAGCTGACCCGAGGCG
CCTTTGTCTTGTGACTGGTCCCTTCAAGATCAATGCTTGTCCTCTTCGCCGCATCAACCAGATTTACCTGATCGCCAC
AGCAAGGCGGGTCAGGCTGGACAGTGACAGGGGAGGCCGGTGGATGCGGGACATAAACAGACGCTTCAAGATCA
GCAGCAAGCAGTGCACTTGCACCCTGCCTTGCACCCGCCCTCCTTGCACTCACACTTGTCTTTGCAGCATGGATCAG
TGTGAGAAGTACCTCGTTACCAGCCGGTGACACCTCCCGACGACGTTAAGTGACCGCTATCATTCGGAAACTTTGC

AGAAGGAAAGGCCTCGCTCTTAAATGTCAAGATAGAATAAAAAAAATATATAAAAAACAAAAAAAAAAAAAAAA

AGAGCGGAACCATATGTCCGGACCTGCCGTTCCTCTCGTACTGAGCAGGACTGCTGTATCAACGACATAACTGCCA
ATCCATGTGGGTCAGGCTGGCGTGCAGATGGGCAATGCCTGCTGGGAGCTGTATTGTCTTGAGCATGGTATTGCGG
AGATTCAATAAAGATAAAAACTCATGAATTGACTAAAAAAATCAAAGAAACTTTTGAAACGAAAAATAGAGTTTATT

GTGCTGTGGTGAGAGTCGATGTTGAACCTCGCTGTGTGCTGTGTGGTGTGTGCTGACTGCTGTGCTGAGTCAACCA
ATAGAATTTTGGGTGGAAGTTAATTTGCGTTCCAGGTTTAAGATTTGGCTGATAGATTAATTGTGTTCCAGTTGTTC

GACAAGCAGAAATGAGGACTATAAGATCATCATAACGGCCAGAATGGAAACAGAATAAAGTCTAAATGCGCATCA

AGACGAAGGGTAACGGTTCCAGTTTCTCTGTGGACTGTGCTAACGTGCAGAGATCCATGTCCTACGTGTGTGCGAC
TCTACGTCAGAGACAGCTCTTCTTTTGTCCAACCCTTCATACGAGTTCCTAATTAAGAAACTAATGATTATGCTACCT

GAGTTAAACGAATCAAGATTTCGACTCTTAGATGTAGATAACCGAACTGTTTTACCAATAAATACTCAAATTCGAAT
TTAACAGTATTGAATTAGAACGCTTGATTTGTCTTATTGTAAGTTCTTTTCTTCCTTTAGAAAATTGCTTCTTTGTTTTC
GACCTCTCTCCCTGACAACACCGCGGACCCCACCAGCCACACCCGGAAGCTCAAGATAGACTGATTTGACTGTAGAT

GGTTAAAAGGCTGGATTCCTAAAGGTGGTCAATATATTCCAACTTCAATAGTAGGTGCTAATCTTCTATGGAAGAAG
TCGGTAAAACATCAATCCAACATACGTGATCACAAATATATAAAGTGTGTAGAAGGAACACCACGTTTGTTGGCTG

TGACAAAGACTGTGTTGGGTGAGAGAGGATGAATGGTACCGGGAAAAGGACCGGTCACGGGAGAATGACTGCAT

AACCACTGCTTTCAGGTGCACATTCAAGGCGTTAACAGTGGCTGGAGACCTGCCATGAAGTCCTGCTGTGCCTGAC
CTTGCACAGCTTGTACTTAGCCTCTTCATCAGTGATACGGTGAGCAATGAAGCGGCCCTTTACATCATACAGAATGC
TGGGTTTCTTCAGCGGCCGGATCGCCATGGTGCCTTCTGTTAGTCCCCACGAGGGAGAAGAAAAGAGG

TAATTTTATCTTTTATATCATCTTACCTTACACATTAATTCTTAAGTTTTAATAGTTTAAAAAAATAAAAACTTTGGTTT

AGTCTGCAACATAATAATGGATTCTCCCACCCTCACGCCTTCCCACCCAACATTTATACACGCTTGCACAAAACTATG

AAGTCATCAGGTTACATTGTACCGTTTGATAATAAAGAGAGATTGAGAACTAAAAAAAAAAAAAAGATCG
ACTGATCGTGCCAGTGTCATTTTCTTGATGGCATTGGCCGTCTCACAGGTGTTCTTGAAGCTGCATCGTAGGTTTGAG

TCTGAGCTCCGAGATCGGAAGAGCACACGTCTGAACTCCAGTCACGAACCGCGATCTCGTATGCCGTCGTCTGCT

TAAAAATTGACATCCTAAATCATGTACATTTAAGCTAAATGAAGGATAGCAATTTAGAAGCACTTCCTGTGGACAA

TGATCCTTAGAACACATTAAAACACGCTTTTCATGGCGATCAACAATCACTTGCCACCATCACCGTCAGACCGATG

TTATTGTAATCACCATAATATAATGTAAATAACCTTAATATTATATAACCATGGTATATGGATGATGATGCAATGTG
ATACATTTAAAATGTTTTGATTATTATTGTATGAAATATGTTTGAAAATGGCTGTTATGTTATTTTACTACGATAAG
TGGGGACTACAAGGACATCGATGGGGAGGAGCGGCCAGTGTTGTACTTCCCTAAATATGGTCTACAGGAGGAGA

TTGCAGTAAAACAACGGAATTAATTATAATAATAATCTTCCTAATGAATTGTGTTTTTAACACTAATGCATTATTGC
CGAGCCGGAGATGTTGATTTTCTCGCATCTGACCACCACCACTCGCTGACCTGCCAACGTGGTCTTGGCAACAATG
TTCTTGATGATGACCAGAGCCAGCACTGACAGGTTGGAGTCCACAATGCAGCCACGCACAGACTTCCTCTTGCGC
GTTGAACCCGTGAAAGTTACACAGCACGTTCTTGCCCTGTACCTCCTCGGCGATCAGACGGAACTTACGAAAGCT
GGCCTCAGCTTGCCAAGAGGCCTTGGGGCCACACACCTGGCCTTAGCCTGCCTCTTGTTGCGCCTCCGCTGCTTA
CGCACGTGGAACTGGTCCTTGCCACAGTTCTTCACCAAGTACTTATTGGCACAGATGCGCCCAGCCTCCAGAGCC

GTTCTAAGAGGCCACAGTGAAGTGTTAATAAAAAGAAAATTTGAAAAAAAAAAAAAAAAAAAAAAAAAGATCGGA

CTGTTACTATGGTGCCCTTCTTCACCTTCTTGGTCACAATGTCTAGTTTCATGTCCTTGAATGAGCGCCTGAGTGTG
TTCCGCGCCTTAAACCCTTTGAGTTAGTCATGTTGGCGGTTTATCTGCACCACACACACGAGGATGATGGAGGAA
CACAATACAGCGTGACATACATTAACACTACACAACATGCAACGCTACACACTAACGCACAACACTATCTTAAATC
GCCGATCTCCCCGATTTCATTAGGGTCGAGCCACACCTTCTTCTTGCCACACTTGAGCACCTCTGAGGCGAGGCGC

CCTAGCTCGCACTACCACTGGCGCTCGCATCTTTGGTGTGATGAAGGGTGCTGTGGATGGAGGCATTGAGGTGC

GTTTGTGGTGTTGAAATGGATTTAAAAAGTGTTCGTTATTCTTCCGTGCTGCCTGCTTTGTTTATCAATGTTTTTTTT

AGAAGTTTGACCCCTCAGGTCTTAAGAAGACCGAGACCACTGAGAAGAATGTTCTTCCGACTAAGGAAGTCATTG

TGGATCCTCTGGGGGGCAAGCTTTGTGTTAGCGGCAGGGCGACCAAGCTCAAACTTCCTCTTCTTGCGAGGTTGC
CAGTAGCCTCGCCTCACAGGAACGATAGACAGCTTGGCCAGAATGATAGCACCGCGGATAGCAGTGGCAACCTC

TGCGTTAATTGATGTATTTGAGGAGGTGTTAATATATACTTGTCGCGCTCTTGAAAAAAAAAAAAAAA

GGGAGTGTTCCGATCGGCATGACATTACCCACGTCCAGGTTAGCCTTCTTGCCACAGTACACAAATTGTCCAGAG
CCTCATCTTGTACAGCTGAGGCTCGGGATCAGTGTCGGACGGCAGCTTCCGTCCTATCACGCTGTACTCCTTCAGC
GTTACGCAGCCCATACTCACCCACAAGCTTTAGCTCCTGGTCGAGACGTGGCTTCTCAAACGGCCTCCTTGGGGTG
TTAAAAATATTCAATACAAATAACGATTCATCACCTATGGACAACCTTACATTGTTTTTCCATTGTTATAGTTTACAA

CAGCTGCACATCATTCACAATTTCAATGGTACCCCTGGAGATCTTGGTTGGGATCTGCAAAGCCTGGAAGAAACTA
GAACCACCACTCCTATAAGACATTTTCGAGCTAGTGCAGAGAGAGGAAGGATGATTAAGACTGTCTCCGTCCACTT
CCTAGTAGCCATGTGCTGGGAGGGCACCGGTGTGGTGAAGACTGCACGCACCATGATGGGAGAAACCAAACCTG
GTGTGAACCCAAGAAGTTCGGTGGACCAGGAGCCCGCGCACGCTACCAAAAGTCTTACCGATAACTCCATCATTA
CGGCCACCAAGCTGGACATTGCTGCCTTTGGCGTCCCCAAACACATCAATGATAAGTACTTCAGGAGGATTAAGGC
ACCTGGTTGAATCTGGAGTAGGTACGGCGCGCTAGGAACCGGTACAGCTTGACCAGCAGACGAAGGTAAACATCC
GCATGGTAGTGGTAGTTGAGTTGAGTTGAGTTGAGGGAGAATGGACTGGCTTGCTTTCCTCTCAGTGGAGTGTGC
CCGATAAAAGTCGGCCAACCTAGCGTACGTCGGTTGGTTGGAGTACCGGTGGTGTGAGTAATGGTCTACCTCTGTC

AAAAACCAACTCCAATATAAACAAACAAAAACAAATAAAAAACAAAACACATAAAAAAAAAAACACAAAAAAAAA

ACAGTAGGGTAAAACTAACCTGTCTCGCGACGGTCTAAACCCAGCTCACGTTCCCTTTTGATGGGTGAACAATCCA
CCTGACGGCTCCATACCCTCCGACAAGGCCCTTGGCGTCGCTGACGACTCCTTCACCACCTTCTTCAGCGAGACCGG
TTTCTGGATGCTCACAAGATGAACCAAATAAATCCATATCAAGGGATGTTATTTTCCATCTCCAAAGAGAGGTAGTC

AAACAACGAAAAATAAAAACTCAGTAGGGGAAGGCGGGAGACTGACCACAGATTTATATTTTACTCCTGTGGGGC
GGTCTTCAGGCTGGAAAATGGCTTCCATTCCAGGCAGTTTTGATAAATTGCATCCGCATTCACTTCCAGTTGGTGTT

CATTAAACATCTCTTCTGATTGAAAGCCTCACGCACTCTAAGATTAATAACTTGAGAACAGTTGTATAGGTTTTTGTA

CCTTCAAACTGCCCTCCAACGACAGAAAACCTAGAGACACAAGATTACAAGACCCAGCCACTTCTGACACTACCAAC
TTTGCACGGTCAGAATACCGCGGCTCTTTAACTTCTTGTCAGTGAGCAGGCTAGACTATTTATTATCTCAAATAGACA

TTATTATCTCTGCTGCCGATGTGATCCATTCTTGGACAGTTCCTGCCTTAGGTGTAAAAGCAGATGCTATCCCTGGCC
GTTCGTTCTTATAAGTTAATGCTTTTTCGAGACTTTTCTTTTGACCTTTTTGTTTGCATTGTTGACGTTTTACTGAGGT
TGAGCCAAGGTTTTGTGCTTTTATGCAAACTCCTTGTCAAACATGAACTCTCCCAGGCCGGAGGTTCCAGCACGCTT

GGCTCAAAAGAAGGAGAAGAAAAAAAGTACTTCTGAAAGGATAAACAGGATTATACCTCATCGTAACCCTTTAATT
GCAGCGTGGTGTTGAGGTCATCCCAGTAAACACACGTGCTCACATCGCACCACCACCACACACATAATCACTTGTCT

TACAACAGATGATTTCCGTATAGCGCCTCTTTGGTTTATAATTTGTAAGTCCAATCTATTCAAACAAGGGCTGGGCA

CTGGGCCCCCTTCCATTATTAAAAAAAAAAAATTGGAAAAAAAAAAAAAAAAAAAAAAAAAAAAAAAATTATTTTAATA
GGAAGTGCTCCCCAGTCTTCTTGATGCTAACCACATCCATGAAGCCAGTAGGGAAGTTGCGATCAGTGCGGATCTT

TTGTAAACCAAAAATGAAATCCAACTTCTTAAAACTTCAAGAAACTAATCACCTTGATATGACTTTATTAGCTCTTCCT

GCACATCAACCGTAAAATTTGTTACCCTTGAACACTGGCGTTGAACAGAGCACGGAGAAGGTACAACTCAATAGAT

CCCCTTCGCCTTACAGGACTTGGTGGTATTCTCTGGTTGGGCACTGTACCGCACCATCTTGTTTCTCGGGGCCGGAC

TGGATGGGCCAGACTCGTCATATTCCTGCTTGCTGATCCACATCTGTTGGAAGGTGGAGAGGGAGGCCAGGATGG

GCGTCATTCTCCCTAAAGGGGGATAGTGACAATTTTTTCTTATAAGATTTAATGTAAAGTAAACTCAAAGCCGAAAA

ATTACAGTTTGTCCAGGTAATTGGTCAAATCGGGCAAACCTTCTTTCAAGCCCTTCCTCTTCCTGGTGTCAACAACA
AAGGAAGATATGATGTGGATTTGTATGTAATAGTTTTTATTTATTCTTATTTTTATAAACATTGGCGCACAATATAGA
AACTATGAGCCAATATCATTATTTATAGATATAATTGCCCTTTAACACCAGGATAACCATTGGATCATCTGTTACCA

AGAAAATGGTATTTCTCAATTTGTTTTCAAAACACATGTATGCAACCCTGCCTCGAAAAAATAAGATATATAATAAC
GCAGCCAGGCGACCAAGCAGGTGCCCGGCGCCATCTATCAATATTGGCTTTGCCGTGAAGCCAGGCATGTTGAGG
TCACCAGTGCGGCGCGGACGGTAGCACGAGTGACCCTTGGAAAGCAGGAGTCGCACACGGTTGTTGGTGAGGAC
CCTCTCAGCATCAGTCTCGTTCTGGAGGTCAGCCAGGGACACCTCGAACACACGACCCTTCAAACCCTCGGACGCGA
CGTGCCGGCTGGTTGAACCACGTCCGGACATAGCGCTGCCAGTCCTTGTGGAAGTGGCAGTTGGGGATGATATTAT
TCGGATGACAGCTGTTCATATTCATCCGACACAAGATGTACACACAGGGGCATCTCGCGCACATCAGCCTTCTTCCT

AGAGCGTCGGGTAGGGAAAGAGTGTTATGGTGAGGGTTTTTGGGGGGGGGGGGGGTGGGGGGTTGTTGGG[illegible]

CCTCGGGGGCCCTTCACTATCACCACCCGCTTCCCCACCTTCACCGAAACATTCTTGGGGATGGTGATCGCCTGGGA

ATACTACACATGCAATACAACTACAATCAGATATCCACCTCTAACAACCATTAAAGGAAACAATACAACTAAAAGCT
TTCTGGACCTTGAGCGAACTCATCTTGGAGTTGCGGGAAGGGAGAGGAGGAATTGGCGAAGCCAAC

CCCACAGTGAGAAGAGGTTCCCAGGCTATGACAAGGAGGCCAAGGAATTTAATGCCACAGTCTTACGTGACCACAT

TTTTTTATTATTATTGTTATTATTTCCTTTATTTATTTCATGTTTATCACAGGGCATTATTCTTTCCTTTTTTTTTGCCTT[illegible]

AACAGGAGAAGAAAGCCTAAATCGGTGGAAGTGGCCTCAAGAGTTACTCCTCCTGTCTCCTCCCTGCTCCCTCAGC

ATGCGCTTGCCCCCAGTGGAGCGCCGCTTATGCCAGTGGTCGCGTGAGATACCCATGGCGGAGAGGTGGGGAAG
CTTGCTACACTTCACCCCAAGCCCGATATGACCATCAAAGTCACCGATAGCAACAAAGGCCTTGAATCGAGTCCTCT

TACATTCCTTCAGCGGCCAGGAAGAGTTCCTTACGCGTCTTGTACCTGTACGGGTCACGGAAGTGCACCTCGGCTA
AAGCCAGACGCCTTCATGATGGTGGTGCACACGGGAAGGAAATTTTGGAACACAACAACCTCCTCCTCTCCTTGTG
GAGTACCGCTTGCTTTGGACCAGTTGTACCCTGTGGTTCACCATCGTGAATGTATGCTTGTAGACACCAAGGGAAC
TATCCGTAGTCTCGAAGCAGCTGTGGTTCAAAACTTTCATTCCGAGCCACTAGTGCTACTCCTGCTGTCCTCCACGC

GTCTTCTCAGGACCAAGACCAGTGTTCTGGTTGGGAATGGTCACAGGGCATGGAGCAAGGGCTCCAGCACGAGC
TGAATGGAGTTTAAATGCGTCTGACAGCTATCGTAATCCCATACCGGTGTATCGAACTTATTTACCTTATTACTTTCC
CCGACTCTAAGCCTGGCACCATTAGAGGAGATTTCTGCATCGAAGTTGGAAGAAACATCATCCACGGTTCAGATTC

CAAGCGTCCCAAGAAGGAGGAAGGAGACATCTTTGAACAGAAGAAGGAGAAATATGTGCCGTCAGCCCAGCGC
TGCGACTTGGGTTCACGTCGCTTCACTTTCCGATCATGTTTATGCGAAATATCCTTCCCCATGGCGGCAGCGGGAG
AGGATGACTGACTGCCTTCCCATTGCATGACGCTCTTTTATGGGCCGCGCTGGTGCTGGTGTTGTCTTGGCTGGCC
CACAATGGACAGAAGAGAAGAGTGTGGGTTGAGCTGCGGCACTGTTTGCCTGTTCTCAGGCCGGGCCGCCAAA

ACGCTTGGTGAATTCTGCTTCACAATGATAGGAAGAGCCGACATCGAAGGATCAAAAAGCGACGTCGCTATGAA
CGCCGGCAAGCACGTGCCCAGGGCGGTCTTCGTCGACCTCGAACCTTCAGTAATCGATGAGGTGCGCACCGGAA
TATTTCAAGCGACAGAAGCATGGAACAAGGCAGACACGTGTTGACAGACGCAGACCATTACTATGATGGCCTGT

GTCTATAGAGGGTTACCAAAGGGGTCATAGTGGGAAACTGTTTGGATGTATTCGCTCCAAACCTTATGTATAATTT
TGGTCTTTCAGCATTCCAGATTGTTGATATTCCTGGAAATTAGTTGGTATTCCAGAATCTATATCTTTTTTTCCAGA

ATACAGTTGCTAGACATTACAAAAACATTAACGATAATACCTACACAAACTTGGACTTGGCACGGCAGTATTGTTT

GTCTACCTTCCATCCCCGCCCGCCTCACGCAATACCCATGACACCAGATGAAGGGCTGACGCTGACACCAATGCTTG
ATGTTTTTGATAAACAGGCAAAGAAACTTGTTTGCCGAATTCCTTTAACATAACTTTTTTTATTTATAAATTTAAACCT

CGATTAAACCAAACTAGGGTGTTAATTAACCGGCCTGGTTTATTTTTTGGTCAGTGTTCAGAAATTTGCGGAGCTAA
TAATGCTGACTGTTAGTTAGTCTTAGATGCTCGTGTTAAACTGACTCTTTGTAACCATATCTTTTTGAAATAAATGAT
TCAGGCGGGTGATCATGTCCCCAATCTTCTTGATGGCTTCCACCTGTTCTTGAAGATACTCGTCTTCCAAGAACTTGC

TACTATAAAAGTGTGGAGACCTTGGTAAGTTCCTTCTCGAGTAATATCTCGTCACCATTGAACTATGGTTAATAGAAT
CACATCAAACATATACATTACCTGATAAAATATTGTCAGAACTTTATTCGTTACATTTCTATTGTTGATTATTTTTTCT

ATCCTCCTGCGATATGAGACCTGCTTTTACTGCTTTCTCGGCAAAGTCTGCGACAGCCTTTAGCACCACCCTTGTTCTC

AAAAAAGAATATCAAACAAAGAAAAAAAAAAAAACAAAACACAAAAAACCCAAAAAACAAAAAAAAACGAACAACC
TGCCATCCACCCTGATGAGCCTCTGCATGGTGATCTTCATAACTTCAGTGTTGGTAAGGGCATACTTGAGGCGGTTA

TATTATATTAATCTTATCTTTCCTTTTTACTCGGATATCCCACCCCCTTTCTATAGGATTAATTTTATTAACTCAAACAA

TAAATAGTTAAGTCCTACAAACCGCAATACATGCACACTACACTACAAATAATGAAGTCACGTACCCTTCAGAGAGG

AGCCGCCGATCCAGACGGAGTACTTGCGCTCTGGGGGAGCAATGATCTTGATCTTCATGGTGGAGGGCGCGAG

ATATTTCAATTAAATACAAATAAAGGAACGGATGAGGTTTCTTGATGACGATATGACAAAATTCCGGAAACTAAC

ATCTGGTAGGGCTTGCTGTTGCCAGTGATATCCATGGGATTGCCGGGCATCTCTTGCCAGTGGTCAAAGACACAC

TTACAATCACTGGATTTAATATGCGCCAATAAAATTGAAATACCCTCAAAAAAAAAAAAAAAAAAAAAAAAAAAA

AGAGCATGAATTTTATTTACTGAATTCTTGGATGAAAGTCATGTGTGTCTTATGAAGGTATATTCAGTAAATGTTG
AGATCGGAAGAGCACACGTCTGAACTCCAGTCACGAACCGCGATCTCGTATGCCGTCTTCTGCGTGAAAAAGGG
GCCCTGCTTCATGGGGAAGCCCTGCTTGTCATTGCCACCAGCAATGCGCAGCACGTAGCCCTTCCACTCATCACC
ATCTTGGTTCCTTGTGTACGGTTCACCAGGGTCTTTCCGACATTCCTGTTGAAGAAGAGTGCTGGGGACTTCACAT

CCCAAGGTCAAAAATACGGATCTTAGCGTCAGGCACACCCCTGCAGAACCGACTCTTGGGGTACGGCTTGTTCTT

TAAACACCTCTAATGGCAACTGTTATCTATTCAACTACAATCAAAACATTACGCTAAAATAAGATGAAATATTAAAA

TCATGGGCAAGCATGTAGCCAATTACATGCGAGAACTGATGGAAGAAGATGAAGATGCATACAAGAGGCAGTTC

CCTTCTTTCCTTCTTTCCTTCCTTCCTTCTTTCCTTCCTTCCTTCCTTTCTTCCTTTCTGTCCTCGCATGGTTTTCAAAAT

CCTTCCTTTTAGGGTCTGCAAGCCAAAATGTTTTGTACTTTGATAACCATAAGTATATAGGTCAACTGATTTTGTA

TGCCCAGCGCGTGTCTGCTTCTGGACAGGCATGATCTTCAGCACCTCATCCTTCAGTTTGGGCCCAAGGAAGAAGT

TGGGGGCACCGCGGCCTGGGTCATGGATGATGGTCTTAATGATGCCACGGATATAACCATGTCTCTCAGCGTAA

GGCACACGTGAGGAAAATATGGAGGAGCCTCGTCACAACCTCCGCCTCACTCACCTTCTCTGATCTGAAAAAAA
CCCTGGGACAACTTAGGGATCTAGGAGCGTAACAAGGGGCGAGACACACTTCCTGAGGTCCTATTTTCATGATGG

AGGGGCCTTCTTCTTGTTGGCCAGCAGCTTGTCACGCACATCCACCAAGTCTTCATTGGTGAAAACGAACCCCACG
TCCCATTCGACAGCCACACACCACCCAGGGCCAATCACGTACCCTAGACAAACAGGTAACACGTATGGGGTTAAT
AGTAGAATCTGCCAACAAGGAGATTGCCTTGTGGTTCAAACCCGAGGAACTGGTCAGCTGGACCCAGGCCAACG

AAGAAGGACCAGGCAGTCGTGGACGGCATGTTGATGTCTGTGATCCGCAAGCGTCAGGACAAGAAGATGCTCTT

GTGTGCTGTGTCGTGTGCAGGACGGATACCGCGTCGTCTCCATGCACTAGAGGAATGACGTAAGATTGCTTAGG
ATTGATGCATCGGTCAATTTTTTTTTTTTTTGGTCGGTACCCGGCCACCCGAATTTTTTGGTTTTTGTTGCTGCAAG

GCTTGGCCGCCACAAGCCAGTTATCCCTGTGGTAACTTTTCTGACACCTCTTGCTTAAAACTCATAAAGTCAAAAG
CCTACCGCCAGCTGTTCCACCCCGAGCAGCTCATCAGCGGCAAGGAAGATGCCGCCAACAACTACGCCAGAGGAC
GACACAAAGGTTCAAGCATACAGCTAAGTAAACGCAGCCCAATTTAGAGACCTTTTAGGAGAATGTAGTTAAGTT

CTCGGTGATAAAAGAGGCTCCTGTCCACCACGTAGAAGGTTATGTCACGATTACCGGAGAATTTGGTCACAGTCT
TAGAATTCTGGCTGGAAGTTGATCTGTGTTCCAGGTTTGAGATCAGGTTGATAGATTACTTGGGTTGGAGTTTTC

TCGATTGTTTCACTAATGTTCATGTACTGTACCTCAACGATCCAGAATAAATGTTGTCTGGCTTTGCAACGGGAAT

GCCTACAACCTTGTCAAGACCCAGTGGAGTGGAGGAAGGTTCCGTAGAAGTCAACGGTCTGTCAATGCTTATAGCC
TTATTTTATTTTAGGGCTTTAATATACAAATAAACTTATACTAATATAACCATTAAACCCCTATAAAAAAAAATTCTAT

ACCATAGATTTATACCAATTGTAATCGAGAGAGTCTCTACAAATTCTTTCCTTAATTGAGTTTCTTCTCACTCGTCTGA

CCCATATGGGGATCATTATTCTTGCCAGCAGTGACATGAAGTTCCAACAGGCTCGTATTGACTTGTTTTTCCAAATCA

TGGCTACAAGTCTTAAGAGGAGTAAACTTATATTATATTGGTGAAATCATTTAATTAACCCGGTAGTTAGTATTATT
TCTTGCTTCTTTGCTTTCTACATGTCTCGCCTGTTGGAGGTTACATTAGTATTGCATGTGTTCCGCTCTTAACTAGGC

GCTACGAGTAAAGGAGTCTCCCCTGATGGTTGTCCAGCCAGGGCACCACACATCTCCCCTGAAGTAGAGCTCCCAC

ACGGAGAAGGATTACCAATGGCAGACACTCTCTCAGCCTGTGAGGGCCAGTGGAAGGACGAGGAGCATAAACAC

ATTACAATTTCATTAACTACAGGCACCTACTCCCATTCATTTTGATTTTCTTATGTCCTGTTCCTTATTTTTTTAGGGGC

GGGCGGCCAGCCAGGAGCCAACTCAGACCTGAAACAAAAGCCACAATAATGTCGGTACTATAATTCTGGGTTTGAT

GCGGTGATTTCCTTCTGCATTCTGTCAGCAATGCCGGGGTACATTGTGGTGCCTCCGGACAGCACAGTGTTGGCG

CAGTTGTAGGGATACCACATTTTAACCAAGGTAGGTAGACCCTACATCAGTGGTTCCCAAACTTTTCCAGGCTAG

TGAGGGAAAGCCTGACCTCCAGTGTTGGAGCGAAGGTCAGCAGTGAAGCCAAAGGACTCATTCACAGGAAGATG

GTTTTTAATATAACTCATCTTACTTTTTTCATTTATTATTTTGTTGTTGTTGTTGTTGTTGCTGCTGCCGTTCTTTATTA

AGGGAGTCGGCCTCCACCTCCTGTCCCATGCGCTTCTCATTGAAGATGCGAGTCTTTTTCTCGTCATCAAACTCAA

ATACCAATCCTTACGGGTGAAGGGGTCAATCACCTTCTTCTTGGACCCCTTTTTGCCTGATTTGTTGAGTCCCTTG

GCAGTAACGGTAACATCTCGCCGGACGTCTCCCCATCTTGACCGTGCTACTTCTTCCTCTTCTCCTGCGTGAAAAAA

CACGACTCATACGGCGGTAACACAATTGGTGGCAGCGGTGGGAATGGTAGTGACAAACCCAATACTATACTTCTC

TCTCGCTTCATTAAGTGTGGAATTACTCCTGATGAGCTTGAGGACATCTACAAGAAGGCACAAGCCAACATCCGGG

ATTCAATGTTTTTTTTATGCAAGTAAATAAACAAATCGCAAACAAAAAAAAAAAAAAAATAAAAAAAAAAGGTTTA

CTTCATATCAGATGCTATTCTATTATTTTTTGCTTTATTGATATAAAGAAGACGGTGCTCATTGATAGCAAGGCGTT

CAATGATCTCGTACTCCTTGATAGGAAGTGAGAAAAGGTAGATCTCCTCCAGACTCCTAATCTTGCCGTCCTTCAC

TCAACAGGCCTAAGAGCAGGTTTACCCTTGCGGTGGTGTGTGTGGGACTTGAACACCGAGCCCGCGCCCTTCCTCT

AATTTCCTTACTTGAACCGCTAGTCTCCGCCGAGCTGACCAGCCACACGGCCAGCTTGGGGTGACGACAGATGTT

TTGTTCACAATGTAAGGAAGGAGCTTCTCAAGGTTGGGATTGGTCTCCAAGTGTCCTCGGATGGCCTTGCGCATCA
ATAGCTGGTTTGTTAAGGTAGATTAGCGCTATGTCGTTCGCTAAAGTCTTCCTGTCGTAGTAGGGATGTTTGGCGA
AGAGCTGGATCTATGAGTAAGGAGCGGTGTGAATAGTGGGGAGGGTTAGTGATCGTGCTGGTCCTCAGGTGGTA

TGGCTACCTGGGCACTCCATTCGGTCTCAGGAACAAAATGTATCCTCATAGGCTGAAGTTCTAAATAAACAAGTAAT

CAAACACGTTAATGATCCTTCCGCAGGTTCACCTACGGAAACCTTGTTACGACTTTTACTTCCTCTAAATGATCAAGT

GATCGATAGGCCCTGCTTTCGCAGTCCGTATTCGTACTGAAAATCAGGATCAAGCCAGCATTTGCCCTTTTGCTCTA
CACTACACCATCGGTAAGGAGGTCGTCGACCTGGTTCTTGATCGCATCCGAAAACTGGCCGACAACTGCACCGGAC
AGTCGCCTTGCACACAAACATGGAGTGTGGCAAAGACAATGAAATCCAGGAGTTACGAACATGGAATGACAAATG

TACCGTTCACTGTGATGGTGCCTGAGGGGTCTCGTTTGAAGGTGAGTGTCTTGCCGTTGAGGTTGGGGACTTGCA
TCGTCTGTTGTCTGGGAACTGAGTTGTGTTCCAGAAATTAGATCTTCCAGTTTCGTTAAGTGCAAGTCACCTTCAGT

GCTTTATAACTATTTCATATCAAGGCCCAAGATACATCAAAGTCACTGTTGTACGTCCACATGGAATATTAACACAT

GAGTGCTGCGATCGGCCCCAAGGTTGTACACTTCAGGAGGTATCGGAGTATTGTTTAACTATCAAGGAGCCTTACA
TAGGTATCTTAATAACACTTAAACCTGACATAAATAAATTTACTTTCTAACTCTTAGTTAAAACACTCTTTTATCATGA

AAGAGCGGCCTGGAGGGCATCATGGGCATTGCCCCATTCCTGCTGGCATGGAGCAGCAATGGCCTGCAGCACAAC

TGCTCTAATTGAGCCAGTAAGAGGCCAAGGCCTTATGTTTACAAGATGGTAGGGGTGGTGTCCGTGCGATGATGAC
GACTGATCATTGAAATCTTAATGATTATTGAACATTATGAACAGCAAAGTGTCTTGACTAATTATGTCACTTGGATGA

CTTCTTTAGTTGGGGTGTTACTCGGAAGTTGCAGATTTTGTCTAGTATCTCTGTGTTTCCGTCTTCTAACAGAGCATC

CCCCCAAGTTTGTCCAGCATCCATGACCTTGGAGCCACCAGGCGTTTGAGGTGGTGCTTTGGTCCTCGGGCCATGAC

GAATATTAGTACTATTTATTTATATGGCCTCTCTTGCTTCTAATGAAATATTCACAGCTTCTCTTGTAACTTTCTTTATT

TCCCATAACACAAAGTAGAATGTTAAAGCCTCCACGTTGAAAGGTGGCACGAGTTACATCGAAATGGCAAATGCAC

TACAGGTCCTTACGGATGTCCACGTCGCACTTCATAATGGAGTTGTAGGTGGTCTCGTGGATACCGCAGGATTCC[illegible]

[illegible]GCCCCTTGGCTAACAGGTGGATTCCTTGCGCTCCCCACCCCCCCCCACGCACACACACAGGCGAACACAAACTTT

AGCCTTGACAACAAACATGGGAGTACCCACCACCTGCATTTCCTCAAAGACAACACCTCGGCGCCTGTTGAGCAC[illegible]

[illegible]GAGCTTCTGGCACCCCGTTACGGGGTAGGAAACGTTTAACTTCATCGCGCCGTGCTGCCTCCCGTTCCCTAGCCA[illegible]

CCTTGCCTTGCCTGGTCTTGCCTTGCCTTGCCTTGCCTTGCCTTGCCTTGCCTTGCCTTGCCTTGCCTTGCCT[illegible]

[illegible]CTGATCCAATTGCCAAGGCCCGCCCAGAAAAGAAGGTGACTAAGAAGAGGTGGAATGCCAAGAGGCTTACCCGT

[illegible]GGCAGAAAGTCCTTTGAGGAACTGTTTGATAGTCAAAGGTTTGACATGCTTTGCACTCCCTTATTTATGATCAGC[illegible]

[illegible]AACCTGCCCAGCTTAGTGATGGGTGCCCACTCCTTCTCGTTCTCCTTCCCACGCCCACGGCCGCGGCCCCTACCAC

[illegible]GCCTTGCCTCAGCGTCTTACCTTGGTCGATATGGTAGTAGTAAGGTAGTGTGTTGGCTGCGTCTCAGAGCACATA[illegible]

ATGGTGTTCTTGCCCATGAGCACCACAGCACACCCACGCAGGGCCATGCGGATCTCCTGCATCTGCTTGGAGCCCA
CTGTAGCAATATTTCTCTCCTGCTGTCGTGGTGGTTGTGTTATACTTCCCGCCTGTGTGTTCCAAGGGCCTAGCAGA
TGTGGTGGCTGCCATTGGTGTGCTGTTGCCAGACAACATTCTGTTAGGGCCAGCATCCAACCCCTCCAGCTTGGG

TTGGACATCTTTCCGTCAGCTCGAGGCGCCGAGAGACCCAACGGCGAACCGAGGGCCCGGCATCCAAGAGCGCC

ACGCGAGGTTTCTGTCCACGCTGAGCTGGCCTTAGGACACCTGCGTTATCGTTTGACAGATGTACCGCCCCAGTCA
TGCAGGGCTTCCTTGTCTTCCACTCGTTCGGCGGCGGCACCGGCTCTGGCTTCACCTCGCTGCTGATGGAACGCCT
GAAGTAAAGAACGTCATCTGCTCTTTTGCCACTGGAATCTCTTTATTAATTCAAAGAGAAAATACTTAACCATTAG

GGCCGTTGGGTAAACTCATCCTGGGGTCGTCTGGCCTCACCTCCTCCTCCACCATGTAGTCAAGAAGGATCTCACG
AGGTTCCAGATCTTGCGGCTTTCCAGACTCCAGCGCATTCTGTTTACATGCACATTTTCCAGGACGCAAAGAATTA

CCCACGCCCTTGAGAACAACAACAACAACAACAACAACAACAACAACAACAACAACAACAACAACAACAACAACA

AGATCTATACTGGCTCAGAACAACACGCAGGTATAAGAGTTCAGACCATTAATGATTACAGCCTCAAAATTTTTCTCA
ACTACTTTTAAGCCTAGAGAAAATTAATTTTTATTTTTCAAGTATTATAACCCCCTTATTGAACAAGAAGCTAATCCCT

CCCTTCCTCCACGCTGGTTCTGGTACTTCATGAAGGTCTGGGCGTGGTCCCTCTCCTCATCACTAGATTCTTTGAAGA

CATTAGTTAATTTCTCTAGTATAAAGTGCTCTTAATACAGCAAATACATAAGATTGAATAATTGCAACAGCAGATTC
AAAAAAGGCAGTAGTTTTTTTTTTTCTTCAGAATATCAGTATGGCACACCCATGACATGCAAATTAGTTACCCACAA

CTTTTGCTTTATCGAACAATAGGTCCTTCAAGAAGCCTTCGCTCGGGGCAACCACTGCTATGACCACCACGAAGAGG

TTATACTTTTTTATTTCTAGGATTTACTTTTTTAATTACCCTTCTTGACCCTTTAATTACCTCTTACTTCTCTCTTGTTAG

GTTTCAATAGTTGCGGATGAAAGGCACACAAAATTAAGGATGCTAAGGGCACAGGTGAATTTTGACATGGCACCTT

ATACCCAGGAAGGAAGGCTGGAAGAGAGCCTCTGGGCAACGGAACCTCTCGTTGCCGATGGTGATCACCTGACCG

TTTTTTTGTTTATTTGTTTGGTATGCTTCAAATTGAAAACAAAGGCAAACACATTTTCTTTATATGGTGTGCTTCGAA

ACCGTAGATGCCACCTACTGCAGCCTCAGGACACTGAATCTCACAGAGGTAAACAGGCTCCTGGAGACGAGGTTGT

TGAAGAACGCAGGGCTGCTGTCAATGTCCGAAAGGATGCTTGGCTGGCCAAGATCGAAAAGGGAGAAGCTACCGT

AATGCTAGTGGACCAAAAATTCACCTTGCTTTCATCTCATATTTAGAGGATAGAGTTCTCCCATGAAGGAAATGGA

GCCCACGGCCGCGCCCCCTCCCCCGGGTGCCGAAGCCACCTCGGAAACCTCCACGGCCAGATCCGGAGTCTGCCA

TTAGGGTTGTGATAATTGAGTATTTCATGCCATGGAGAATTTCAATACCATTACCTAATTTACGTTTAGTCTCTATTA

CATTATCGGCACCCACGATGAAACAACGGCTGTATTCATCAAACAGCTGAGTGATCTTGGTGAAGTAGTTAGCCTT
AACTTTTAGTGTGGTGGTGGTGGTGGTGGTGATGCAGTGGGCAGCGGTAAGGACTAGTGATTCGTGTATTAGGGA

CAGTCCGAAGGCCTCACTAAATCATTCAATCGGTAGTAGCGACGGGCGGTGTGTACAAAGGGCAGGGACGTAATC

AACTCCCCACCTGATAATGTCCTCGGAGCGGATCGCGGCGGGCGCGAACGCCCGGTCAAGCTAACGCGGAGCCGA
CTCCGTCGACTACGGCAAGAAGAGCAAACTGGAGTTCGCCATCTACCCGGCGCCCCAGGTGGCCACGGCCGTGGT
TATGTCGATGTCTTTCAAGCCACAAAGTCAGTCCTCAGGTTAGAATCCGCTACTCCTTCCCACAGGAGCGGGTCAGC

CAGCAGGTTGAAGTACTGAGAGGCATACAGTGGGTGGGGGGAATCTTGTGGGACCCAGTTCATAACCTCAATGTA
CTACAACCAAATCCAAAATTAGGCTTTAATAAATTATTTGATTGCAAGGTTGAATATGAATGATCTTTCTCATCTTCA

ACTCTGGCGCTATGAAGGTCTTAGGACCTTCGTTACCTCTCACGACCTGGATGCCTGCCTCC
