# Supplementary figures and images for: Seasonal changes in the expression of insulin-like androgenic hormone (IAG) in the androgenic gland of the Jonah crab, Cancer borealis
Source: PLoS One. 2022 Feb 3;17(2):e0261206. doi: 10.1371/journal.pone.0261206 (PMC8812979; doi:10.1371/journal.pone.0261206)

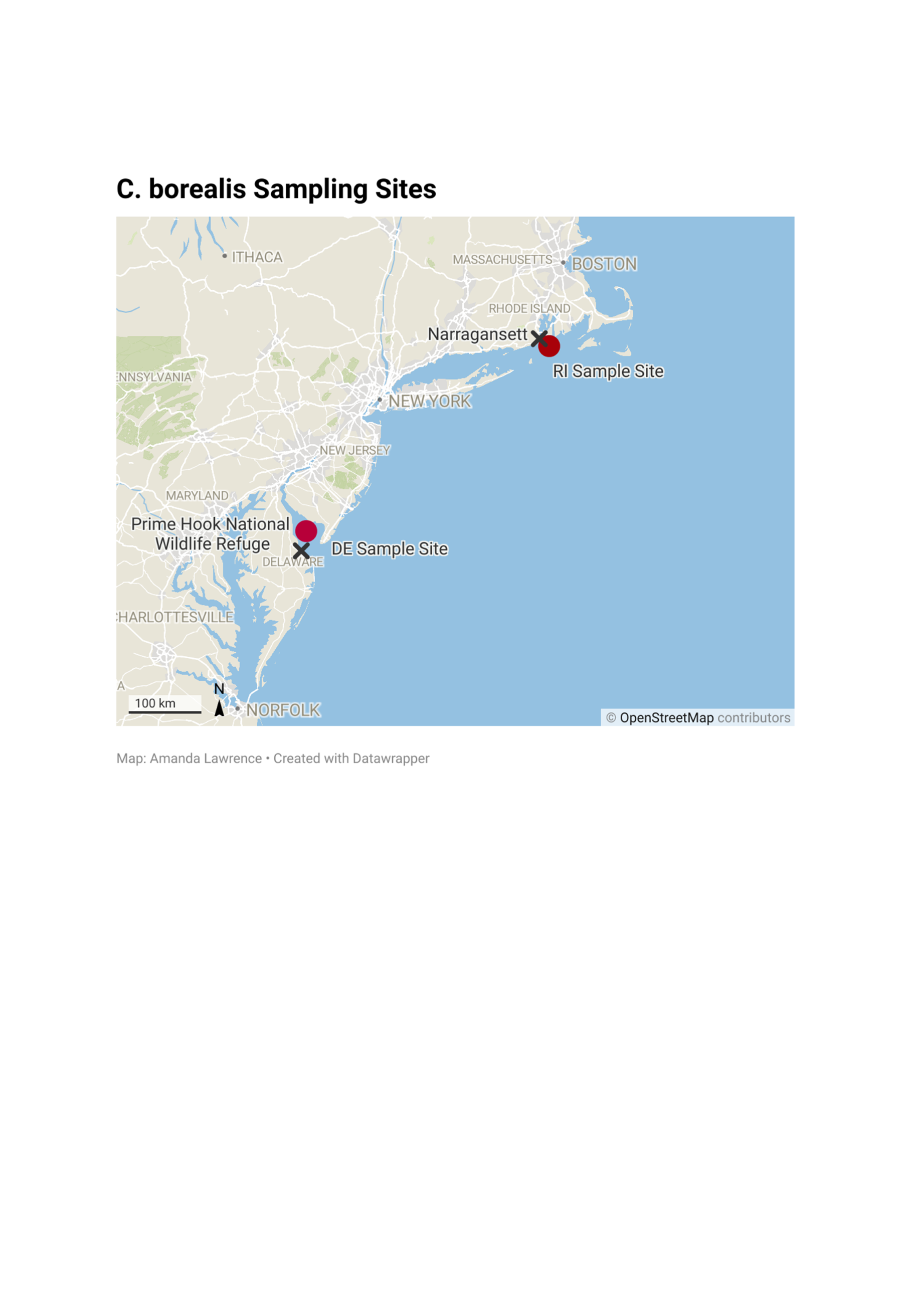

Supplement: S1 Fig — A volcano plot shows overall differential expression patterns where the X-axis reflects difference in expression between the eyestalk ablated males on the left and the non-ablated control males on the right. The Y-axis reflects the False Discovery Rate, a measure of differential expression. The top 100 most differentially expressed transcripts are marked with a solid square for the upregulated ones and a dotted square for the downregulated genes. Group numbers are presented using K-means clustering (K = 6) results. Upregulated are the groups 3 (circle), 4 (square), and 5 (remaining dots); Downregulated are the groups 1 (square), 2 (circle), and 6 (remaining dots). * = PPOAE; ** = trypsin-like enzyme; and *** = chymotrypsin. (TIF) [file pone.0261206.s002.tif]

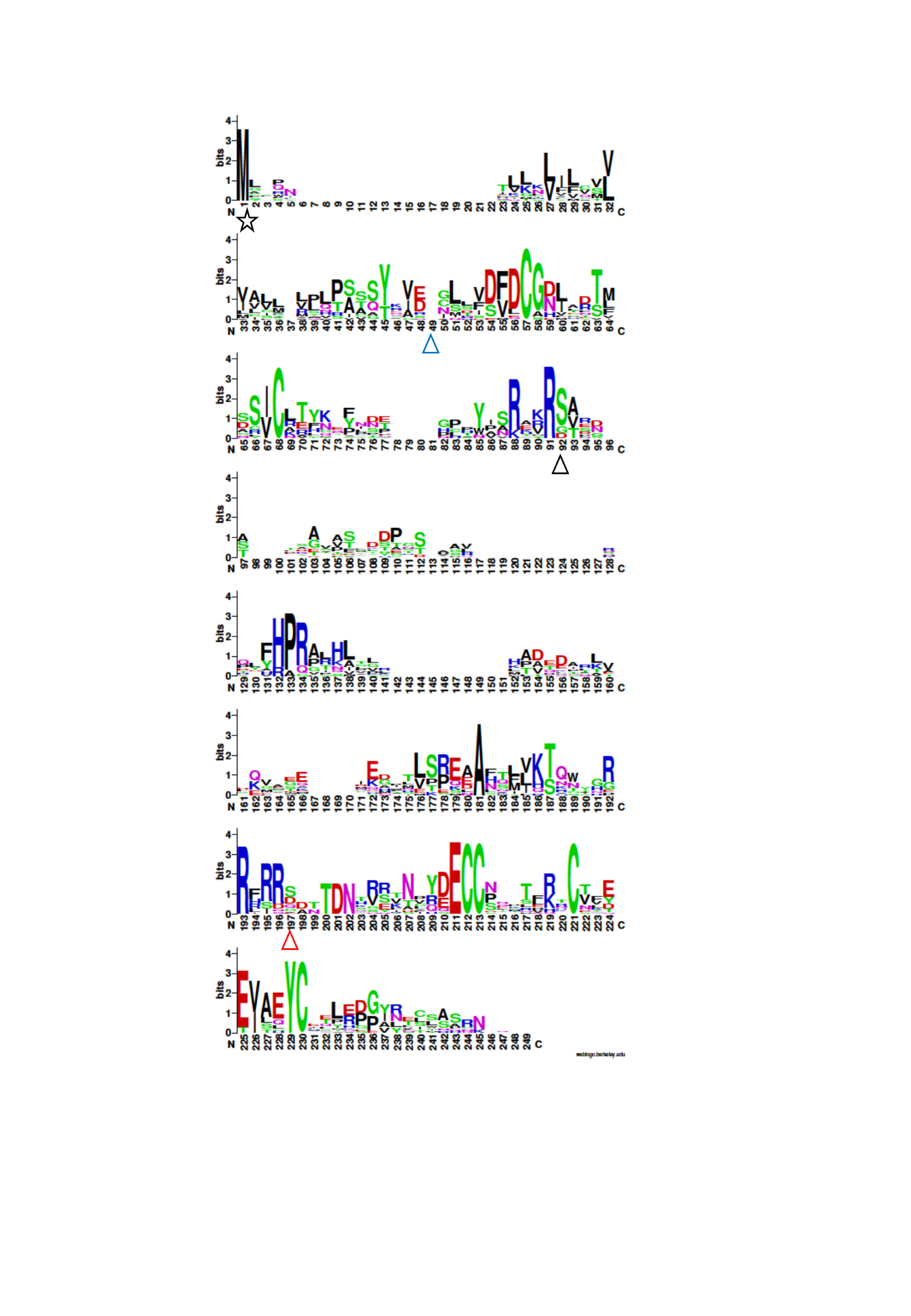

Supplement: S2 Fig — A sequence logo of MUSCLE 3.8.31 aligned decapod IAG and isopod AGH deduced amino acid sequences for the entire ORF region using https://weblogo.berkeley.edu/logo.cgi. The Y-axis describes the amount of information in bits*, and the X-axis shows the position in the alignment. The start of the signal peptide is signified by a black outlined star, the start of the B chain is marked by a blue outlined triangle, the C peptide is marked by a black outlined triangle, and A chain is marked by a red outlined triangle. The BLAST search identified a candidate IAG sequence from the top 100 ranked first in ablated and second in intact based on TPM values. The ablated/intact TPM ratio of 2.0 revealed that the IAG candidate gene was twice as abundant in ablated than intact males. The candidate IAG sequence was used to generate GSPs, allowing the full-length isolation of CabIAG via cloning from the AG, resulting in a cloned and transcriptomic CabIAG sequence sharing 98.7% identity. (TIF) [file pone.0261206.s003.tif]

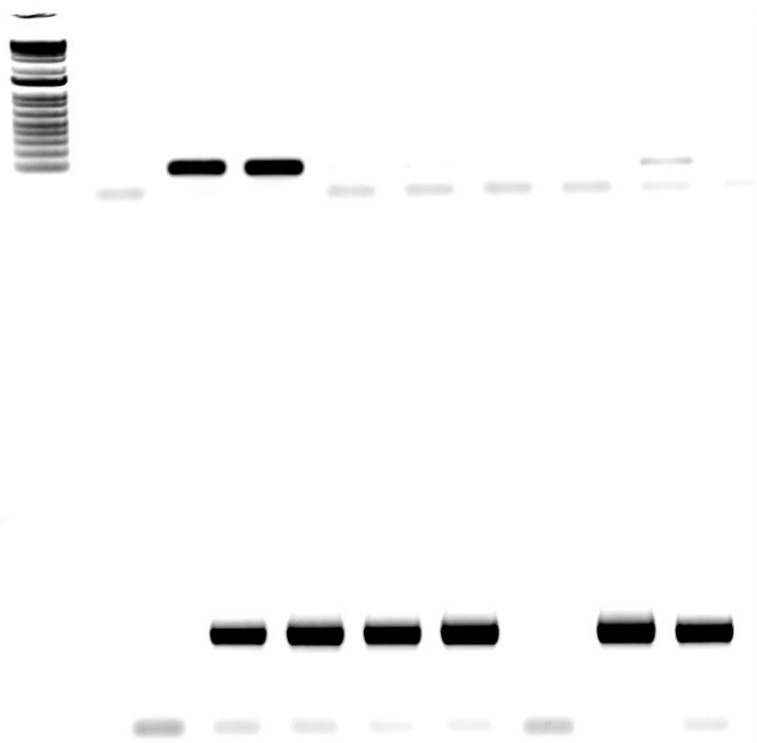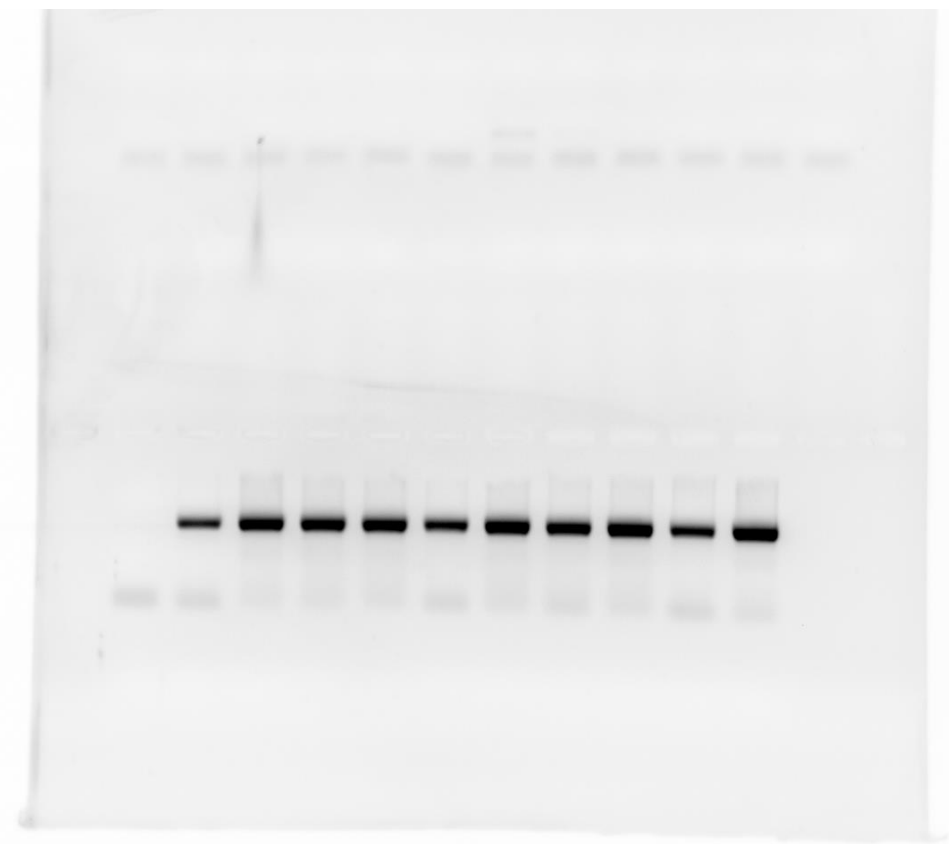

Supplement: S3 Fig — (PDF) [file pone.0261206.s004.pdf]
