# Supplementary material for: Seasonal changes in the expression of insulin-like androgenic hormone (IAG) in the androgenic gland of the Jonah crab, Cancer borealis
Source: PLoS One. 2022 Feb 3;17(2):e0261206. doi: 10.1371/journal.pone.0261206 (PMC8812979; doi:10.1371/journal.pone.0261206)

Male:
1.no template control
2. AG
3. AG from an eyestalk-ablated individual
4. eyestalk ganglia
5. thoracic ganglia complex;
6. brain
7. hemocytes
8. testis

Female:
1. no template control
2. spermatheca
3. eyestalk ganglia
4. thoracic ganglia complex
5. chela muscle
6. hemocytes
7. Hepatopancreas
8. ovary. *CabNa/K-ATPase*

A. *CabIAG*

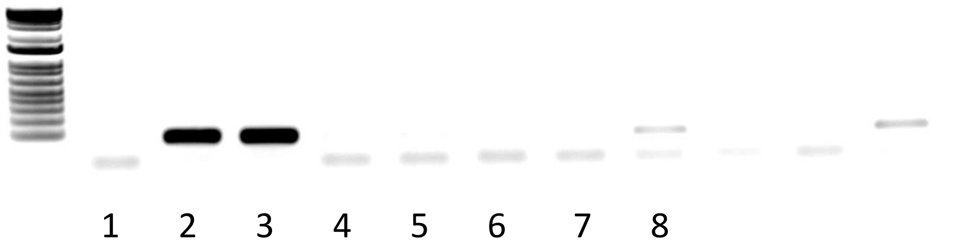

B. *CabNa/K-ATPase*

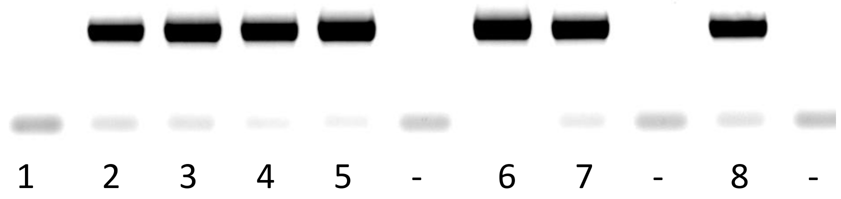

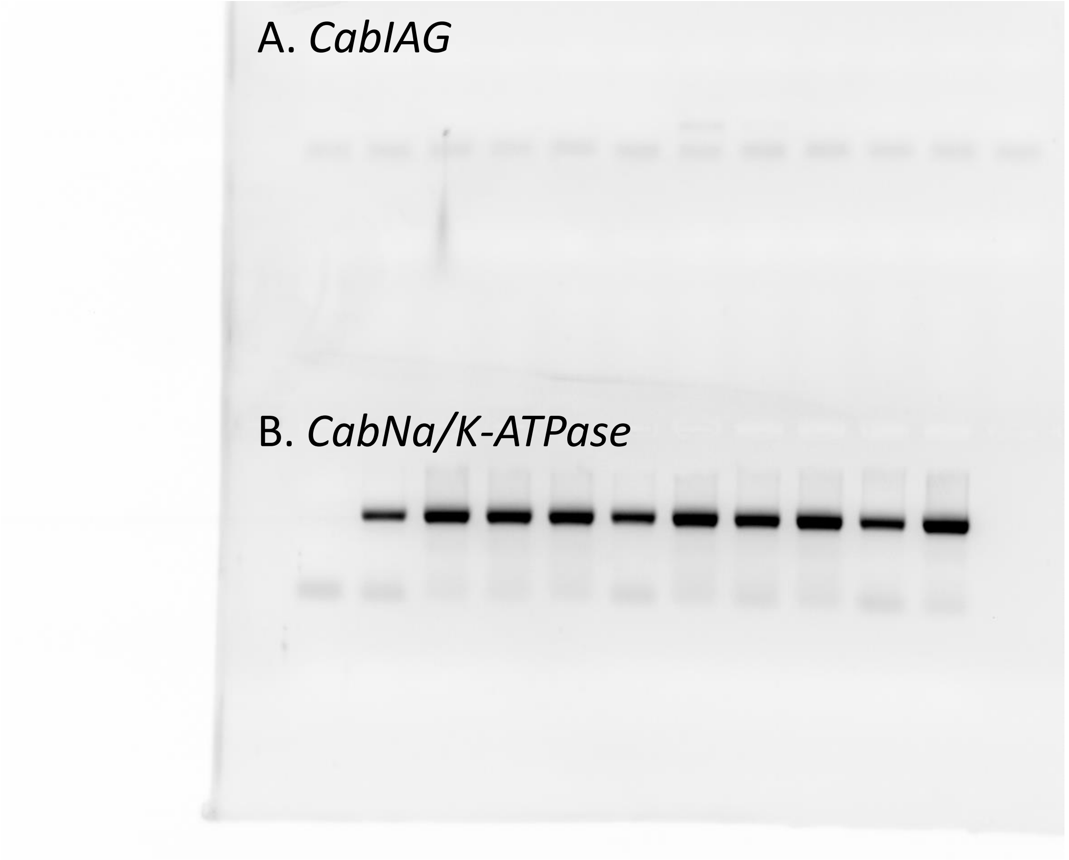

Supplement: S1 Raw images — (PDF) [file pone.0261206.s008.pdf]
